# Supplementary material for: A robust, semi-automated approach for counting cementum increments imaged with synchrotron X-ray computed tomography
Source: PLoS One. 2021 Nov 4;16(11):e0249743. doi: 10.1371/journal.pone.0249743 (PMC8568193; doi:10.1371/journal.pone.0249743)
Supplement: S1 File — (DOCX) [file pone.0249743.s001.docx]

**Supplement for:**

**A robust, semi-automated approach for counting cementum increments imaged with synchrotron X-ray computed tomography**

Elis Newham^1,2^, Pamela G. Gill.^3,4^, Kate Robson Brown^5,6^, Neil J. Gostling^7^, Ian J. Corfe^8,9^, & Philipp Schneider^1,10*^.

1. Bioengineering Science Research Group, Faculty of Engineering and Physical Sciences, University of Southampton, Southampton, United Kingdom.
2. School of Engineering and Materials Science, Queen Mary University of London, London, E1 4NS, United Kingdom.
3. School of Earth Sciences, University of Bristol. Wills Memorial Building, Queens Road, Bristol, BS8 1RJ, United Kingdom.
4. Department of Earth Sciences, Natural History Museum. Cromwell Road, London, SW7 5BD, United Kingdom.
5. Department of Mechanical Engineering, Queen’s Building, University of Bristol, Bristol, UK.
6. Department of Anthropology and Archaeology, University of Bristol. 43 Woodland Road, Bristol, BS8 1UU, United Kingdom.
7. School of Biological Sciences, Faculty of Environmental and Life Sciences, University of Southampton, Southampton, UK.
8. Developmental Biology Program, Institute of Biotechnology, University of Helsinki. Viikinkaari 5D, University of Helsinki, Helsinki, Finland.
9. Geological Survey of Finland, Espoo, Finland.
10. High-Performance Vision Systems, Center for Vision, Automation & Control, AIT Austrian Institute of Technology, Vienna, Austria.
11. **Image processing by directional filtering**

Once straightened and isolated, the cementum data can be further processed using directional filters in order to enhance contrast between cementum increments. Image filtering is commonly used to suppress the contribution of unwanted signals such as noise, while preserving and enhancing the targeted signal or image contribution for the analysis in question. A wealth of image filters exist in the literature and are commonly available in image processing and analysis software packages. Several can be tuned and tweaked for data-specific purposes, including oriented filters such as the steerable Gaussian filter.

Oriented filters are a class of filters in which a filter of arbitrary orientation is synthesised as a linear combination of a set of ‘basis filters’. Assuming that the following Gaussian function (*G*) for a set of coordinates *x* and *y* that is circularly symmetric:

|  | $G\left( x,y \right)= e^{-\left( x^{2}+y^{2} \right)}$. | (1) |
| --- | --- | --- |

The directional derivative operator (*G*_i_) of this function is steerable (Freeman and Adelson, 1991). This can be described using a rotation operator $\left( x,y \right)^{\theta}$. This operator can be applied to any function $f\left( x,y \right)$ so that $f^{\theta}\left( x,y \right)$ represents $f\left( x,y \right)$ rotated by an angle $\theta$ about the origin. Hence, the first derivative of a Gaussian ($G_{1}^{0^{o}}$) is given by:

|  | $G_{1}^{0^{o}}= \frac{\delta}{\delta x}e^{-\left( x^{2}+y^{2} \right)}= -2xe^{-\left( x^{2}+y^{2} \right)}$. | (2) |
| --- | --- | --- |

The same function rotated by 90° is given by:

|  | $G_{1}^{{90}^{o}}= \frac{\delta}{\delta y} e^{-\left( x^{2}+y^{2} \right)}= -2ye^{-\left( x^{2}+y^{2} \right)}$. | (3) |
| --- | --- | --- |

On this account, the filter $G_{1}$ at an arbitrary orientation $\theta$ can be formulated by taking the linear combination of $G_{1}^{0^{o}}$and $G_{1}^{{90}^{o}}$:

|  | $G_{1}^{\theta^{o}}=\cos\left( \theta\right)G_{1}^{0^{o}}+sin(\theta)G_{1}^{{90}^{o}}$ , | (4) |
| --- | --- | --- |

where $G_{1}^{0^{o}}$ and $G_{1}^{{90}^{o}}$ form the basis filters for any arbitrary rotation. The $\cos\left( \theta\right)$ and $sin(\theta)$ terms are the corresponding interpolation functions of these basis filters (Freeman and Adelson, 1991).

Straightening of cementum increments allows a single orientation to be used when filtering (e.g. $G_{1}^{{90}^{o}}$ for increments along the *x*-axis or $G_{1}^{0^{o}}$ for increments along the *y*-axis), and steerable Gaussian filtering has been shown here to substantially enhance image contrast between straightened cementum increments (Fig. 5d). We used a steerable Gaussian filter implemented in MATLAB (R2016a; The MathWorks, Inc., Natick, MA, USA), namely the tool called ‘SteerGauss’ (version 1.0.0.0) developed and made freely available by Lanman (Lanman, 2006; [www.mathworks.com/matlabcentral/fileexchange/9645-steerable-gaussian-filters](http://www.mathworks.com/matlabcentral/fileexchange/9645-steerable-gaussian-filters) [accessed on September 01, 2021]). Applying a directional filter with an orientation along the *y*-axis and a directional derivative of 1 isolates the first derivative of a cementum image and creates a separate image where light increments are given greyscale values of 255 and dark increments zero as shown in Fig. S1.b & S1.e. The addition of this directional derivative image to the original image provides a filtered image with increased the image contrast between the cementum increments (Fig. S1.c,f). However, the addition of a directional derivative image to the original image can lead to the loss of information due to the oversaturation of the resultant image file (grey values > 256 in 8-bit data or >65,536 in 16-bit data), and original textures and contrast within increments can be significantly reduced (Fig. S1.e). This problem can be mediated by dividing the greyscale values of the directional derivative image by a fixed value. As it is essential that the imaged structures are preserved (e.g. texture expressed as local variations of greyscales, Fig. S1,f) for subsequent analyses, this was here performed by converting the derivative image into a double precision format, and dividing all values by a factor between two and 10.

The effects of filter parameters on image contrast and texture were analysed by measuring and comparing greyscale distributions along 10 transects through the cementum of the l14 specimen (Fig. S1.d). Distributions were measured along the same transects after adding first directional derivative images that had been divided incrementally by integers from two to 10. These resulting ‘filtered’ datasets are here assigned to a naming convention following ‘Filter 2’, ‘Filter 3’, …, ‘Filter 10’, with the number describing the integer with which the first directional derivative image was divided by (Table S1). The modulation transfer function (MTF) of cementum increments in each transect was used to provide a quantitative estimate of image contrast, and how this changed due to the strength of filtering. The effects of filtering on discrete aspects of the greyscale distribution in each transect were also compared to account for potential loss of texture detail.

The MTF can be described by the hypothetical greyscale imaging of a square wave function composed of light and dark increments. The maximum greyscale possible will originate from the light increments, and the minimum from the dark increments. The modulation of the original function can be defined as:

|  | $M_{O} = \frac{\left( L_{\max}- L_{\min} \right)}{\left( L_{\max}+ L_{\min} \right)}$ , | (1) |
| --- | --- | --- |

where $L_{\max}$ describes the maximum greyscale (‘luminance’) value of the light increments, and $L_{\min}$ describes the minimum greyscale value of the dark increments. If the frequency (*v*) and the ‘modulation’ (contrast) of the original data is known, then the modulation of the subsequent image can be measured, and the MTF of the image can be calculated as the ratio between the modulation of the original function ($M_{o}$) and the modulation of the filtered image ($M_{i}$):

|  | $\mathrm{MTF}\left( v \right)= \frac{M_{i}}{M_{o}}$. | (2) |
| --- | --- | --- |

For this study, the MTF was used to provide quantitative information on the amount of contrast added by using a steerable Gaussian filter of increasing strength. For each filter, modulation functions were calculated for each increment pair along the same 10 transects as unfiltered data, and MTFs subsequently calculated and compared using ANOVA in the ‘Past 3’ Statistical environment (Hammer et al., 2001).

The potential loss of signal from filtering was assessed through comparing the greyscale distribution of each transect when imaged under each filter strength. Increment pairs were identified visually comparing greyscale transects to the unfiltered CT images from which they were produced. Primary light increments were defined as peaks in greyscale that matched with visible light increments in PPCI SR CT data, and primary dark increments were defined as troughs in greyscale that matched with visible dark increments in the data (Fig. S1.f). After primary increment pairs had been identified along each transect during MTF analysis, the number of secondary peaks in greyscale was recorded within each increment pair, along with their greyscale value and position (Fig. S1.f). Secondary/’piggy-back’ increments were defined as minor incremental features found along the ascending/descending limbs of primary increment pairs and troughs. ANOVA comparisons were performed between the number of secondary peaks imaged using each filter and the number of peaks for images that have not been filtered, as well as the ratio of their greyscale value with that of the corresponding primary peak. This was in order to identify whether certain filter strengths created a significant loss in detail of increments, which may limit future analyses and comparisons between cementum increments.

ANOVA comparisons of MTF values suggested that dividing the first directional derivative of an image by a factor between seven and 10 does not significantly improve image contrast relative to unfiltered values (Table S1). Dividing by smaller values steadily improve image contrast until $M_{i}$ values of cementum increments become an order of magnitude larger than unfiltered values when the first directional derivative is divided by between two and four (Fig. S1.g). This suggests that a minimum division of six, and an optimum division of less than four should be used for the targeted steerable Gaussian filter.

Following MTF measurements, ANOVA comparisons suggested that the strength of filtering had no significant effect on the number of secondary peaks within primary cementum increments. Although filtering affected the absolute height of primary peaks and secondary peaks (Fig. S1.f), their magnitudes were changed proportionally, thus preserving their ratio between them even when using the strongest filters. Finally, in visual analysis of transects, the strongest filter (‘Filter 2’) provided the clearest increment pairs, shown by discrete peaks and troughs in absolute greyscale values, directly corresponding to principal light and dark increments, respectively (Fig. S1.f). For weaker filters (‘Filter 3’, ‘Filter 4’, etc.) these patterns became concealed by secondary peaks and troughs, and thus harder to distinguish. This is reflected when using the automated increment counting algorithm to count increment pairs: The strongest filter (‘Filter 2’) provided the most consistent counts for every measured transect (Table S2).

Results of filter comparisons thus suggested that the first directional derivative of an image should be halved before being added to the original cementum image, in order to optimise contrast between cementum increments and improve the accuracy of automated cementum increment counting. As a MATLAB function this can be incorporated into an automated image-processing framework, to be employed after straightening of cementum increments. This combination of straightening and filtering has been shown here to significantly enhance image quality of cementum increments, and to provide data that can be readily examined and automatically be processed in subsequent quantitative analyses.

As a final test, we applied the filtering function to a subsample of five specimens (k16, l10, k91, l56 and l59) and saved the outputs as both 8-bit and 16-bit tiff files, to determine whether the increased range of absolute greyscale values offered by 16-bit data provides greater image contrast between straightened increments, and improved accuracy of increment counts (compared to 8-bit data). The original results of the filtering algorithm are saved as double precision/float files, so conversion to tiff format required adaptation of the dynamic range covered by the result to the range specified by the bit depth (8 vs. 16). Comparison between the accuracies of increment counts provided by both datasets for each specimen suggests that 16-bit data does not provide a significantly higher accuracy than that of 8-bit data, as both datasets provided mean increment count estimates within the maximum/minimum expected counts for each specimen (Fig. S2). ANOVA comparisons between 8-bit and 16-bit datasets for each specimen only suggest a significant difference in estimated increment counts for the l10 specimen, and Levene’s tests do not find significant differences in the variances of these datasets (Table S3). This suggests that, while a minority of specimens may produce different ranges of absolute increment estimate values using 8/16-bit data, they are still centred on the same increment count and 16-bit data does not provide significantly higher accuracy or precision for increment count estimates than 8-bit data.

| **Filter** | **ANOVA F-value** | **ANOVA *p*-value** |
| --- | --- | --- |
| **Filter 2** | 167.20 | <0.01 |
| **Filter 3** | 81.62 | <0.01 |
| **Filter 4** | 59.70 | <0.01 |
| **Filter 5** | 31.59 | <0.01 |
| **Filter 6** | 15.67 | <0.01 |
| **Filter 7** | 5.64 | 0.20 |
| **Filter 8** | 1.38 | 0.24 |
| **Filter 9** | 1.05 | 0.27 |
| **Filter 10** | 0.90 | 0.36 |

**Table S1. ANOVA comparisons of the modulation transfer functions for the steerable Gaussian filters**. Cementum of PPCI SR CT slice processed by steerable Gaussian filters of increasing strength (‘Filter 2’: strongest, ‘Filter 10’: weakest) are compared to the original image (i.e. no filtering) using ANOVA. Data processed is from specimen l56.

| **Transect** | **Increment count** | | |
| --- | --- | --- | --- |
|  | **Filter 2** | **Filter 3** | **Filter 4** |
| 1 | 10 | 12 | 10 |
| 2 | 10 | 10 | 13 |
| 3 | 10 | 9 | 9 |
| 4 | 9 | 9 | 10 |
| 5 | 10 | 11 | 10 |
| 6 | 10 | 10 | 9 |
| 7 | 10 | 9 | 10 |
| 8 | 10 | 10 | 10 |
| 9 | 9 | 8 | 9 |
| 10 | 10 | 11 | 11 |

**Table S2. Algorithmically generated counts of cementum increments for different steerable Gaussian filters.** Cementum increment counts derived from CT images processed by steerable Gaussian filters of different strengths along 10 transects of individual l56. Filter 2 provided most consistent increment counts. Data processed is from specimen l56.

| **Specimen** | **Levene's test (*p*)** | **ANOVA (*p*)** |
| --- | --- | --- |
| k16 | 0.85 | 0.61 |
| l10 | 0.08 | 0.05 |
| k91 | 0.06 | 0.70 |
| l56 | 0.61 | 0.46 |
| l59 | 0.41 | 0.63 |

**Table S3.** Results of statistical comparisons between estimated increment counts obtained from 8-bit vs. 16-bit datasets, generated from the steerable Gaussian filtering function applied to five specimens. Alpha value for *p* = 0.05.

1. **Robustness testing for increment counting algorithm**

To test the robustness of the proposed increment counting algorithm, it was applied to arbitrary sine wave patterns of known periodicity that modelled the peak/trough patterns in greyscale values created by cementum increments along trajectories through the width of the cementum. Wave/increment numbers between five and 30 were investigated. For each count, a sine wave pattern was created in MATLAB using the ‘sin’ command (Fig. S2.a). This pattern was disturbed in a controlled manner to mimic increasingly severe image noise, by adding noise to the original sine wave pattern using the MATLAB ‘randn’ function (Fig. S2.b). The multiplication of the random values making up these noise patterns by increments between one and 10 allowed controlling the amount of noise that was applied to the original sine wave pattern (Fig. S2.b). This resulted in signal-to-noise ratios (SNRs) between 0.9 (when the noise pattern was multiplied by a factor of one), to 0.1 (when the noise pattern was multiplied by a factor of 10). In other words, noise was added incrementally of increasing severity to produce SNRs that decreased in 0.1 decrements between an SNR of 0.9 and 0.1 (Fig. S2.c). 30 sine wave patterns were generated for each SNR. The increment counting algorithm was then applied to each pattern and the resulting increment count estimate saved. This was repeated for each increment/wave count between 5 and 30.

**3. Splitting of transects through cementum**

Our increment counting algorithm is dependent on splitting a single transect of averaged greyscale values through the cementum into individual sections. This is necessary to minimise the effects on increment counting, due to large-scale drift in greyscale values from low to high, from the outer cementum surface (lower greyscale values) to the cemento-dentine boundary (higher greyscale values) (Fig. 2). The choice of the number of sections is based on a comparison we performed between three, five and seven sections. To this end, in trials of our increment counting algorithm, we generated sine wave transects of known increment count between five and 30, and decreasing signal-to-noise ratio (SNR) from 0.9 to 0.1 to test for the robustness of the algorithm depending on the number of transect sections. The accuracy of the estimated counts was assessed by comparing their mean rounded value to the known count for each sine wave, where the precision of their counts was assessed by measuring and comparing their standard deviations. A standard deviation of one integer (i.e. two whole numbers) or more around the mean was determined as an imprecise value, as this has the potential to provide an incorrect estimate of annual increment counts.

The estimated increment count was 100% accurate and independent of the number of transect sections for un-modified sine wave patterns of known increment count (Tables S3-S4). For sine wave patterns with added noise, application of our increment counting algorithm to patterns with SNRs of < 0.8 created several increment count estimates whose means underestimated known counts by > 1 when transects were split into seven sections (Table S5). Thus, no further testing of lower SNR values was performed for sine wave transects split into seven sections.

Transects split into three sections were more resistant to noise than those split into seven sections, and SNRs of < 0.8 produced identical results to un-modified patterns (Table S4). SNRs of between 0.8 and 0.6 provided estimated increment counts with smaller standard deviations than when transects were split into five sections, with values for no transect exceeding one integer (Fig. 5a, Table S4). Increment count estimates provided for SNRs of 0.4 exhibit larger standard deviations when transects are split into three sections, compared to five sections (Fig. 5b, Table 3). The accuracies of estimates were also lower when transects are split into three sections than when split into five sections, and the estimated increment count for the sample with known counts of 29 increments was 28. For SNRs of 0.2, application of our algorithm to transects split into three sections underestimated known increment counts by at least one integer for several counts (Table S4).

In summary, lower number of transect sections provided lower standard deviations of increment count estimates. Each section will provide its own uncertainty for its estimated counts, and this uncertainty will be increasingly compounded with increasing numbers of sections created per transect. However, as noise increases along transects, an optimum number of sections are required to accommodate this noise. When a noisy transect is split into an insufficient number of sections, the amplification of standard deviations of greyscale values over each large section is artificially amplified by this noise. This can lead to underestimation of their increment counts, as genuine increments may not depart from the mean greyscale value beyond these amplified lower/higher standard deviation values. This highlights the need to assess an optimum value for splitting transects through the cementum, and a value of five was found as optimum for this study.

| **Known increment count** | **SNR = 0.8** | | | | **SNR = 0.6** | | | | **SNR = 0.4** | | | | **SNR = 0.2** | | | |
| --- | --- | --- | --- | --- | --- | --- | --- | --- | --- | --- | --- | --- | --- | --- | --- | --- |
|  | **Mean of estimated counts** | **Standard deviation** | ***CV*** | **Increment count (rounded mean of estimates)** | **Mean of estimated counts** | **Standard deviation** | ***CV*** | **Increment count (rounded mean of estimates)** | **Mean of estimated counts** | **standard deviation** | ***CV*** | **Increment count (rounded mean of estimates)** | **Mean estimated count** | **standard deviation** | ***CV*** | **Increment count (rounded mean of estimates)** |
| **10** | 10.00 | 0.00 | 0.00 | 10 | 10.03 | 0.31 | 0.03 | 10.00 | 10.42 | 0.56 | 0.05 | 10.00 | 10.56 | 1.32 | 0.12 | 11.00 |
| **11** | 11.00 | 0.00 | 0.00 | 11 | 10.95 | 0.65 | 0.06 | 11.00 | 11.29 | 0.78 | 0.07 | 11.00 | 11.64 | 1.71 | 0.15 | 12.00 |
| **12** | 12.00 | 0.00 | 0.00 | 12 | 12.03 | 0.41 | 0.03 | 12.00 | 12.23 | 0.72 | 0.06 | 12.00 | 11.93 | 0.93 | 0.08 | 12.00 |
| **13** | 13.00 | 0.00 | 0.00 | 13 | 12.63 | 0.49 | 0.04 | 13.00 | 13.26 | 0.44 | 0.03 | 13.00 | 13.27 | 1.34 | 0.10 | 13.00 |
| **14** | 14.00 | 0.00 | 0.00 | 14 | 13.97 | 0.66 | 0.05 | 14.00 | 14.16 | 1.34 | 0.09 | 14.00 | 14.01 | 1.18 | 0.08 | 14.00 |
| **15** | 15.00 | 0.00 | 0.00 | 15 | 14.73 | 0.51 | 0.03 | 15.00 | 14.71 | 0.74 | 0.05 | 15.00 | 14.90 | 1.67 | 0.11 | 15.00 |
| **16** | 16.00 | 0.00 | 0.00 | 16 | 15.80 | 0.60 | 0.04 | 16.00 | 16.19 | 0.54 | 0.03 | 16.00 | 16.00 | 1.63 | 0.10 | 16.00 |
| **17** | 17.00 | 0.00 | 0.00 | 17 | 16.83 | 0.45 | 0.03 | 17.00 | 17.00 | 0.68 | 0.04 | 17.00 | 16.60 | 1.05 | 0.06 | 17.00 |
| **18** | 18.00 | 0.00 | 0.00 | 18 | 17.80 | 0.48 | 0.03 | 18.00 | 17.65 | 0.66 | 0.04 | 18.00 | 17.90 | 1.34 | 0.07 | 18.00 |
| **19** | 19.00 | 0.00 | 0.00 | 19 | 18.93 | 0.36 | 0.02 | 19.00 | 18.74 | 0.77 | 0.04 | 19.00 | 19.02 | 0.88 | 0.05 | 19.00 |
| **20** | 20.00 | 0.00 | 0.00 | 20 | 19.67 | 0.65 | 0.03 | 20.00 | 19.65 | 0.71 | 0.04 | 20.00 | 19.86 | 0.85 | 0.04 | 20.00 |
| **21** | 21.00 | 0.00 | 0.00 | 21 | 20.57 | 0.62 | 0.03 | 21.00 | 21.03 | 0.84 | 0.04 | 21.00 | 19.83 | 1.50 | 0.08 | 20.00 |
| **22** | 22.00 | 0.00 | 0.00 | 22 | 21.63 | 0.66 | 0.03 | 22.00 | 21.58 | 0.92 | 0.04 | 22.00 | 21.37 | 1.12 | 0.05 | 21.00 |
| **23** | 23.00 | 0.00 | 0.00 | 23 | 22.73 | 0.63 | 0.03 | 23.00 | 23.45 | 0.80 | 0.03 | 23.00 | 20.10 | 1.89 | 0.09 | 20.00 |
| **24** | 24.00 | 0.00 | 0.00 | 24 | 23.67 | 0.65 | 0.03 | 24.00 | 23.81 | 1.15 | 0.05 | 24.00 | 23.17 | 0.98 | 0.04 | 23.00 |
| **25** | 25.00 | 0.00 | 0.00 | 25 | 24.93 | 0.68 | 0.03 | 25.00 | 25.35 | 1.14 | 0.04 | 25.00 | 24.20 | 1.15 | 0.05 | 24.00 |
| **26** | 26.00 | 0.00 | 0.00 | 26 | 25.70 | 0.46 | 0.02 | 26.00 | 26.42 | 1.05 | 0.04 | 26.00 | 24.57 | 1.12 | 0.05 | 25.00 |
| **27** | 27.00 | 0.00 | 0.00 | 27 | 26.60 | 0.56 | 0.02 | 27.00 | 27.32 | 1.12 | 0.04 | 27.00 | 25.93 | 1.47 | 0.06 | 26.00 |
| **28** | 28.00 | 0.00 | 0.00 | 28 | 27.83 | 0.81 | 0.03 | 28.00 | 27.58 | 1.11 | 0.04 | 28.00 | 26.90 | 1.03 | 0.04 | 27.00 |
| **29** | 29.00 | 0.00 | 0.00 | 29 | 29.13 | 0.78 | 0.03 | 29.00 | 28.45 | 2.23 | 0.08 | 28.00 | 28.67 | 2.51 | 0.09 | 29.00 |
| **30** | 30.00 | 0.00 | 0.00 | 30 | 29.63 | 0.80 | 0.03 | 30.00 | 29.52 | 2.81 | 0.10 | 30.00 | 28.70 | 1.89 | 0.07 | 29.00 |

**Table S4. Results of application of our increment counting algorithm to sine wave patterns of known increment counts.** Transects have been split into three sections, with increasing amounts of noise applied (indicated by signal-to-noise ratio values; SNR). *CV*: coefficient of variation.

| **Known increment count** | **SNR = 0.8** | | | |
| --- | --- | --- | --- | --- |
|  | **Mean of estimated counts** | **Standard deviation** | ***CV*** | **Increment count (rounded mean of estimates)** |
| **10** | 10.13 | 1.41 | 0.14 | 10 |
| **11** | 11.43 | 1.17 | 0.10 | 11 |
| **12** | 12.03 | 1.03 | 0.09 | 12 |
| **13** | 12.90 | 0.71 | 0.06 | 13 |
| **14** | 13.97 | 1.03 | 0.07 | 14 |
| **15** | 15.40 | 0.97 | 0.06 | 15 |
| **16** | 16.20 | 0.81 | 0.05 | 16 |
| **17** | 16.33 | 0.99 | 0.06 | 16 |
| **18** | 17.90 | 1.12 | 0.06 | 18 |
| **19** | 18.63 | 1.03 | 0.06 | 19 |
| **20** | 20.27 | 1.74 | 0.09 | 20 |
| **21** | 21.33 | 0.84 | 0.04 | 21 |
| **22** | 22.27 | 1.74 | 0.08 | 22 |
| **23** | 23.83 | 0.38 | 0.02 | 24 |
| **24** | 24.33 | 0.48 | 0.02 | 24 |
| **25** | 25.30 | 0.53 | 0.02 | 25 |
| **26** | 26.57 | 0.68 | 0.03 | 27 |
| **27** | 27.47 | 0.63 | 0.02 | 27 |
| **28** | 28.90 | 0.48 | 0.02 | 29 |
| **29** | 29.13 | 0.86 | 0.03 | 29 |
| **30** | 29.70 | 0.75 | 0.03 | 30 |

**Table S5. Results of application of our increment counting algorithm to sine wave patterns of known increment counts.** Transects have been split into seven sections, with increasing amounts of noise applied (indicated by signal-to-noise ratio values; SNR). *CV*: coefficient of variation.

**4.** **Accounting for increments split between two neighbouring sections**

Splitting greyscale transects into several sections risks the chance of only partially capturing increments within one particular section (Fig. 2b-c). As only the ascending/descending limb of such features would be captured in two neighbouring sections, they may not be detected as a peak/trough in greyscale in either section using the first stage of the increment counting algorithm, which defines peaks or troughs with reference to the two respective troughs or peaks surrounding them. As it is known whether each section starts and ends with either a detected peak or a trough, the greyscale value of the final pixel of each section does indicate whether the section ends part-way through a further peak or trough. Therefore, if the last recorded feature of a section using ‘Findpeaks’ is a trough, then a final greyscale value above the sections upper standard deviation may be defined as the ascending limb of a peak, and vice versa.

This hypothesis is tested by comparing the greyscale value of the first and last recorded pixel in the section in question, the greyscale of the last pixel in the preceding section, the first recorded pixel in the proceeding section, and the upper/lower standard deviations of each section. If a) both the first recorded feature of this section and the last recorded feature of the preceding section are troughs; but b) both the first value of the section and last value of the preceding section are above both of their upper standard deviations; and c) an additional 0.5 has not been added to the increment count of the preceding section; then d) an additional 0.5 is added to the increment count of the section. Else, if a) both the first recorded feature of the section and the last recorded feature of the preceding sections are peaks; but b) both the first value of the section and last value of the preceding section are below both of their lower standard deviations; and c) an additional 0.5 has not been added to the increment count of the preceding section; than d) an additional 0.5 is added to the increment count of the section (Fig. 2b-d).

Also, if a) both the last recorded feature of this section and the first recorded feature of the proceeding section are troughs; but b) the last pixel of this section and the first value of the proceeding section are above the upper standard deviations of both sections; then c) add 0.5 to the increment count of this section. Else if a) both the last recorded feature of this section and the first recorded feature of the proceeding section are peaks; but b) the last pixel of this section and the first value of the proceeding section are below the lower standard deviations of both sections; then c) add 0.5 to the increment count of this section (Fig. 2b-d).

**5. Thin-section imaging**

Thin sections were studied using a Nikon Eclipse LV100 microscope equipped with a Nikon DS-Fi2 digital camera and using 20×, 50× and 100× objectives under polarised and non-polarised light. The Nikon DS-Fi2 camera offers a field of view of 2560×1920 pixels for each objective.

**6.** **Testing the effects of variability in straightening on increment counting**

The only part of the proposed increment counting algorithm that requires human vision is the guidance of the straightening process in ImageJ/Fiji (Schneider et al., 2012). This process requires the user to plot a segmented midline through the centre of the cementum in CT slices using the ‘Segmented line tool’. The user then inputs the number of pixels on either side of this midline that must be included in the ImageJ/Fiji ‘Straighten’ tool to include all of the cementum. The variability in plotting this midline and determining the pixel number and extent of the cementum results in differences in the extent and morphology of straightened and isolated cementum images on the resulting straightened images, and this may also affect the increment count estimates produced by our increment counting algorithm.

To investigate the effect of this variation on resulting increment counts, due to differences in plotting a midline through the centre of the cementum increment in straightened images of the same CT slice, one random CT slice was selected from the datasets of the k91, k16 and l10 specimens. These were then straightened and filtered five times per slice (five attempts) following the workflow outlined in Section ‘2.4 Cementum increment counting algorithm’ of the main document of this publication, and increments were counted using the increment counting algorithm presented here.

For each slice, mean estimated increment counts vary by a maximum of 0.29 for k91, 0.73 for k16, and 0.33 for l10. Maximum standard deviations are 0.82 for k91, 1.14 for k16, and 1.5 for l10 (Table S6). This suggests that the variation in morphology resulting from semi-automatically straightening PPCI SR CT slices does not have a significant effect on the resultant cementum increment counts.

| **Specimen** | **Straighten attempt** | **Mean estimated count** | **Minimum estimated count** | **Maximum estimated count** | **Standard deviation** |
| --- | --- | --- | --- | --- | --- |
| **k91** | 1 | 9.72 | 8.5 | 11.5 | 0.74 |
|  | 2 | 9.61 | 8.5 | 12 | 0.77 |
|  | 3 | 9.67 | 8.5 | 12 | 0.78 |
|  | 4 | 9.90 | 9 | 12 | 0.76 |
|  | 5 | 9.86 | 9 | 13 | 0.82 |
| **k16** | 1 | 9.06 | 7.5 | 13 | 1.14 |
|  | 2 | 9.64 | 8 | 12.5 | 1.13 |
|  | 3 | 9.56 | 8 | 13 | 1.14 |
|  | 4 | 9.79 | 8 | 11 | 0.84 |
|  | 5 | 9.77 | 7.5 | 13 | 0.86 |
| **l10** | 1 | 9.31 | 7 | 13.5 | 1.50 |
|  | 2 | 9.17 | 7 | 13.5 | 1.24 |
|  | 3 | 9.11 | 7 | 14.5 | 1.34 |
|  | 4 | 9.06 | 7 | 12.5 | 1.32 |
|  | 5 | 9.39 | 7 | 14.5 | 1.37 |

**Table S6. Variability of automated increment counting results due to manual midline plotting through the cementum centre of three specimens.** Results shown for five alternative attempts to straighten and filter the same region in each specimen.

**Supplementary references**

- Freeman WT, Adelson EH. The Design and Use of Steerable Filters. IEEE Transactions on Pattern Analysis and Machine Intelligence. 1991;13(9):891–906.
- Hammer Ø, Harper DA, Ryan PD. PAST: Paleontological statistics software package for education and data analysis. Palaeontol. Electron. 2001;4(1):9.
- Schneider CA, Rasband WS, Eliceiri KW. NIH Image to ImageJ: 25 years of image analysis. Nat. Methods. 2012;9(7):671.

### Figure S1.

### Figure S1. Assessment of the effects of directional filtering on cementum images. (a) Original straightened (8-bit) image of the cementum of the *Macaca mulatta* individual l14. (b) 8-bit image in (a) after Gaussian filtration oriented along the *y*-axis and a first directional derivative, consisting of only greyscale values of 255 (light increments) and zero (dark increments). (c) Filtered image created by addition of (b) to the original image, after division of greyscale values in (b) by two (‘Filter 2’). (d) Filtered image created by addition of (b) to the original image, after division of greyscale values in (b) by eight (‘Filter 8’). (e) Greyscale values along transect marked with dashed red line in (b). Coloured lines indicate the greyscale values created by dividing values in (b) by integers between two and nine. (f) Comparison between greyscale distributions of Filter 2 (black), Filter 8 (blue) and the original image (red). The red box highlights the distinction between primary peaks and secondary peaks in greyscale used when assessing the potential loss of detail due to filtering. (g) Comparison of modulation transfer function (MTF) of cementum increments between filters. Red boxes indicate filters with significant differences in modulation compared to the original image (green box), whereas blue boxes indicate filters without significant differences to the original (following ANOVA; see Table S1). All scale bars are 75 µm.

### Figure S2.

###
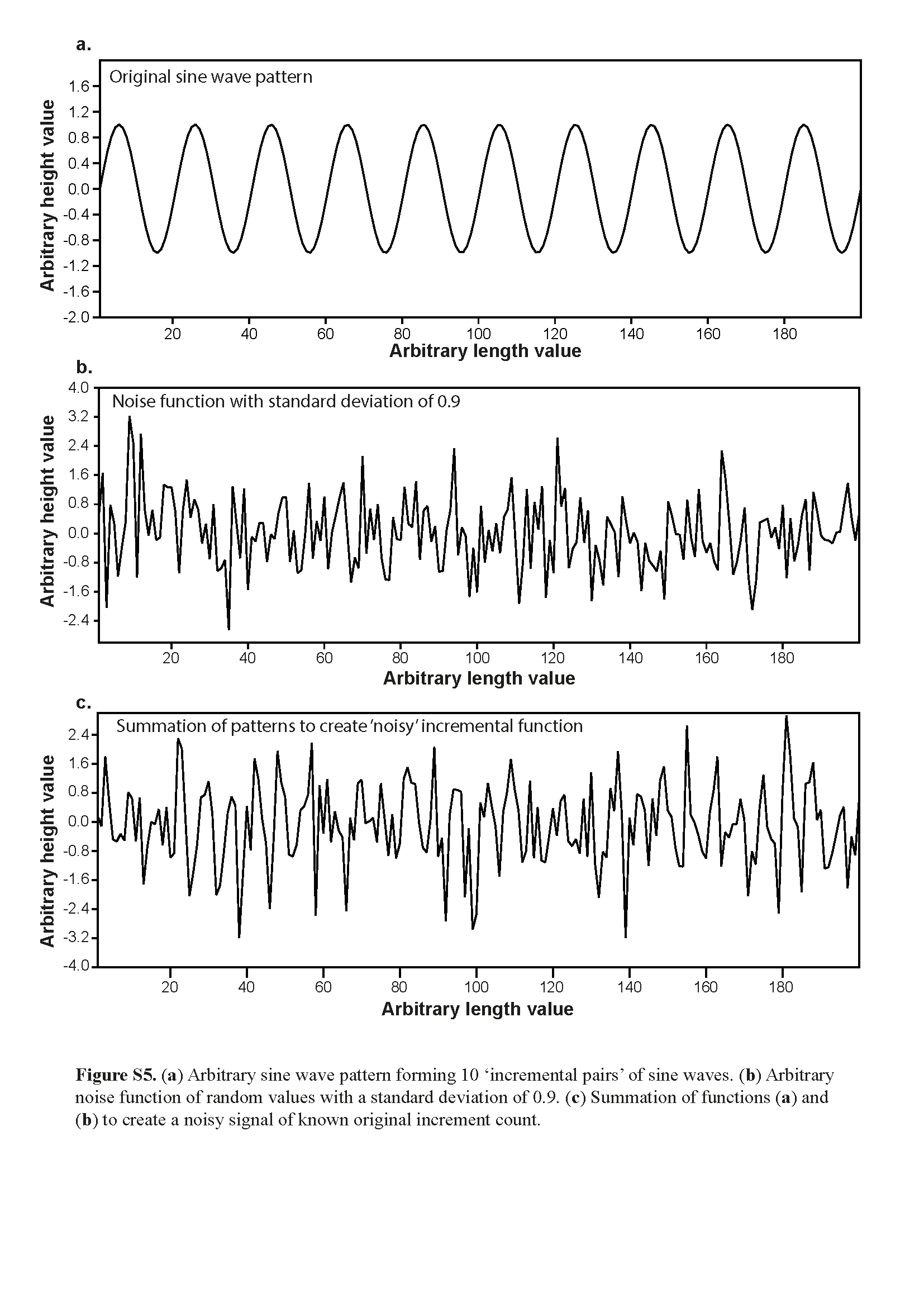


**Figure S2. Sine wave patterns for robustness testing of increment counting.** (**a**) Arbitrary sine waves pattern forming 10 ‘incremental pairs’ of sine waves. (**b**) Arbitrary noise function of random values with a standard deviation of 0.9. (**c**) Summation of functions (**a**) and (**b**) to create a noisy signal of 0.1 SNR level, but of known original increment count.

**Figure S3**

**
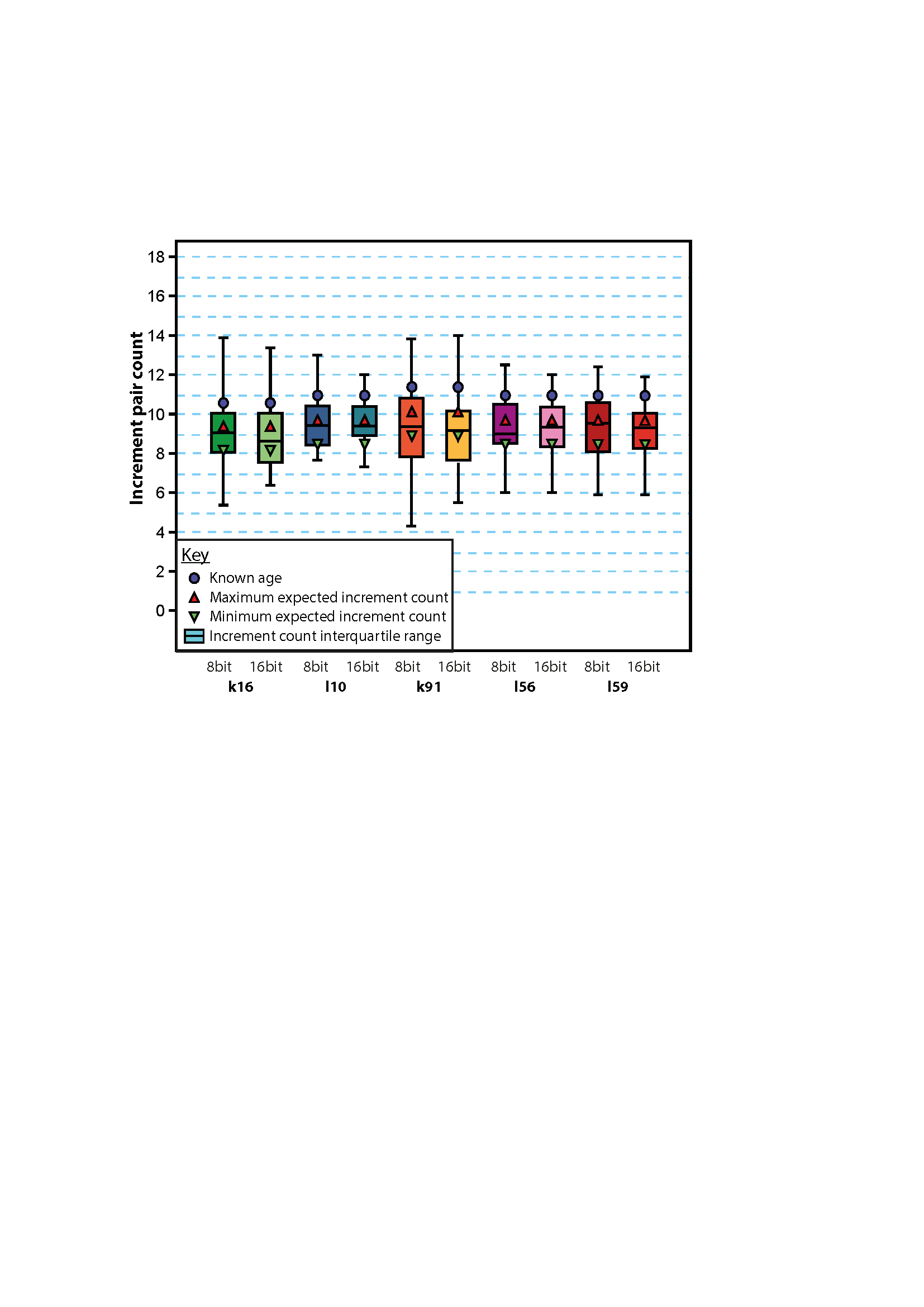
**

**Figure S3. Comparison of estimated increment counts between 8-bit and 16-bit filtered datasets.** Datasets have been generated for five specimens.

**Figure S4.**

**
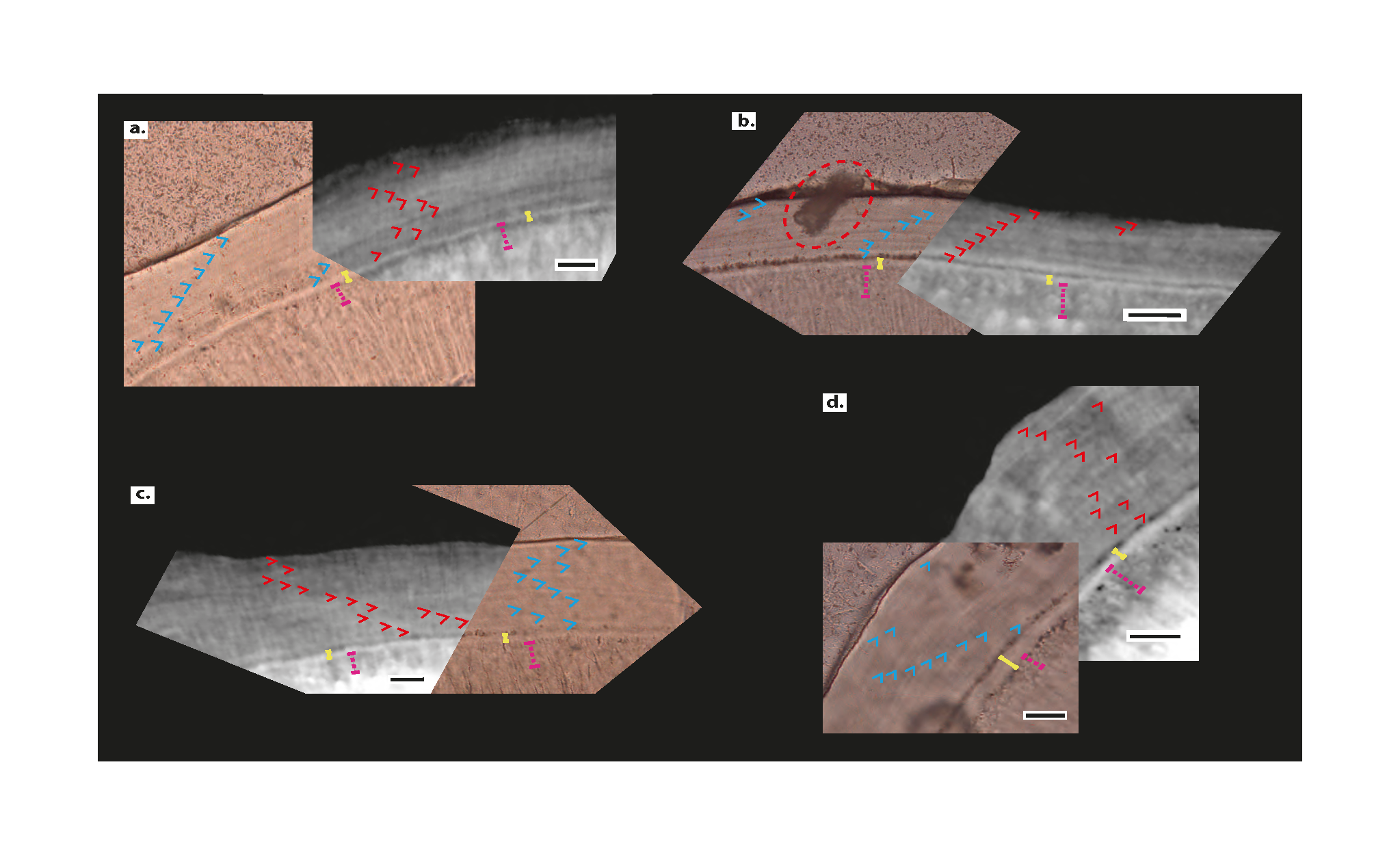
**

**Figure S4. Direct overlap of histological and PPCI data of cementum regions shown in Figure 5.** (**a**) Overlap of corresponding regions of cementum data from the k49 specimen. (**b**) Overlap of corresponding regions of cementum data from the k16 specimen. (**c**) Overlap of corresponding regions of cementum data from the l59 specimen. (**d**) Overlap of corresponding regions of cementum data from the l56 specimen. (**a-d**) Red arrows highlight cementum increments in PPCI SR CT data and blue arrow highlight increments in histological data. Yellow whiskers highlight the granular layer of Tomes and pink dashed whiskers highlight the hyaline layer of Hopewell-Smith. Red dashed circles highlight surface damage created during thin-section processing. Black scale bars represent 30 µm.

**Figure S5.**


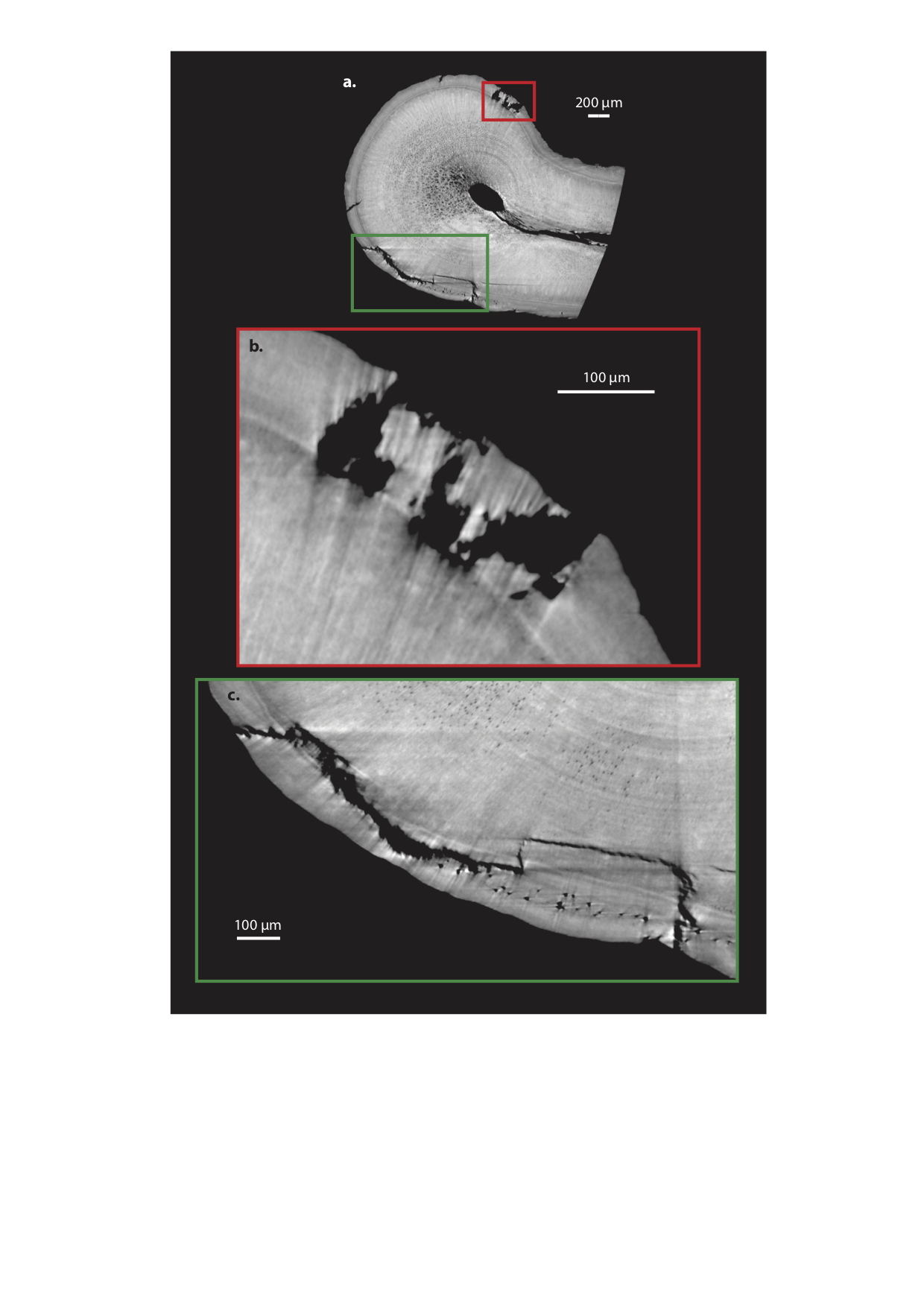


**Figure S5. Examples of microcracks and damage of cementum encountered during synchrotron radiation-based computed tomographic (SR CT) imaging of suboptimally prepared material.** (**a**) Overview of a single PPCI SR CT slice of the left m1 tooth root of the l14 individual, showing two key areas of damage highlighted by red and green boxes. (**b**) Detail of the red box in (**a**), displaying burst cellular voids. (**c**) Detail of the green box in (**a**), displaying the formation of a circumferential crack through the cementum, and expanding cell voids indicated by characteristic movement artifacts along their boundaries (streaks).
