# Supplementary material for: A robust, semi-automated approach for counting cementum increments imaged with synchrotron X-ray computed tomography
Source: PLoS One. 2021 Nov 4;16(11):e0249743. doi: 10.1371/journal.pone.0249743 (PMC8568193; doi:10.1371/journal.pone.0249743)
Supplement: Attachment — Submitted filename: PlosOne_Newham_et_al_Review.pdf [file pone.0249743.s003.pdf]

Thank you for asking me to review the work by Newham and colleagues.

This piece of research is most valuable and represent a considerable step forward in the field of cementochronology, with potential implications for studying hominin fossils, yet with some limitations that I will highlight later in this review.

I would like to congratulate the authors for the work and encourage them to consider the comments and suggestions I made, after what I will fully support the publication of this work in PlosOne.

I have provided a detailed annotation of the main manuscript (attached pdf). Please find below some more general comments:

Abstract

- see my comment, the claim are currently not supported by any reported statistical results. Please refer to your correlation tests.

- After reading in depth the paper and SOM, I understand why there is no highlight about the non-destructive nature of synchrotron µCT imaging, this is later explained by the destructive protocol used. Yet, for future directions, it would be good to stress that non-destructive imaging is possible.

Introduction

- errors in biblio

- lack of precision regarding location of AEFC and CIFC.

- improve clarify regarding the use of the word "cementum": complex tissue with sub-types, indicate here clearly what is being studied.

Methods + Results

- the protocol used here is highly destructive! The crown was sectioned, and the roots separated. The end of the introduction involves a kind of blunt criticism about the pilot study of Le cabec et al 2019, yet, they did not cut the teeth, and use higher energy to avoid damaging the DNA!

- Whether the teeth used here are deciduous or permanent is not clear until the discussion. This should be stated at the beginning of the "Materials".

- There are no explanation about how the thin sections were made? Any polishing involved? Slice thickness?

- SR µCT acquisitions: Why choosing 1501 projections whereas SNR seems best around 3000-4000 projections?

- There is a lack of explanation regarding what has been scanned: where is the FOV/ROI on the root? What size? Which root? Where along the root length? Was the ROI chosen in a standardized manner for all individuals? Explain the criteria. A figure would be helpful.

- Figure 2: I think there is a mismatch between the x values in Fig 2b and Fig 2c? (if you track the peaks, and corresponding x-values).

- Cite more often your supplementary information.

- 16 or 8 bit data? This needs to be clarified and tested as it may have an impact on the results.

- Figure 3: on the plot, there are too many curves compared to the figure legend? Also rather indicate the resolution / pixel size and not the sample-detector distances, as this is less intuitive for most non-specialist readers.

- How do the method deal with second-order increments if any? In the SOM I see they are ignored, as well as on the figure showing the I and II in light blue. But is it so frequent? I might be good to show a picture, because those structures do exist. They have been identified also by SXRF.

- how were the 30 slices chosen within the stack? Or was the ROI chosen to involve 30 slices only? This is not clear.

- any use of thick slices (average of multiple slices to enhance visibility)? Or only native slices?

Discussion

L553-560: + Fig. S3: the scans generated micro-cracks: this is of concern!!! Maybe the specimen was still fresh and not totally dry? this could have induced the liberation of free radicals, especially with the low energy used.

This claim is actually going to be harmful to the synchrotron community, and a major concern! Why? Because ***non-specialists are going to be comforted in the <u>general belief</u> that synchrotron imaging damages specimens***. So it should be made clear here why and how these micro-cracks occurred, and *not let readers believe this will happen every time*!

Supplementary Material

Equations 2 and 3: is the second 0 the symbol for degree (0°)? then it should be superscript to 0 or 90, here it seems to have normal size and it thus seems confusing. Or L42, explain this is for 0°, this would help non-specialists to understand the notation.

Fig. S2 and others: individual plots should at least have 1 or 2 key words as title otherwise it is impossible what they represent without reading the full caption.

L66: so why not running this on the 16 bit data? It needs to be tested if running it on 8 bit induces a loss of contrast/details that would not occur when using 16 bit data.

L78-79: I am not sure to understand how you choose by which integer you divide the 1st directional derivative image.

L104: is not this circular reasoning? the PPC SR data without any processing, thus including background noise? this needs to be clarified.

L161: why "tribological" in the title??? and L187, L286: I don't understand the use of this word "tribological"? To me, this has to do when 2 surfaces are in contact and involve movement (e.g., tooth occlusal surfaces and food items). Please either define, rephrase (explain) or delete.

Table S5: typo.
