# Supplementary material for: A robust, semi-automated approach for counting cementum increments imaged with synchrotron X-ray computed tomography
Source: PLoS One. 2021 Nov 4;16(11):e0249743. doi: 10.1371/journal.pone.0249743 (PMC8568193; doi:10.1371/journal.pone.0249743)
Supplement: Attachment — Submitted filename: PONE-D-21-09124_R1_reviewer.pdf [file pone.0249743.s005.pdf]

PLOS ONE

A robust, semi-automated approach for counting cementum increments imaged with X-ray computed tomography

--Manuscript Draft--

**Manuscript Number:**	PONE-D-21-09124R1
**Article Type:**	Research Article
**Full Title:**	A robust, semi-automated approach for counting cementum increments imaged with X-ray computed tomography
**Short Title:**	A semi-automated approach for counting cementum increments in X-ray tomographic data
**Corresponding Author:**	Elis Newham, Ph.D.<br>Queen Mary University of London<br>London, London UNITED KINGDOM
**Keywords:**	Cementum; X-ray tomography; synchrotron; lifespan; imaging optimisation
**Abstract:**	Cementum, the tissue attaching mammal tooth roots to the periodontal ligament, grows appositionally throughout life, displaying a series of circum-annual incremental features. These have been studied for decades as a direct record of chronological lifespan. The majority of previous studies on cementum have used traditional thin-section histological methods to image and analyse increments. However, several caveats have been raised in terms of studying cementum increments in thin-sections. Firstly, the limited number of thin-sections and the two-dimensional perspective they impart provide an incomplete interpretation of cementum structure, and studies often struggle or fail to overcome complications in increment patterns that complicate or inhibit increment counting. Increments have been repeatedly shown to both split and coalesce, creating accessory increments that can bias increment counts. Secondly, identification and counting of cementum increments using human vision is subjective, and it has led to inaccurate readings in several experiments studying individuals of known age. Here, we have attempted to optimise a recently introduced imaging modality for cementum imaging; X-ray propagation-based phase-contrast imaging (PPCI). X-ray PPCI was performed for a sample of rhesus macaque (Macaca mulatta) lower first molars (n=10) from a laboratory population of known age. A new method for semi-automatic increment counting was then integrated into a purpose-built software package for studying cementum increments. Qualitative comparison with data from conventional cementochronology, based on histological examination of tissue sections, confirmed that X-ray PPCI reliably non-destructively records cementum increments (given the appropriate preparation of specimens prior to imaging). Validation of the increment counting algorithm suggests that it is robust and provides accurate estimates of increment counts. In summary, we show that our new increment counting method has the potential to overcome caveats of conventional cementochronology approaches, when used to analyse 3D images provided by X-ray PPCI.
**Order of Authors:**	Elis Newham, Ph.D.
	Pamela G Gill
	Kate Robson Brown
	Neil J Gostling
	Ian J Corfe
	Philipp Schneider
**Response to Reviewers:**	Response to the Academic Editor:<br>We thank the editor for their positive review of our article and offer for revision and resubmission. We concede that comparison with the results of similar studies on archaeological and fossil teeth suggest that the damage observed in our experiment was most likely due to suboptimal preparation of teeth prior to scanning. We have adjusted our discussion of this accordingly, to minimise any inference that synchrotron

Powered by Editorial Manager® and ProduXion Manager® from Aries Systems Corporation

imaging itself is necessarily destructive.
We have revised our citation format and reference list to both conform to that of PLoS ONE, and amend inconsistencies in the reference list. We have also revised our scale bar description and scaling in all figures. Finally, following the comments of the editor and reviewers, we have aimed to make the narrative more broad in relation to cementum biology and synchrotron radiation-based X-ray imaging.
We now include the statement “No permits were required for the described study, which complied with all relevant regulations” to conform to PLoS ONE’s reporting requirements.
We also now explicitly state what funding from Ginko investments was used for (Line 665: “Funding was also provided by Ginko Investments Ltd (Bristol, UK) for purchasing external hard drives and funding travel to the Swiss Light Source, and the Academy of Finland. And on line 673: ”The funders had no role in study design, data collection and analysis, decision to publish, or preparation of the manuscript.” We also amend our competing interest statement as: “The authors report no conflicts of interest. The private funding received from Ginko Investments Ltd does not alter our adherence to PLOS ONE policies on sharing data and materials.”

We hope that the changes and revisions to our manuscript are satisfactory.

Response to reviewer 1:
Reviewer #1: This is an important contribution to the literature on cementum. It defines the nature of an annual increment in acellular cementum and describes a semi-automated method for making objective quantitatively based counts of annual cementum increments. It is significant that these counts closely match those made with traditional transmitted light microscopy in the same specimens.
We thank the reviewer for their considered and positive review.
The study is based on animals that were raised under laboratory conditions (presumably caged for most of their lives). Since this suggests they were buffered from the external seasonal environment and since the presumption is also that their diet was of a consistent nature throughout their lives, the presence of annual increments in their cementum suggests this is an endogenous rhythm and not one that has been assumed by some to be entirely driven by seasonality and or shifts in dietary quality and/or toughness. Some reflection of this in the discussion might be useful for people to at least ponder the implications.
We agree that the above-mentioned factors point to the circum-annual periodicity of cementum incrementation following an endogenous, potentially hormonal rhythm. We have thus altered our discussion to include (Line 567): “This is of particular note given that the study sample originates from a captive population with minimal seasonal environmental and nutritional variation. It is understood that the cementum of captive mammals presents lower increment contrast than free-roaming populations (Klevezal, 1995), and so the identification and accurate counting of circum-annual increments of PPCI data in captive cementum may be another promising element of this technique. This also adds to the body of work finding circum-annual increments in modern humans (Naji et al., 2016 and references therein), suggesting that incrementation follows an endogenous (potentially hormone-driven; see Newham et al., 2020a) rhythm as opposed to primarily reflecting seasonal dietary differences (Liberman, 1993).”
The age range of the animals in this study is as expected much less than the age range in studies of adult modern human cementum. It seems the accuracy of counts of annual cementum increments decreases with increasing age. This may imply later cementum increments, formed say after twenty of thirty years (way beyond the ages of animals in this study), are of a different nature, perhaps more likely fused together or so much slower in formation rate that they can’t be resolved. It might be a good thing to offer some suggestions about the problems this study may not have been able to address as well as those it clearly does address so well and that are discussed. In this way other potential factors affecting, particularly large cementum increment counts in older humans, are less likely to be swept under the carpet and more likely to be addressed in other ways. I’m particularly happy to see Zander and Hurzeler (1958) making it back into the cementum literature. It is one of very few studies that contains data on rates of cementogenesis in different modern human tooth types over many years.
While several validation studies have found exceptional accuracy using

Powered by Editorial Manager® and ProduXion Manager® from Aries Systems Corporation

cementochronology to age humans living up to and above 90 years of age (Kagerer and Grupe, 2001; Witwer-Backhofen et al., 2004), others have indeed shown a decrease in accuracy with increasing age (Mani-Caplazi et al,, 2019; see Naji et al., 2016 for a review). It is known that the cementum of long living animals including humans shows an increasing increment density (i.e. numer of increments per unit length), with increment count increasing faster than increases in radial width of the cementum. This increase in increment density evidently makes subjective determination of increments more difficult, given a certain spatial resolution. Thus, it may well be that the optimum parameters found for our sample will be suboptimal for others. Several synchrotron beamlines can now readily achieve effective voxel sizes of around 0.3 µm, which may be preferable for distinguishing densely spaced increments in human cementum. As we state (now in more detail; see Line 537) in our discussion, we believe our results indicate that each experiment based on novel material to cementum SR CT imaging should begin with a parameter sweep to achieve the optimum data relative to the goals of the particular experiment, including the biological nature of the sample itself.

The materials and methods are very carefully described and left and right ‘m1s’ – better to say M1s because they are permanent teeth – were fixed for 10 days in paraformaldehyde. Can the authors present some evidence that there was no demineralisation from the surface inwards as a result of this that might have introduced (or altered) a gradient in the grey scale values e.g. in Figure 2?

We cannot conclusively state whether any demineralisation occurred due to fixation in PFA, and this may be one of the factors that led to microscale cracking witnessed during the experiment (Figure S5). Preparation of the sample and its effects on imaging are now discussed in the revised manuscript (Line 598). For future work, we intend on dehydrating all teeth through desiccation prior to scanning.

References:

•Condon K, Charles DK, Cheverud JM, Buikstra JE. Cementum annulation and age determination in Homo sapiens. II. Estimatres and accuracy. AM. J. Phys. Anthropol. 1986;71:321-330.
•Kagerer P, Grupe G. On the validity of individual age-at-death diagnosis by incremental line counts in human dental cementum. Technical considerations. Anthropol. Anz. 2001;59:331-342.
•Mani-Caplazi G, Hotz G, Wittwer-Backofen U, Vach W. Measuring incremental line width and appearance in the tooth cementum of recent and archaeological human teeth to identify irregularities: First insights using a standardized protocol. Int J Paleopathol. 2019;27:24-37.
•Naji S, Colard T, Blondiaux J, Bertrand B, d’Incau E, Bocquet-Appel JP. Cementochronology, to cut or not to cut? Int. J. Paleopathol. 2016;15:113-119.
•Wittwer-Backofen U, Gampe J, Vaupel JW. Tooth cementum annulation for age estimation: results from a large known-age validation study. Am. J. Phys. Anthropol. 2004;123:119-129.

Response to reviewer 2:

Thank you for asking me to review the work by Newham and colleagues.

This piece of research is most valuable and represent a considerable step forward in the field of cementochronology, with potential implications for studying hominin fossils, yet with some limitations that I will highlight later in this review.

I would like to congratulate the authors for the work and encourage them to consider the comments and suggestions I made, after what I will fully support the publication of this work in PlosOne.

I have provided a detailed annotation of the main manuscript (attached pdf). Please find below some more general comments:

We thank the reviewer for their extremely considered and thorough review. We appreciate their positive interpretation of the value of our study, and have taken on board their critiques. We have thus considerably revised the elements raised by the reviewer, including new supplementary study and modification of discussion points that were rightfully highlighted as easily misinterpreted. We have gone through the detailed comments in the reviewers attached commented PDF, and attempted to correct all points accordingly. Please see below for direct replies to comments:

Abstract
- see my comment, the claim are currently not supported by any reported statistical results. Please refer to your correlation tests.
We have now changed the claim to “qualitative comparison” and have created a new figure in the Supplement (Figure S3), where histological and tomographic data are combined to provide qualitative evidence for this statement.
- After reading in depth the paper and SOM, I understand why there is no highlight about the non-destructive nature of synchrotron µCT imaging, this is later explained by the destructive protocol used. Yet, for future directions, it would be good to stress that non-destructive imaging is possible.
We have added a note in the abstract that synchrotron CT imaging is in general non-destructive, given appropriate preparation of the sample prior to scanning.

Introduction
- errors in biblio
We have adjusted errors and the citation style accordingly.
- lack of precision regarding location of AEFC and CIFC.
We have added more detail on current knowledge of cementum tissue distribution (see line 79), although it should not be expected to be homogeneous (Naji et al., 2016).
- improve clarify regarding the use of the word "cementum": complex tissue with sub-types, indicate here clearly what is being studied.
We have clarified that we are specifically interested in imaging and analysing AEFC.

Methods + Results
- the protocol used here is highly destructive! The crown was sectioned, and the roots separated. The end of the introduction involves a kind of blunt criticism about the pilot study of Le cabec et al 2019, yet, they did not cut the teeth, and use higher energy to avoid damaging the DNA!
The preparation procedure is indeed more destructive than that of Le Cabec et al. (2019), because one of our principal aims was validating the optimum experimental settings to achieve optimal image quality for PPCI of cementum, regardless of specimen provenance or the broader scientific goals. This meant ensuring that samples were processed to be optimal in shape for PPCI, with minimal “excess” tissue interfering with the X-ray beam outside of the region of interest being scanned. We now implicitly discuss these concerns in the paragraph starting at line 191.
Of course, this procedure is unsuitable for many archaeological/fossil specimens, and different experimental settings are necessary for maximal possible image quality while minimising X-ray dose (if a DNA analysis is planned following PPCI) and accounting for interference by other roots etc. Briefly, our focus was to push the imaging technique for best possible image quality that could be achieved at TOMCAT beamline. We now discuss the other constraints that will effect ‘optimum’ experimental settings for a particular cementum study from Line 539.
We apologise for our seemingly blunt review of Le Cabec et al. (2019), as this was definitively not our intention. We value the work of Cabec and co-workers as one of the founding studies for the synchrotron study of cementum and have revised our wording accordingly (Line 151: “Due to the uniqueness of their archaeological sample, the canines studied in Le Cabec et al. [30] could not be thin-sectioned.”). We also believe that our comparison with the current gold standard of thin-section histology is a necessary prerequisite for understanding the accuracy of PPCI data and how they reflect genuine biological structures, and the effects of taphonomic and/or diagenetic processes on this structure.
- Whether the teeth used here are deciduous or permanent is not clear until the discussion. This should be stated at the beginning of the “Materials”.
The teeth used are all permanent teeth, and we apologise if we did not make that sufficiently clear throughout. We have now added reference to this more frequently and from the start of the “Materials” section.
- There are no explanation about how the thin sections were made? Any polishing involved? Slice thickness?
The full thin-section processing procedure is available in Newham et al. (2020). We have also added the slice thickness in our revised manuscript.
- SR µCT acquisitions: Why choosing 1501 projections whereas SNR seems best around 3000-4000 projections?
We used 1501 projections for the principal experiment in order to scan the entire

Powered by Editorial Manager® and ProduXion Manager® from Aries Systems Corporation

sample within the allotted experimental time. This compromise is now discussed in terms of general PPCI study of cementum in the revised discussion: (Line 551 “Further, while increasing the number of angular projections increases image quality, it also increases the total acquisition time of each CT scan. As each synchrotron experiment is awarded a restricted amount of beam time, optimal experimental setings have to be weighed up against scanning time to image a sufficient sample of specimens. Hence, given the time allocated for our experiment, we retained 1501 projections per CT scan, half of the optimal value around 3000-4000 in terms of SNR (Fig. 4), in order to image our complete sample within the allotted beamtime”.

- There is a lack of explanation regarding what has been scanned: where is the FOV/ROI on the root? What size? Which root? Where along the root length? Was the ROI chosen in a standardized manner for all individuals? Explain the criteria. A figure would be helpful.

We have now explained that scans were centered on the coronal third of the anterior root of each tooth analysed. Three regions were scanned, which were overlapping longitudinally and covering a volume of approx. 41.31.3 mm3. See Line 248 for this explanation.

- Figure 2: I think there is a mismatch between the x values in Fig 2b and Fig 2c? (if you track the peaks, and corresponding x-values).

Many thanks for highlighting this mistake, which is now corrected.

- Cite more often your supplementary information.

The supplementary information is now cited in seven more places than the original manuscript.

- 16 or 8 bit data? This needs to be clarified and tested as it may have an impact on the results.

The original CT reconstructions were saved as 16-bit files, but the output of the steerable Gaussian filter function was saved as 8-bit files, and the increment counting procedure was developed for 8-bit data.

- Figure 3: on the plot, there are too many curves compared to the figure legend? Also rather indicate the resolution
/ pixel size and not the sample-detector distances, as this is less intuitive for most non-specialist readers.

The plot and key represent the different sample-to-detector distances examined, as shown in Figure 5.e. We use the actual distance between the sample and the detector, rather than the effective propagation distance that would require further explanation in the manuscript. We believe that the actual distance is an effective concept for the unexperienced reader that requires minimal knowledge or explanation of how the two factors (physical distance and effective pixel size) interact, especially when the effects of this distance are shown in Figure 3. Finally, due to the quasi-parallel nature of the synchrotron X-ray beam, effective propagation distance is nearly identical to sample-to-detector distance (Zeller-Plumhoff et al., 2017)

- How do the method deal with second-order increments if any? In the SOM I see they are ignored, as well as on the figure showing the I and II in light blue. But is it so frequent? I might be good to show a picture, because those structures do exist. They have been identified also by SXRF.

The method discounts increments with image contrast that does not exceed the local standard deviation of the image grey scale (i.e. “piggy-back features” as shown in Figure 5). However, the method does not directly discriminate between “real” circum-annual increments and “secondary” sub-annual increments. This would require subjective definition between increment types in each slice examined, which in-turn relies on user experience and interpretation. Our goal is instead to be as objective as possible, and to minimise the reliance of cementochronology on user experience (see Line 380: “While this procedure does not necessarily target or discriminate “accessory” or secondary increments from “real” circum-annual increments, it remains an objective way of discerning “real” increments that does not rely on subjective interpretation by the user. This in-turn relies on user-experience, which is one of the primary elements of cementochronology we are here seeking to minimise.”.

We understand from previous studies (Newham et al. 2020a,b) that secondary increments commonly form due to intermittent splitting of circum-annual increments, which we have attempted to overcome by intensively sampling over each examined slice, and over 30 slices per scan.

- how were the 30 slices chosen within the stack? Or was the ROI chosen to involve 30 slices only? This is not clear.

We apologise that we did not make that clear enough. The 30 slices were chosen

within regions with highest image contrast for increments upon manual examination of each scan. For each region, slices were selected until 30 slices had been chosen (for each scan). We have made this clearer in our revised manuscript accordingly (line 419: “Slices were selected contiguously through these regions until a number of 30 slices had been reached”).
- any use of thick slices (average of multiple slices to enhance visibility)? Or only native slices?
Native slices were used.

Discussion
Lines 553-560: + Fig. S3: the scans generated micro-cracks: this is of concern!!! Maybe the specimen was still fresh and not totally dry? this could have induced the liberation of free radicals, especially with the low energy used.
This claim is actually going to be harmful to the synchrotron community, and a major concern! Why? Because non- specialists are going to be comforted in the general belief that synchrotron imaging damages specimens. So it should be made clear here why and how these micro-cracks occurred, and not let readers believe this will happen every time!
We apologise for this unanticipated interpretation of this discussion. By no means we intended to undermine confidence in synchrotron imaging, and have significantly modified our discussion to highlight that the preparation of the sample is the primary cause for microstructural damage, exemplified by comparison with data from archaeological (Le Cabec et al., 2019) and fossil (Newham et al., 2020a) data, Line 608: “It also became apparent during scanning that micrometre-scale cracks, which are not visible macroscopically, have formed within the cementum tissue (Supplementary Fig. S5), most probably due the interaction of the hard X-rays with residual water left within the teeth and/or related effects due to this interaction [47]. Although this damage could not be seen macroscopically, comparison with the lack of observations of any damage in archaeological [30] and fossil [32] teeth confirms that the preparation of “fresh” teeth was suboptimal for SR CT scanning, as residual water was retained. Further, it cannot be confirmed whether micro-cracks occurred solely during the scanning process, or started due to demineralisation during fixation in paraformaldehyde. This should be examined in future studies via microscopic examination of the cementum of fixed teeth. Moreover, further optimisation of the preparation procedure for fresh teeth is still needed, that ensures both minimal risk of biological contamination and minimal damage to the tissue during CT scanning.”

Supplementary material
Equations 2 and 3: is the second 0 the symbol for degree (0°)? then it should be superscript to 0 or 90, here it seems to have normal size and it thus seems confusing. Or L42, explain this is for 0°, this would help non-specialists to understand the notation.
This font has now been changed accordingly.
Fig. S2 and others: individual plots should at least have 1 or 2 key words as title otherwise it is impossible what they represent without reading the full caption.
Titles have been added to plots as suggested. Thank you very much for this useful comment.
L66: so why not running this on the 16 bit data? It needs to be tested if running it on 8 bit induces a loss of contrast/details that would not occur when using 16 bit data.
The output of the filtering function is first calculated at double precision (i.e. “float64” between 0 and 1) format, then converted to the chosen Tiff format (here as 8-bit data). As this conversion is proportional to the distribution of float64 values in the original filtered file, conversion to 16-bit data will experience the same (relative) greyscale distribution as that of an 8-bit conversion, although the absolute greyscale variation will be different. However, we have now run a test on a subsample of five straightened datasets converted to both 8-bit and 16-bit data and subjected to our increment counting algorithm. This test shows that both bit depths produce comparable accuracy of estimated increment counts when compared to expected counts for each specimen. ANOVA comparisons between 8-bit and 16-bit datasets for each specimen only suggest a significant difference in increment counts for one single specimen, and Levene’s test show no significant difference in the variation between 8-bit and 16-bit datasets for each specimen. We interpret these results as suggestive that, while a minority of specimens may produce different ranges of absolute increment estimate values using 8/16-bit data, they are still centred on the same increment count and 16-bit data does not provide significantly higher accuracy or precision for increment count

	estimates than 8-bit data.<br>L78-79: I am not sure to understand how you choose by which integer you divide the 1st directional derivative image.<br>The optimum integer was chosen as the integer that provided the strongest image contrast between circum-annual cementum increments, while preserving as much greyscale contrast as possible. This was based upon qualitative examination of data filtered using each integer value successively, and monitoring when particular elements of the data were lost. While this is a subjective process, it was necessary to determine that as much data of interest (circum-annual increments) was preserved as possible, while minimizing the presence of greyscale noise.<br>L104: is not this circular reasoning? the PPC SR data without any processing, thus including background noise? this needs to be clarified.<br>While filtering aims to reduce background noise (i.e. unwanted signal), over-filtering can also remove potentially important and useful biological contrast (i.e. wanted signal). Thus, comparison between filtered data and original data was important to monitor what information (wanted vs unwanted signal) was being lost when applying increasingly strong filters, and to establish a cut-off where too much “wanted” biological signal was lost.<br>L161: why "tribological" in the title??? and L187, L286: I don't understand the use of this word "tribological"? To me, this has to do when 2 surfaces are in contact and involve movement (e.g., tooth occlusal surfaces and food items).<br>We agree that this term is confusing and have deleted the term throughout the manuscript.<br>Table S5: typo.<br>Typo corrected. Thank you very much for the detailed review, which is greatly appreciated.<br><br>References:<br>•Naji S, Colard T, Blondiaux J, Bertrand B, d’Incau E, Bocquet-Appel JP. Cementochronology, to cut or not to cut? Int. J. Paleopathol. 2016;15:113-119.<br>•Newham E, Gill PG, Brewer P, Benton MJ, Fernandez V, Gostling N J, et al. Reptile-like physiology in Early Jurassic stem-mammals. Nat. Commun. 2020a;11(1):1-13.<br>•Newham E. Corfe IJ, Brown KR, Gostling NJ, Gill PG, Schneider P. Synchrotron radiation-based X-ray tomography reveals life history in primate cementum incrementation. J. R. Soc. Interface. 2020b;17(172):20200538.<br>•Zeller-Plumhoff B, Mead JL, Tan D, Roose T, Clough GF, Boardman RP, et al. Soft tissue 3D imaging in the lab through optimised propagation-based phase contrast computed tomography. Opt Express. 2017;25(26):33451-68.
**Additional Information:**	
**Question**	**Response**
**Financial Disclosure**<br><br>Enter a financial disclosure statement that describes the sources of funding for the work included in this submission. Review the submission guidelines for detailed requirements. View published research articles from *PLOS ONE* for specific examples.<br><br>This statement is required for submission and **will appear in the published article** if the submission is accepted. Please make sure it is accurate.	This study was part-funded by a Natural Environmental Research Council/Engineering and Physical Sciences Research Council doctoral candidateship (UK; grant number NE/R009783/1). Funding was also provided by Ginko Investments Ltd (Bristol, UK), and the Academy of Finland.

**Unfunded studies**<br>Enter: *The author(s) received no specific funding for this work.*<br><br>**Funded studies**<br>Enter a statement with the following details:<br>• Initials of the authors who received each award<br>• Grant numbers awarded to each author<br>• The full name of each funder<br>• URL of each funder website<br>• Did the sponsors or funders play any role in the study design, data collection and analysis, decision to publish, or preparation of the manuscript?<br>• **NO** - Include this sentence at the end of your statement: *The funders had no role in study design, data collection and analysis, decision to publish, or preparation of the manuscript.*<br>• **YES** - Specify the role(s) played.<br><br>* typeset	
**Competing Interests**<br><br>Use the instructions below to enter a competing interest statement for this submission. On behalf of all authors, disclose any competing interests that could be perceived to bias this work—acknowledging all financial support and any other relevant financial or non-financial competing interests.<br><br>This statement **will appear in the published article** if the submission is accepted. Please make sure it is accurate. View published research articles from *PLOS ONE* for specific examples.	The authors have declared that no competing interests exist.

**NO authors have competing interests**<br><br>Enter: *The authors have declared that no competing interests exist.*<br><br>**Authors with competing interests**<br><br>Enter competing interest details beginning with this statement:<br><br>*I have read the journal's policy and the authors of this manuscript have the following competing interests: [insert competing interests here]*<br><br>* typeset	
**Ethics Statement**<br><br>Enter an ethics statement for this submission. This statement is required if the study involved:<br><br>• Human participants<br>• Human specimens or tissue<br>• Vertebrate animals or cephalopods<br>• Vertebrate embryos or tissues<br>• Field research<br><br>Write "N/A" if the submission does not require an ethics statement.<br><br>General guidance is provided below. Consult the submission guidelines for detailed instructions. **Make sure that all information entered here is included in the Methods section of the manuscript.**	This study is focused on the analysis of the right lower first molars (m1) from a sample comprised of 10 female Rhesus macaques (Macaca mulatta), raised under laboratory conditions and bred for biomedical research at the Primate Breeding Facility of Public Health England, Salisbury (UK). The unit was managed by the Defence Science and Technology Laboratory (DSTL) prior to 1st October 2009. The Macaca mulatta colony has been closed and self-contained since 1982, from animals originally imported from India between 1978 and 1982. All animals used here were routinely monitored and checked for primate-borne diseases of risk to humans (included hepatitis B, herpes B, and tuberculosis), and were humanely killed using an overdose of pentobarbital, under Home Office establishment licence 70-1707, due to being unfit for breeding or whole-animal scientific procedural use. No animal was killed for the specific purpose of this experiment. Once the animals were killed, their lower jaws were mechanically dislocated and removed by Public Health England.<br>The studied sample was classed as Category B biological waste by the UK government. As no animal was sacrificed or harmed for the purpose of this study, no animal research ethics committee approval was needed. The study complied with all relevant regulations under the UK Animals (Scientific Procedures) Act 1986 (ASPA).

Powered by Editorial Manager® and ProduXion Manager® from Aries Systems Corporation

**Format for specific study types**<br><br>**Human Subject Research (involving human participants and/or tissue)**<br>• Give the name of the institutional review board or ethics committee that approved the study<br>• Include the approval number and/or a statement indicating approval of this research<br>• Indicate the form of consent obtained (written/oral) or the reason that consent was not obtained (e.g. the data were analyzed anonymously)<br><br>**Animal Research (involving vertebrate animals, embryos or tissues)**<br>• Provide the name of the Institutional Animal Care and Use Committee (IACUC) or other relevant ethics board that reviewed the study protocol, and indicate whether they approved this research or granted a formal waiver of ethical approval<br>• Include an approval number if one was obtained<br>• If the study involved *non-human primates*, add *additional details* about animal welfare and steps taken to ameliorate suffering<br>• If anesthesia, euthanasia, or any kind of animal sacrifice is part of the study, include briefly which substances and/or methods were applied<br><br>**Field Research**<br><br>Include the following details if this study involves the collection of plant, animal, or other materials from a natural setting:<br>• Field permit number<br>• Name of the institution or relevant body that granted permission	
**Data Availability**<br><br>Authors are required to make all data underlying the findings described fully available, without restriction, and from the time of publication. PLOS allows rare exceptions to address legal and ethical concerns. See the PLOS Data Policy and FAQ for detailed information.	Yes - all data are fully available without restriction

Powered by Editorial Manager® and ProduXion Manager® from Aries Systems Corporation

A Data Availability Statement describing where the data can be found is required at submission. Your answers to this question constitute the Data Availability Statement and **will be published in the article**, if accepted.<br><br>**Important:** Stating 'data available on request from the author' is not sufficient. If your data are only available upon request, select 'No' for the first question and explain your exceptional situation in the text box.<br><br>Do the authors confirm that all data underlying the findings described in their manuscript are fully available without restriction?	
**Describe where the data may be found in full sentences. If you are copying our sample text, replace any instances of XXX with the appropriate details.**<br><br>• If the data are **held or will be held in a public repository**, include URLs, accession numbers or DOIs. If this information will only be available after acceptance, indicate this by ticking the box below. For example: *All XXX files are available from the XXX database (accession number(s) XXX, XXX.*).<br>• If the data are all contained **within the manuscript and/or Supporting Information files**, enter the following: *All relevant data are within the manuscript and its Supporting Information files.*<br>• If neither of these applies but you are able to provide **details of access elsewhere**, with or without limitations, please do so. For example:<br><br>*Data cannot be shared publicly because of [XXX]. Data are available from the XXX Institutional Data Access / Ethics Committee (contact via XXX) for researchers who meet the criteria for access to confidential data.*<br><br>*The data underlying the results presented in the study are available from (include the name of the third party*	All data supporting this study will be made openly available from the University of Southampton repository ("https://doi.org/10.5258/SOTON/").

and contact information or URL).<br>• This text is appropriate if the data are owned by a third party and authors do not have permission to share the data.<br><br>* typeset	
Additional data availability information:	Tick here if the URLs/accession numbers/DOIs will be available only after acceptance of the manuscript for publication so that we can ensure their inclusion before publication.

Powered by Editorial Manager® and ProduXion Manager® from Aries Systems Corporation

Manuscript

Click here to access/download;Manuscript;Newham et al. 2021 PloS one revised MS 28.07.docx

A robust, semi-automated approach for counting cementum increments imaged with X-ray computed tomography

Elis Newham[1,2*], Pamela G. Gill.[3,4], Kate Robson Brown[5,6], Neil J. Gostling[7], Ian J. Corfe[8,9], & Philipp Schneider[1,10*].

* Corresponding author

1. Bioengineering Science Research Group, Faculty of Engineering and Physical Sciences, University of Southampton. University Road, Southampton, SO17 1BJ, United Kingdom.
2. School of Engineering and Materials Science, Queen Mary University of London, London, E1 4NS, United Kingdom.
3. School of Earth Sciences, University of Bristol. Wills Memorial Building, Queens Road, Bristol, BS8 1RJ, United Kingdom.
4. Department of Earth Sciences, Natural History Museum. Cromwell Road, London, SW7 5BD, United Kingdom.
5. Department of Mechanical Engineering, Queen's Building, University of Bristol, Bristol, UK.
6. Department of Anthropology and Archaeology, University of Bristol. 43 Woodland Road, Bristol, BS8 1UU, United Kingdom.
7. School of Biological Sciences, Faculty of Environmental and Life Sciences, University of Southampton, Southampton, UK.
8. Developmental Biology Program, Institute of Biotechnology, University of Helsinki. Viikinkaari 5D, University of Helsinki, Helsinki, Finland.
9. Geological Survey of Finland, Espoo, Finland.

10. High-Performance Vision Systems, Center for Vision, Automation & Control, AIT Austrian Institute of Technology, Vienna, Austria.

Abstract

Cementum, the tissue attaching mammal tooth roots to the periodontal ligament, grows appositionally throughout life, displaying a series of circum-annual incremental features. These have been studied for decades as a direct record of chronological lifespan. The majority of previous studies on cementum have used traditional thin-section histological methods to image and analyse increments. However, several caveats have been raised in terms of studying cementum increments in thin-sections. Firstly, the limited number of thin-sections and the two-dimensional perspective they impart provide an incomplete interpretation of cementum structure, and studies often struggle or fail to overcome complications in increment patterns that complicate or inhibit increment counting. Increments have been repeatedly shown to both split and coalesce, creating accessory increments that can bias increment counts. Secondly, identification and counting of cementum increments using human vision is subjective, and it has led to inaccurate readings in several experiments studying individuals of known age. Here, we have attempted to optimise a recently introduced imaging modality for cementum imaging; X-ray propagation-based phase-contrast imaging (PPCI). X-ray PPCI was performed for a sample of rhesus macaque (*Macaca mulatta*) lower first molars (n=10) from a laboratory population of known age. A new method for semi-automatic increment counting was then integrated into a purpose-built software package for studying cementum increments. Qualitative comparison with data from conventional cementochronology, based on histological examination of tissue sections, confirmed that X-ray PPCI reliably non-destructively records cementum increments (given the appropriate preparation of specimens prior to imaging). Validation of the increment counting algorithm suggests that it is robust and provides accurate estimates of increment counts. In summary, we show that our new

increment counting method has the potential to overcome caveats of conventional cementochronology approaches, when used to analyse 3D images provided by X-ray PPCI.

1. Introduction

Mammalian teeth comprise three principal mineralised tissues: enamel, dentine and cementum. Each tissue provides its own record of growth via incremental patterns observed using thin-section histology that track periodic changes in their growth rates. Development of enamel and dentine is largely truncated after the maturation of the tooth. However, cementum grows continuously throughout life and provides a more complete record of an individual's life history [1]. Incremental features found in cementum are well understood to have a circum-annual periodicity, with one thick translucent increment, and one thin opaque increment formed every year (when viewed in thin-section under transmitted light microscopy; Fig. 1). These contrasting opacities are hypothesised to be primarily due to seasonal changes in the mineralisation rates of the cementum hydroxyapatite matrix. Cementum is composed of bundles of collagen fibres emanating from both the cementum itself (intrinsic fibres), or from the periodontal ligament or PDL (extrinsic 'Sharpey's' fibres), wrapped within a hydroxyapatite matrix [2]. Unfavourable growth seasons promote a reduction in the deposition rate of this matrix, while the mineralisation rate is unaffected [3]. This produces ultra-mineralisation of thinner portions of cementum during these periods ([1,4]; though see [3] and [5] for mineral distribution mapping studies that suggest higher mineral densities in wider, not narrower, cementum increments).

The proportion of collagen fibres emanating from either the cementum or the PDL defines the two major cementum tissue types. Acellular extrinsic fibre cementum (AEFC) contains predominantly Sharpey's fibres from the PDL. While the distribution of either tissue is not homogeneous, there is a general consensus that AEFC is concentrated in the crownward/coronal third of the root, while CIFC is the predominant tissue of the apical third of the root [6]. This tissue has the most regular periodicity and provides the most consistent growth record in the cementum. Cellular intrinsic fibre cementum (CIFC) contains only fibres originating from the cementum itself. This tissue grows more sporadically than AEFC with a less precise periodicity and is known to nucleate within regions of the tooth that undergo anomalously high occlusal forcing. As such, AEFC is usually the recommended cementum tissue for cementochronological studies and the tissue focussed on for this study ([6] and references within).

Several studies have questioned the accuracy, precision, and reliability of current methods of imaging and analysing cementum increments [7,8]. CIFC cementum is a dynamic, biomechanically responsive tissue and increments are known to both split and coalesce. These phenomena are also known to occure in AEFC cementum, which can undermine confidence in their counts as an estimate of age at death. The overwhelming majority of previous studies have relied on thin-sectioning samples to image increments using light microscopy [6]. This allows increments to be viewed at high spatial resolutions, and offers a range of optical [9] and digital image processing [10,11] methods that filter and highlight increments to varying degrees. However, the destructive nature of thin-sectioning and the restrictive two-dimensional (2D) perspective offered by histological sections limit the understanding of the complex nature of cementum and its increments [7]. There is a wide range in the

reported accuracy (the proximity of estimated increment counts to known/true chronological age in years) and precision (repeatability and reproducibility between researchers) of increment counts [7,12,13], despite generally high reported accuracy and precision across multiple studies [6].

Computer vision and image processing have been explored to aid human counting of cementum increments, and to overcome the need for human counting itself [14,15]. Peaks and troughs in cementum opacity or greyscale 'luminance' can be extracted along radial transects through the cementum from digital micrographs of thin-sections using image processing software and studied by using 'Digital Cementum Luminance Analysis' (DCLA) [11] (Fig. 2a-b). Light increments are represented by distinct peaks in luminance values, and dark increments by distinct troughs in luminance values (Fig. 2b). These patterns are interpreted by either manually counting peaks and/or troughs, or through peak/trough detection algorithms (e.g. 'Find peaks' in ImageJ/Fiji; [16]). This abstraction of increments to peaks and troughs offers a less subjective method for manually counting increments, compared to directly reading thin-section images (Fig. 2b). Further, the use of numerical greyscale values to distinguish neighbouring light and dark increments allows quantitative thresholds to be defined for distinguishing increments. These thresholds represent a specific greyscale value from which peaks/troughs representing light/dark increments must differ from either the last trough or peak (respectively), or from the mean value of greyscale for the transect under study, in order to be identified as a 'genuine' increment.

However, DCLA methods have so far relied upon *a priori* assumptions regarding the contrast in greyscale values caused by incrementation versus those caused by other sources such as image noise. The chosen threshold value for

distinguishing increments in each DCLA method is specific to the image technique and hence image data in the original study, and so may not be robustly applied to data from other imaging modalities or techniques, or to taxa that fail to meet the specified threshold specified in the original study. Thus, the next stage in DCLA development should focus on developing a more flexible strategy for distinguishing cementum increments based on relative instead of absolute greyscale distribution criteria.

3D imaging, such as high-resolution computed tomography (CT) using X-ray propagation-based phase-contrast imaging (PPCI) at synchrotron radiation (SR) sources, may overcome the limitations posed by counting cementum increments in histological thin-sections. SR CT has revolutionised the study of other hard tissue microstructures such as vascular and cellular networks in bone [17:22] or growth increments in enamel [22-27]. SR CT imaging of such tissues provides a 3D context to the study of internal structures at sub-micrometre levels, and, with high signal-to-noise ratio (SNR) and high contrast-to-noise ratios (CNRs), provides high levels of image quality. PPCI through SR CT has recently allowed increments to be followed through the cementum of the teeth of archaeological humans [28-30] and macaque monkeys and early mammal fossils [31,32], overcoming the limitations of 2D thin-section-based imaging. The study of Le Cabec et al. [30] investigated a sample of human teeth from an archaeological population of known age at death, and reported high precision between counts performed by different observers, and for repeated counts performed by the same observer. However, although a strong correlation was found between age estimated by increment counts and known age, the accuracy (proximity of estimated age to known age) of estimates fell from 2.5 years in individuals 20-29 years old to 28 years in individuals 60-89 years old (with counts consistently underestimating true age). Due to the uniqueness of their archaeological

sample, the canines studied in Le Cabec et al. [30] could not be thin-sectioned. On this account, it could not be determined whether the nature of the source of this inaccuracy was biological, diagenetic (chemical changes to the cementum changing and overprinting original increments), or technical (insufficient image contrast between increments due to similar material properties in terms of X-ray interactions and/or due to unsuitable imaging and/or CT reconstruction settings). Further optimisation of cementum analysis using SR CT must be accompanied by comparison with histological data of the same regions of interest (ROIs), in order to relate image quality of SR CT data to the current "gold standard" for cementum imaging.

Here, we aim to optimise PPCI through SR CT for studying cementum increments in 3D, in comparison with the respective histological data in 2D. We will particularly assess the potential for overcoming the limitations identified in current approaches for counting cementum increments. As a second objective, we provide a validated, semi-automated and robust algorithm for user-independent counting of cementum increments.

2. Materials and Methods

2.1. Teeth samples

This study is focused on the analysis of the permanent right lower first molars (m1) from a sample of 10 female Rhesus macaques (*Macaca mulatta*), raised under laboratory conditions and bred for biomedical research at the Primate Breeding Facility of Public Health England, Salisbury (UK) (Table 1). All animals used here were routinely monitored and checked for primate-borne diseases of risk to humans (including hepatitis B, herpes B and tuberculosis) and were humanely killed using an overdose of pentobarbital, under Home Office establishment licence 70-1707, due to

being unfit for breeding or whole-animal scientific procedural use. No animal was killed for the specific purpose of this study. Once the animals were killed, their lower jaws were mechanically dislocated and removed by Public Health England. Lower jaws were then freeze-stored at -20 °C prior to further tissue preparation. The studied sample was classed as Category B biological waste by the UK government. As no animal was sacrificed or harmed for the purpose of this study, no animal research ethics committee approval was needed. No permits were required for the described study, which complied with all relevant regulations under the UK Animals (Scientific Procedures) Act 1986 (ASPA).

To prepare specimens, lower jaws were first mechanically cleaned of soft tissue using surgical tools (scalpel, scissors and tweezers). The coronoid and angular processes were then removed using a handsaw. Once prepared, specimens were bathed in tap water in a sealed plastic container, which was stored in a fume cupboard for three weeks (21 days). This procedure was adopted to rot away the periodontal ligament and alveolar soft tissue that could not be mechanically removed. After three weeks, teeth were sufficiently loose within the jaw to be easily removed using surgical pliers. The left and right permanent m1 teeth of all animals were fixed in 10% paraformaldehyde (PFA) solution for 10 days to minimise risk of infection.

Finally, to minimise the X-ray absorption by dense mineralised tissue that was not of interest, the roots were mechanically separated before imaging. The crowns of all teeth were removed using a Buehler IsoMet® Low Speed precision sectioning saw equipped with an Acuthin™ blade (Buehler Ltd, Lake Bluff, IL, USA). Using the same saw, the anterior and posterior roots of each m1 tooth were mechanically separated and mounted on 2 mm-thick carbon fibre rods (CR200600; Ripmax Ltd, Enfield, UK) cut to 1.5 cm length, using cyanoacrylate super glue. While this is a

destructive preparation technique (relative to Le Cabec et al. [30]), we determined it as necessary to provide the optimum sample geometry for SR CT imaging. Following PPCI experiments, transverse histological thin-sections of approximately 70 µm thickness were prepared, cut through the region of ROIs that were acquired by PPCI through SR CT, following the method outlined in Newham et al. [31].

Chronological age at death in years was known for each individual (Table 1). This data point, and the average age of eruption of lower permanent m1 teeth for captive populations of *Macaca mulatta* (approximately 12-18 months; [33]) provided an expected increment count for each individual (age at death minus average age at eruption). However, a potential variation of six months for m1 eruption necessitated the use of a minimum expected increment count, and a maximum expected increment count (one increment higher than the minimum expected count) (Table 1). All thin-sections and remaining whole tooth samples were reposited at the University of Southampton and are accessible upon request to EN and/or PS.

2.2. X-ray PPCI of cementum

PPCI for this study was performed during a three-day experiment at the TOMCAT beamline of the Swiss Light Source (SLS) (15-18 March 2016). The station at TOMCAT allows the user to control a series of key experimental settings, affecting the image quality of the resulting CT data [34,35]. The effects of these experimental settings must be systematically assessed in order to achieve optimal experimental conditions for the specific purpose and required image quality of a study.

In a preliminary experiment, the cementum tissue of specimen l56 was mounted using the convention described above, and imaged using X-ray PPCI through SR CT for a range of different experimental settings. Four key experimental settings (X-ray energy, exposure time, number of X-ray projections, and sample-to-

detector distance) were individually varied according to Table 2, while all other experimental settings were fixed at an X-ray energy of 20 keV, a voxel size of 0.66 µm and an exposure time of 150 ms for 1501 projections, at a sample-to-detector distance of 14.00 mm, which corresponds to a similar effective X-ray propagation distance of 13.99 mm [35] due to the parallel X-ray beam geometry at TOMCAT. The effects of changing experimental settings on image quality of cementum increments were characterised by the signal-to-noise ratio (SNR) and the contrast-to-noise ratio (CNR) as figure of merits. SNR quantifies the level of the image signal relative to the background noise. CNR is a useful measure for assessing image contrast between distinct structures, such as dark/light cementum increments.

Image quality measures for each experimental setting were calculated for 10 reconstructed µCT slices, representing the same regions of the tooth root of l56 in each scan (Fig. 3). SNR was calculated as the ratio between the mean greyscale value (representing density) for a 150-pixel × 150-pixel ROI of cementum ($\overline{g_c}$) and the standard deviation of a 150-pixel × 150-pixel sample of background (i.e. air) (σ_b) in each slice:

$$SNR = \frac{\overline{g_c}}{\sigma_b} \qquad (1)$$

CNR was calculated as the difference of the mean greyscale values of the same ROIs of cementum ($\overline{g_c}$) and air ($\overline{g_b}$), respectively, divided by the pooled standard deviation, following the Pythagorean Theorem of Statistics ($Var(X \pm Y) = Var(X) + Var(Y)$ or $\sigma^2(X \pm Y) = \sigma^2(X) + \sigma^2(Y)$ for independent random variables X and Y, with Var and σ denoting the variance and standard deviation, respectively):

$$CNR = \frac{\overline{g_c} - \overline{g_b}}{\left[(\sigma_c^2 + \sigma_b^2)/2\right]^{1/2}} \qquad (2)$$

where σ_c and σ_b represent the standard deviations of cementum and background, respectively. Mean values of SNR and CNR were calculated from the values for these 10 slices and compared between all experimental settings (Fig. 4 and Table 2).

Following this preliminary study, an X-ray energy of 20 keV and a sample-to-detector distance of 14 mm was chosen to scan three ROIs within the coronal third of the anterior root of each tooth, overlapping longitudinally and covering a volume of approximately 4X1.3X1.3 mm^3 (Table 1). This provided sufficient image contrast between cementum increments. For each scan, the exposure time was set to 150 ms, and 1501 projections were taken per scan. These settings provided sufficient image quality at scan times that allowed the entire sample of teeth to be imaged during our fixed-time experiment. The phase of each X-ray projection was retrieved through the Paganin single-distance non-iterative phase retrieval algorithm, [36], implemented in-house at TOMCAT. The values of the imaginary part of the refractive index δ=3.7 10^{-8} and the decrement of the real part of the refractive index β=1.7 10^{-10}, and hence the ratio $\delta/\beta = 218$, were fixed for all scans. Phase contrast is much more sensitive for imaging low absorbency materials with small differences in material properties, compared to traditional X-ray absorption [37]. The retrieved phase images were reconstructed using an in-house implementation of the Gridrec algorithm [38] at TOMCAT. The resulting CT reconstructions were saved as 16-bit tiff stacks.

2.3. Image processing: straightening and filtering

Image processing of the “raw” reconstruction data is often needed before digital image visualisation, segmentation and quantification. This can involve a wide range of image processing methods, the majority of which are based on the manipulation of 2D pixels and/or 3D voxels using mathematical operations [39]. Here, we applied two principal image processing methods to individual transverse CT slices: straightening

and isolation of cementum, and directional filtering of cementum increments. Straightening and isolation involved distinguishing the cementum from the dentine tissue that it surrounds in each slice, removing the dentine data and algorithmically minimising/”straightening” the remaining cementum data along its circumferential axis (Fig. 5). This then allowed directional filtering to enhance image contrast in a single direction between straightened cementum increments.

For circumferential structures such as cementum increments (when viewed in CT slices transversely through the tooth root), it is often difficult to apply standard image processing tools and analyses without distorting results, due to complexities in their patterns and boundaries. Hence, 2D straightening algorithms are often applied in order to further analyse the data. We chose to use the ‘Straighten’ tool of the open source ImageJ/Fiji image analysis software [16]. This tool applies cubic-spline interpolation across a segmented midline of the feature of interest, which is defined by the user. Straightening is then performed using a series of non-linear cubic splines for an arbitrary number of pixels on either side of the midline that can also be determined by the user. Here, we assigned this number on an individual basis for each dataset, based on the (radial) thickness of cementum being imaged in the first/bottom-most slice, to ensure that all cementum, but no dentine, was included in the processed image (Fig. 5.a-b). This workflow was then repeated in a semi-automated fashion for all CT slices of each dataset following Newham et al. [31], wherein the same midline coordinates and thickness of the segmentation was applied for as many slices as possible from the first slice of a dataset. Due to the change in shape of the root and non-perfect alignment of the sample axis and scanning axis, these midline coordinates had to be adjusted several times through each scan, as the sample changed in size and drifted through the volume. This required manual inspection of the amount of

cementum captured by the segmentation through the volume from the first slice, and adjusting the thickness and midline coordinates.

Following straightening, cementum datasets were further processed using directional filtering in order to enhance contrast between increments (see Supplement, section 1. ‘Image processing by directional filtering’) (Fig. 5.d). Filtering is commonly used to suppress the contribution of unwanted signals such as noise, while preserving and enhancing the targeted signal or image contributions for the analysis in question (Freeman and Adelson, 1991). We used a custom MATLAB (R2016a; The MathWorks, Inc., Natick, MA, USA) tool called ‘SteerGauss’ (version 1.0.0.0) developed and made freely available by Lanman [40], in order to employ directional Gaussian filtering of straightened cementum images following Freeman and Adelson [41]. For directional Gaussian filtering, a Gaussian function for a set of 2D (x,y) Cartesian coordinates can be prescribed for any arbitrary orientation using a directional derivative operator that interpolates between two ‘basic’ Gaussian functions, directed at 0° and 90°, respectively. Straightened increments follow similar longitudinal paths, so a steerable filter can be used to select a single orientation of all increments in an image (Fig. 5.c-d). For the current study, the use of a directional Gaussian filter oriented at 90° to the x-axis has been shown to substantially enhance image contrast between straightened cementum increments (see Supplement, section 1. ‘Image processing by directional filtering’) (Fig. 5).

2.4. Cementum increment counting algorithm

The cementum increment counting algorithm developed here was designed to count cementum increments in a user-independent and semi-automated fashion, further developing the rationale proposed by DCLA for distinguishing individual increments

by using a cut-off point that greyscale peaks/troughs must differ by, in order to be counted as a genuine increment. Our algorithm employs population statistics (mean and standard deviation of greyscale values) to count increments, based on the unique distribution of greyscale values within each individual CT slice (see Supplement, section 2 ‘Robustness testing for increment counting algorithm’). As in DCLA, this method makes use of the average greyscale values along 10 pixel-thick transects through cementum (Fig. 2a). Adapting methods used in tribological surface profiling [42,43] , individual transects are separated into five sections of equal length (Fig. 2b) (see Supplement, section 3 ‘Splitting of transects through the cementum’). PPCI SR CT datasets of cementum show an overarching reduction in greyscale (density) values from the cemento-dentine boundary to the outer-most cementum increment (Fig. 2a-b) [31,32]. Therefore, the use of the mean greyscale value and its standard deviation for the entire transect, as opposed to local values for individual sections of the transect, may preclude the counting of genuine increments towards the outermost increment (as greyscale peaks/troughs are below the mean value), and counting of increments towards the cemento-dentine boundary (as greyscale peaks/troughs are above the mean value). The mean and standard deviation of greyscale values in each section is then calculated (Fig. 2b-d), and light-dark increment pairs are distinguished as peak-trough systems in greyscale that depart from the mean value beyond the local standard deviation in each section (Fig. 2c).

Most importantly, this new method for increment counting can be operated in a semi-automated fashion, following an algorithm implemented in the MATLAB statistical environment (see Appendix for MATLAB script). In MATLAB, each individual straightened and filtered cementum image is investigated along a series of 1000 transects through the cementum chosen at random using a random number

generator (Fig. 2a). For each transect, the distance across the transect that the first pixel above zero appears is saved, which gives the radial length of sampled cementum along the transect. Any transect that is less than the lower standard deviation of the saved lengths is then deselected and resampled until all transects fulfil the lower standard deviation of the original sample. Each transect is divided into five sections of equal length (Fig. 2b), and a cubic spline ('Smoothing spline' function in MATLAB) is fitted to the greyscale pattern captured within each section, in order to minimise the influence of image noise on peak/trough patterns [44] (Fig. 2b). For these five smoothed datasets, their mean greyscale value (red lines in Figure 2) is calculated. An upper 'cut-off' value (green lines in Figure 2) is then determined for each section as its mean greyscale value plus half the standard deviation of its greyscale values, and lower 'cut-off' value (blue lines in Figure 2) as the mean greyscale value minus half of the standard deviation. Two new datasets are then created for each section, the first comprised of only greyscale values above the mean, and the other of values below the mean (Fig. 2c). The dataset comprising higher greyscale values thus consists solely of greyscale 'peaks' (local apex in greyscale values), while the dataset comprising lower greyscale values consists solely of greyscale 'troughs' (local nadir in greyscale values) (Fig. 2c). The 'Findpeaks' tool (part of the default 'Signal processing toolbox' in MATLAB) is then used to identify peaks and troughs in their respective datasets (following multiplication with -1 to convert troughs into peaks) and calculate their difference from the mean greyscale value of that section. This allows peaks and troughs that extend beyond the top and bottom cut-off values (respectively) for each section to be identified, providing the first stage of estimating increment and increment pair counts (Fig. 2b-d).

Following this first estimate of increment and increment pair counts, further steps are taken within the algorithm to ensure that ‘piggy-back’ features (secondary peaks/troughs along the ascending/descending limbs of genuine increment peaks and troughs, formed either due to accessory increments from increment splitting/coalescence, or image noise) do not affect increment counts (Fig. 2). No peaks are counted that immediately proceed from the last respective peak; so only one peak is counted for every trough (in Fig. 2c. peaks i and ii are not counted). Also, no peak/trough system along the transect for which each feature is separated by less than three pixels along the transect (or 1.98 µm), are counted, to ensure that grey scale variations on a small scale do not influence increment counts (Fig. 2c peaks i and ii). While this procedure does not necessarily target or discriminate “accessory” or secondary increments from “real” circum-annual increments, it remains an objective way of discerning “real” increments that does not rely on subjective interpretation by the user. This in-turn relies on user-experience, which is one of the primary elements of cementochronology we are here seeking to minimise.

A final measure is taken to account for increments that are only partly captured inside a neighbouring set of sections along one transect (Fig. 2b-c.). As only the ascending/descending limb of such features would be captured in each section, they may not be detected as a peak/trough in greyscale in either section using the first stage of the increment counting algorithm, which defines peaks or troughs with reference to the two troughs or peaks surrounding them respectively. A second step is therefore undertaken to distinguish, measure and count these features based on their greyscale values relative to the upper and lower standard deviations of each neighbouring section (described in Supplement Section 4 ‘Accounting for

increments split between two neighboring sections'). Once increment pair counts are estimated for the 1000 random transects, the mean and standard deviation are calculated, providing a final estimate of cementum increment pair count.

The robustness of the proposed algorithm was tested by applying it to a series of digital sine wave patterns of known increment number between five and 30. Random noise at different degrees was applied to these patterns in a controlled manner by increasing their standard deviation along the *y*-axis (Fig. 6). Noise was increased incrementally by SNR decrements of 0.1; starting from a SNR of 0.9 and ending at an SNR of 0.1. For each SNR level, increments were counted for 30 sine wave patterns for each count between five and 30. Increment estimates were considered as accurate if the mean estimated count equalled the known increment number to an accuracy of $\pm$ 0.5. Estimates were considered robust for each count as long as the standard deviation for the 30 counted sine wave patterns was < 1, as values above this may produce estimated increment counts of over 1 year above/below known/expected counts (see Supplement Section 2 'Robustness testing for increment counting algorithm').

2.7. Application of cementum increment counting algorithm

The increment counting algorithm presented here was used to generate estimates of increment counts for straightened and filtered CT slices for each lower first molar specimen of the 10 *Macaca mulatta* individuals. We applied the cementum increment counting algorithm to 30 CT slices for each individual, representative of highest cementum increment contrast and quality for each individual [6]. Each straightened and filtered CT dataset was examined by eye, in order to find the regions of highest increment contrast and minimum amounts of complexity in increment patterns (i.e. splitting and coalescence of increments). Slices were

selected contiguously through these regions until a number of 30 slices had been reached. 1000 transects were plotted through the cementum in each CT slice, and increment pair counts were generated for each transect. The mean increment pair count for all 1000 transects was then used as the estimated increment pair count for the slice, and the mean count of the 30 slices rounded to the nearest integer was defined as the estimated increment pair count for that *Macaca mulatta* individual.

3. Results

3.1. Optimisation of cementum imaging

When the X-ray energy was changed in isolation, SNR became consistently lower with increasing X-ray energy, whereas CNR peaked at 20 keV, before steadily falling with increasing X-ray energy beyond this point (Fig. 4a). SNR and CNR steadily improved with increasing exposure time (Fig. 4b). SNR and CNR also improved with increased number of projections, although the relative increase in CNR was marginal between 3001 and 4501 projections (Fig. 4c). SNR steadily increased with larger sample-to-detector distances up to 60 mm (Fig. 4d). Whereas CNR steadily rose from 14 mm sample-to-detector distance to a peak at 28 mm, it fell between a sample-to-detector distance of 28 mm and 100 mm (Fig. 4d).

The image quality of the dataset imaged at 28 mm sample-to-detector distance (Fig. 3c) represents an optimum in the trade-off between spatial resolution and contrast for our application. The smoothing inherent in the Paganin phase retrieval algorithm [35] flattens out increment boundaries and reduces noise, while (mean) greyscale differences are retained between light and dark increments to an extent that offers sufficient image contrast to identify individual cementum increments. For datasets created using smaller sample-to-detector distances (14 mm - 20 mm), high

image contrast resulted between increments, but their boundaries were smoothed and less well defined (case Fig. 3b). For sample-to-detector distances above this, the increasing amounts of smoothing diminished the differences in greyscale values between light and dark increments such that by 100 mm sample-to-detector distance, they were difficult to distinguish by eye (case Fig. 3d). This can also be shown quantitatively by plotting greyscale values along transects through the same region of cementum in each dataset (Fig. 3e) acquired at different sample-to-detector distances.

3.2. Cementum imaging results

Cementum was clearly visible in each CT dataset as an incremental tissue wrapping around the dentine of tooth roots and comprising a series of radial increments (Figs. 5 and 7-8). The cementum could be distinguished from the dentine due to its significantly lower mean grey values, and the cemento-dentine boundary was marked by the characteristic tissues of the granular layer of Tomes and the high-density hyaline layer of Hopewell Smith (Fig. 7). Individual increments were clearly visible within the cementum and could be followed through the entire dataset, both transversely and longitudinally (Fig. 8).

Comparison between CT slices and histological thin-sections of the same regions of cementum (created using the method outlined in Newham et al. [31] and imaged using the method outlined in Supplement Section 5 ‘Thin-section Imaging’) suggests that both imaging techniques represent the same cementum increments (Fig. 7; Supplement Fig. S4). Optical differences between increments in histological data were reflected as grey value differences in CT data. Thick, light increments in histological data corresponded to thick, light increments in CT data, and so absorbed a higher proportion of X-rays relative to thin, dark increments (Fig. 7). Volumetric CT data could further be used to help elucidate primary increments from accessory

increments in several specimens (Fig. 8). Complexities in increment patterns were witnessed intermittently in every *Macaca mulatta* individual, with individual increments splitting and coalescing to create apparent accessory increments. Following Newham et al. [31,32], individual increments could be mapped through the cementum tissue, and the same primary increments could be plotted through the entire scanned tissue volume (approximately 4 mm^3) (Fig. 8) across these complexities, and distinguished from the accessory increments created. Therefore, regions that were confounded by splitting and coalescing of these increments could be distinguished and excluded for analysis of increment counts (Fig. 8). Also, CIFC, the tissue with the least chronological precision in its increment periodicity [6], could be distinguished from acellular cementum by the presence of cellular voids, and so could be avoided when identifying high-contrast regions of increments with a circum-annual periodicity (Fig. 7). These two factors, aided by the entire coronal (crownward) third of the cementum tissue being imaged, led to the identification of the highest quality regions of circum-annual cementum increments for each specimen (Fig. 7).

3.3. Validation of cementum increment counting algorithm

Robustness tests for the proposed increment counting algorithm suggest that it is reliable for SNRs down to 0.2 (Fig. 6). For each simulated pattern of known increment number, the average value of 30 automated counts was identical to the known count for SNRs between 0.9-0.5. The upper/lower standard deviations of these samples did not exceed one integer above/below the known count (Fig. 6). Between SNR levels of 0.5-0.2, average automated counts only differed from known increment number by a value of one in a single sample (with a known increment number of eight). The standard deviations of automated counts exceed one integer above/below the known count for known increment counts of 22 and 28 (Fig. 6). SNRs of 0.1

introduced more errors of between one and two in increment count when compared to the known increment number, and the automated count was outside the region of one standard deviations around the known increment number (Fig. 6).

When increments were algorithmically counted in our macaque data and compared to expected counts for our sample based on known age, a Spearman's r of 0.77 (p<0.009) and Kendall's τ of 0.71 (p = 0.004) suggest significant correlation between semi-automated increment pair counts and expected numbers of cementum increment pairs. The mean of the semi-automated increment pair counts for each *Macaca mulatta* individual either met the minimum or maximum expected count based on their known age or fell in-between the two for every sample, apart from the juvenile individual t46 whose mean estimated count was 0.5 years more than the maximum expected count (Table 1 and Fig. 9). Juvenile cementum has been previously shown to contain more complex incrementation and greater amounts of increment splitting and coalescence than adult cementum [45]. Standard deviations of increment pair counts (average = 0.83) for the 30 individual CT slices examined for each individual did not exceed one for any *Macaca mulatta* individual. This suggests a precision of within one year for estimated increment counts using the proposed cementum increment counting algorithm.

4. Discussion

4.1. Image quality of SR CT data and optimisation of cementum imaging

The positive relationship observed between both SNR and CNR with increasing exposure time and number of projections has been expected following SR CT imaging of other hard tissues [24, 46]. The opposite relationship seen between SNR and X-ray energy can also be explained by a diminished X-ray absorption with

increased X-ray energy due to an exponentially decreased probability of photoelectric interactions between X-rays and the tissue. CNR has a more complex relationship with each experimental setting, with an optimum setting at a different level compared to SNR for each experimental setting. For instance, we located the optimal energy at TOMCAT for cementum increments in terms of CNR at around 20-21 keV, while SNR was highest at 19 KeV and continuously decreased with higher X-ray energies.

The steady increase in SNR with increasing sample-to-detector distance is in agreement with the results of Kitchen et al. [34], but in contrast to the results of Zeller-Plumhoff et al. [35]. Instead, Zeller-Plumhoff et al. [35] found that SNR of PPCI SR CT data of muscle tissue steadily decreased when sample-to-detector distance was increased between 30 mm and 60 mm at TOMCAT. The main factors responsible for the increase in SNR with increasing sample-to-detector distance in our study are the steady decrease in the standard deviation of the image background (σ_b) with increasing sample-to-detector distance versus the peak in mean greyscale value of cementum ($\overline{g_c}$) between 28-60 mm (Fig. 4e). The Paganin phase retrieval algorithm acts as a low pass filter, reducing the image noise in the resultant CT reconstructions. This filtering has been enhanced here with increased sample-to-detector and hence propagation distance, as shown by Kitchen et al. [34]. The reason for the different patterns encountered in SNR between the results of Kitchen et al. [34] and those of Zeller-Plumhoff et al. [35] were attributed by Zeller-Plumhoff et al. [35] to be due to different targets in terms of image quality, when considering the optimal ratio of δ/β for the Paganin phase retrieval algorithm. The objective of Kitchen et al. [34], and of our study, was primarily to enhance image contrast within the PPCI SR CT data, whereas Zeller-Plumhoff et al. [35] also considered the sharpness of feature boundaries when optimising δ/β. Moreover, the material

properties of cementum are different to the soft tissues studied by both Kitchen et al. [34] (lung tissue) and Zeller-Plumhoff et al. [35] (muscle tissue).

It is important to remember that, while our optimisation study was focused on image quality, other factors play important roles for the PPCI of cementum. Firstly, cementum has recently been suggested as an important reservoir of ancient DNA (aDNA) in sub-fossil and archaeological specimens, including early hominins such as Neanderthals [47]. It is also well understood that the significant X-ray doses provided by low energy and/or sub-micrometre resolution PPCI is sufficient to denature and destroy aDNA within the scanned ROI [47]. Hence, the optimum experimental settings presented here may well involve an X-ray dose that is too high to be viable for specimens bound for aDNA extraction and study [30].

Further, while increasing the number of angular projections increases image quality, it also increases the total acquisition time of each CT scan. As each synchrotron experiment is awarded a restricted amount of beam time, optimal experimental setings have to be weighed up against scanning time to image a sufficient sample of specimens. Hence, given the time allocated for our experiment, we retained 1501 projections per CT scan, half of the optimal value around 3000-4000 in terms of SNR (Fig. 4), in order to image our complete sample within the allotted beamtime.

4.2. X-ray PPCI versus thin-section imaging for counting cementum increments

The first objective of this study was to optimise PPCI through SR CT for studying cementum increments in 3D tomographic data, as an alternative strategy to destructive thin-sectioning and light microscopy for imaging and counting cementum increments. We have shown here that optimised PPCI strategies can overcome the principal caveats of thin-section imaging: namely the destructive sample preparation

process (with the caveat that we isolate both tooth roots from the crown) and the limited 2D view of tissue that is actually 3D in nature, so lacking context for interpreting complexities in increment patterns; and also limited control over which cementum tissue type is imaged (AEFC versus CIFC). The high image quality offered by SR CT, including phase retrieval offered in PPCI, has provided comparable fidelity for counting individual cementum increments to thin-section histological images of the same regions. This is of particular note given that the study sample originates from a captive population with minimal seasonal environmental and nutritional variation. It is understood that the cementum of captive mammals presents lower increment contrast than free-roaming populations [1], and so the identification and accurate counting of circum-annual increments of PPCI data in captive cementum may be another promising element of this technique. This also adds to the body of work finding circum-annual increments in modern humans ([6] and references therein), suggesting that incrementation follows an endogenous (potentially hormone-driven; see Newham et al. [30]) rhythm as opposed to primarily reflecting seasonal dietary differences [4].

The volumetric nature of CT datasets allows navigation through the entire cementum tissue at an isotropic and sub-micrometre nominal spatial resolution. Individual cementum increments can be followed across regions exhibiting complex cementum patterns, created by splitting and coalescence of increments, and regions of CIFC can be avoided when analysing AEFC. This minimises the potential for inaccurate increment counting. As a non-destructive technique the use of PPCI through SR CT for cementochronology permits the study of cementum in specimens previously beyond the reach of traditional histological analyses that are destructive, including fossils [32] and archaeological specimens [28-30]. As there is no physical

thin-sectioning of the tissue involved for CT, images are not affected by tissue preparation artefacts such as scratches on the ground and polished thin section or tissue distortion through the mechanical cutting process, which can obscure or alter image details on cementum increments [6,14].

However, our PPCI of cementum through SR CT has also highlighted the sensitivity of cementum image quality to experimental settings. This, alongside other factors described in Section 4.1., suggests that optimisation of experimental settings should be conducted preliminary to every cementochronological PPCI experiment using SR CT, in order to ensure optimised image quality for identifying and counting cementum increments within the constraints given by the broader aims of the particular experiment [30,47]. Optimal experimental settings are specific to the optics of the synchrotron beamline and the material properties, size and morphology of the specimen, and so should be investigated when any of these factors are changed.

It also became apparent during scanning that micrometre-scale cracks, which are not visible macroscopically, have formed within the cementum tissue (Supplementary Fig. S5), most probably due the interaction of the hard X-rays with residual water left within the teeth and/or related effects due to this interaction [47]. Although this damage could not be seen macroscopically, comparison with the lack of observations of any damage in archaeological [30] and fossil [32] teeth confirms that the preparation of “fresh” teeth was suboptimal for SR CT scanning, as residual water was retained. Further, it cannot be confirmed whether micro-cracks occurred solely during the scanning process, or started due to demineralisation during fixation in paraformaldehyde. This should be examined in future studies via microscopic examination of the cementum of fixed teeth. Moreover, further optimisation of the

preparation procedure for fresh teeth is still needed, that ensures both minimal risk of biological contamination and minimal damage to the tissue during CT scanning.

4.3.Cementum increment counting algorithm

The second objective of this study was to provide a validated, semi-automated and robust algorithm for user-independent counting of cementum increments. The manual counting of cementum increments amongst a restricted number of thin-sections per tooth, plays a central role in the current user-dependent approach for counting cementum increments. This subjectivity has led to a wide range of different accuracies and precisions reported for increment counts and their correlation with known age in animal and human samples. Both accuracy and precision in estimated increment counts correlate with the experience of the researcher when compared to known age (i.e. expected increment count) in validation studies [6].

Our algorithm offers a new method for objectively counting cementum increments in a user-independent and semi-automated fashion. This substantially decreases the subjectivity and propensity for human error involved in increment counting (see Supplement section 6. 'Testing the effects of variability in straightening on increment counting'). Within the same selected sample of straightened, isolated and filtered PPCI slices, our algorithm requires no further human input for counting cementum increments and will estimate the same increment count regardless of the experience of the researcher. The accuracy and precision of this algorithm has been validated here for both simulated data and our experimental sample of *Macaca mulatta* cementum (in both 8-bit and 16-bit data; see Supplement Section 1). It could also be further assessed in the same quantitative manner with other PPCI cementum data from animals of known age. Such assessment will allow for further optimisation of our algorithm and tailoring for a wide range of PPCI cementum data.

Finally, although we state the advantages of PPCI imaging over traditional thin-section histological imaging here, the validation of our application on thin-section data of cementum from animals of known age may afford its application for thin-section images. If found to be an accurate method for counting thin-section increments, implementation of our algorithm for thin-sections has potential as an important tool for validating the accuracy of counts estimated by-eye, or even discounting the need for counting increments by-eye completely.

5. Conclusion

In conclusion, we have undertaken a first systematic experimental study on cementum increment counting for non-fossilised dental tissue, based on a comparison between optimised PPCI through SR CT and thin-section histological imaging. Comparison between these two imaging techniques has shown that PPCI SR CT data can provide sufficient spatial resolution and image contrast to reproduce individual growth increments in the cementum tissue (as well as accessory increments that can be followed through their own trajectory and discounted, thanks to the 3D nature of CT data). CT reconstructions are of sufficient quality to count increments semi-automatically using image processing, by defining them as peaks and troughs in greyscale values along transects through the cementum. We have implemented this semi-automated method of increment counting as part of a novel workflow of image processing (cementum isolation, straightening and filtering) and analysis (application of a purpose-built increment counting algorithm). This may help future studies to overcome the central caveat facing current studies of cementum increments: the subjectivity inherent in counting increments by eye that depends on the individual researcher. The combination of non-destructive imaging and objective increment

counting may open up a new range of specimens, samples and studies not suitable for destructive thin-section analysis, and help to exploit the potential of cementum as a record of life history for archaeology, anthropology, forensic science and palaeontology.

Acknowledgments and funding: This study was part-funded by a Natural Environmental Research Council/Engineering and Physical Sciences Research Council doctoral candidateship (UK; grant number NE/R009783/1). Funding was also provided by Ginko Investments Ltd (Bristol, UK) for purchasing external hard drives and funding travel to the Swiss Light Source, and the Academy of Finland. We acknowledge the Paul Scherrer Institute, Villigen, Switzerland for provision of synchrotron radiation beamtime at the TOMCAT beamline of the SLS (Experiment 20151391) and would like to thank Iwan Jerjen, Mark Mavrogordato, Orestis Katsamenis, Sharif Ahmed, Christianne Fernee Juan Núñez, and Priscilla Bayle for their assistance during our beamtime.

Conflict of interest. The authors report no conflicts of interest. The funders had no role in study design, data collection and analysis, decision to publish, or preparation of the manuscript. The private funding received from Ginko Investments Ltd does not alter our adherence to PLOS ONE policies on sharing data and materials.

Author contributions. EN designed, validated and performed all analyses. All authors were involved in writing the proposal for synchrotron beamtime and synchrotron imaging. All authors contributed to drafting and revising the manuscript.

Data accessibility. All data supporting this study are openly available from the University of Southampton repository (“https://doi.org/10.5258/SOTON/D1722).

6. References

1. Klevezal G. Recording structures of mammals. Boca Raton, Florida: CRC Press; 1996.
2. Foster BL. On the discovery of cementum. J. Periodontal Res. 2017;54:666-685.
3. Stock SR, Finney LA, Telser A, Maxey E, Vogt S, Okasinski JS. Cementum structure in Beluga whale teeth. Acta biomater. 2017;4:289-299.
4. Lieberman DE. Life history variables preserved in dental cementum microstructure. Science. 1993;261(5125):1162-1164.
5. Dean C, Le Cabec A, Spiers K, Zhang Y, Garrevot J. Incremental distribution of strontium and zinc in great ape and fossil hominin cementum using synchrotron X-ray fluorescence mapping. J. R. Soc. Interface. 2018;15(138):20170626.
6. Naji S, Colard T, Blondiaux J, Bertrand B, d'Incau E, Bocquet-Appel JP. Cementochronology, to cut or not to cut? Int. J. Paleopathol. 2016;15:113-119.
7. Renz H, Radlanski RJ. Incremental lines in root cementum of human teeth – a reliable age marker? Homo. 2006;57(1):29-50.
8. Kasetty S, Rammanohar M, Raju Ragavendra T. Dental cementum in age estimation: a polarized light and stereomicroscopic study. J. Forensic Sci. 2010;55(3): 779-783.
9. Stutz AJ. Polarizing microscopy identification of chemical diagenesis in archaeological cementum. J. Archaeol. Sci. 2002;29(11):1327-1347.
10. Lieberman DE, Deacon TW, Meadow RH. Computer image enhancement and analysis of cementum increments as applied to teeth of *Gazella gazella*. J. Archaeol. Sci. 1990;17(5):519-533.

11. Wall- Scheffler CM, Foley RA. Digital cementum luminance analysis (DCLA): a tool for the analysis of climatic and seasonal signals in dental cementum. Int. J. Osteoarchaeol. 2008;18(1):11-27.
12. Lipsinic FE, Paunovich E, Houston GD, Robison SF. Correlation of age and incremental lines in the cementum of human teeth. J. Forensic Sci. 1986;31(3):982-989.
13. Obertová Z, Francken M. Tooth cementum annulation method: accuracy and applicability. In Koppe T, Meyer G, Alt KW, Brook A, Dean MC, Kjaer I, et al. editors. Comparative dental morphology Vol. 13. Basel, Switzerland; 2009. p. 184-189.
14. Czermak A, Czermak A, Ernst H, Grupe G. A new method for the automated age-at-death evaluation by tooth-cementum annulation (TCA). Anthropol. Anz. 2006;64(1):25-40.
15. Klauenberg K, Lagona, F. Hidden Markov random field models for TCA image analysis. Computational Statistics & Data Analysis. 2007;52(2):855-868.
16. Schneider CA, Rasband WS, Eliceiri KW. NIH Image to ImageJ: 25 years of image analysis. Nat. Methods. 2012;9(7):671.
17. Schneider P, Stauber M, Voide R, Stampanoni M, Donahue R, Müller R. Ultrastructural properties in cortical bone vary greatly in two inbred strains of mice as assessed by synchrotron light based micro- and nano- CT. J. Bone Miner. Res. 2007;22(10):1557-1570.
18. Schneider P, Krucker T, Meyer E, Ulmann- Schuler A. Weber B, Stampanoni M, et al. Simultaneous 3D visualization and quantification of murine bone and bone vasculature using micro- computed tomography and vascular replica. Microsc. Res. Tech. 2009;72(9):690-701.

19. Sanchez S, Ahlberg PE, Trinajstic KM, Mirone A, Tafforeau P. Three-dimensional synchrotron virtual paleohistology: a new insight into the world of fossil bone microstructures. Microsc. Microanal. 2012;18(5):1095-1105.
20. Goggin PM, Zygalakis KC, Oreffo RO, Schneider P. High-resolution 3D imaging of osteocytes and computational modelling in mechanobiology: insights on bone development, ageing, health and disease. Eur. Cells and Mater. 2016;31:264-95.
21. Núñez JA, Goring A, Hesse E, Thurner PJ, Schneider P, Clarkin CE. Simultaneous visualisation of calcified bone microstructure and intracortical vasculature using synchrotron X-ray phase contrast-enhanced tomography. Sci. Rep. 2017;7(1):13289.
22. Goring A, Sharma A, Javaheri B, Smith RC, Kanczler JM, Boyde A, et al. Regulation of the bone vascular network is sexually dimorphic. J. Bone Miner. Res. 2019; DOI:10.1002/jbmr.3825.
23. Tafforeau P, Boistel R, Boller E, Bravin A, Brunet M, Chaimanee Y, et al. Applications of X-ray synchrotron microtomography for non-destructive 3D studies of paleontological specimens. Appl. 2006;83(2):195-202.
24. Tafforeau P, Bentaleb I, Jaeger JJ, Martin C. Nature of laminations and mineralization in rhinoceros enamel using histology and X-ray synchrotron microtomography: potential implications for palaeoenvironmental isotopic studies. Palaeogeogr. Palaeoclimatol. Palaeoecol. 2007;246(2-4):206-227.
25. Tafforeau P, Smith TM. Nondestructive imaging of hominoid dental microstructure using phase contrast X-ray synchrotron microtomography. J. Hum. Evol. 2008;54(2):272-278.

26. Le Cabec A, Tang N, Tafforeau P. Accessing developmental information of fossil hominin teeth using new synchrotron microtomography-based visualization techniques of dental surfaces and interfaces. PloS one. 2015;10(4):e0123019.
27. Green DR, Green GM, Colman AS, Bidlack FB, Tafforeau P, Smith TM. Synchrotron imaging and Markov chain Monte Carlo reveal tooth mineralization patterns. PloS one. 2017;12(10):e0186391.
28. Mani-Caplazi G, Schulz G, Deyhle H, Hotz G, Vach W, Wittwer-Backofen U, et al. Imaging of the human tooth cementum ultrastructure of archaeological teeth, using hard X-ray microtomography to determine age-at-death and stress periods. In Developments of X-ray tomography XI. International Society for Optics and Photonics. 2017;10391:103911C.
29. Newham E. Exploring the use of tomography for the quantification of cementum growth patterns across the mammal phylogeny. PhD Thesis, The University of Southampton. 2018.
30. Le Cabec A, Tang NK, Ruano Rubio V, Hillson S. Nondestructive adult age at death estimation: Visualizing cementum annulations in a known age historical human assemblage using synchrotron X- ray microtomography. Am. J. Phys. Anthropol. 2019;168(1);25-44.
31. Newham E. Corfe IJ, Brown KR, Gostling NJ, Gill PG, Schneider P. Synchrotron radiation-based X-ray tomography reveals life history in primate cementum incrementation. J. R. Soc. Interface. 2020a;17(172):20200538.
32. Newham E, Gill PG, Brewer P, Benton MJ, Fernandez V, Gostling N J, et al. Reptile-like physiology in Early Jurassic stem-mammals. Nat. Commun. 2020b;11(1):1-13.

33. Bowen WH, Koch G. Determination of age in monkeys (*Macaca irus*) on the basis of dental development. Lab. Anim. 1970;4(1):113-123.
34. Kitchen MJ, Buckley GA, Gureyev TE, Wallace MJ, Andres-Thio N, Uesugi K, et al. CT dose reduction factors in the thousands using X-ray phase contrast. Sci. Rep. 2017;7(1):15953.
35. Zeller-Plumhoff B, Mead JL, Tan D, Roose T, Clough GF, Boardman RP, et al. Soft tissue 3D imaging in the lab through optimised propagation-based phase contrast computed tomography. Opt. Express. 2017;25(26):33451-33468.
36. Paganin D, Mayo SC, Gureyev TE, Miller PR, Wilkins SW. Simultaneous phase and amplitude extraction from a single defocused image of a homogeneous object. J. Microsc. 2002;206(1):33-40.
37. Keyriläinen J, Bravin A, Fernández M, Tenhunen M, Virkkunen P, Suortti P. Phase-contrast X-ray imaging of breast. Acta radiol. 2010;51(8): 866-884.
38. Marone F, Stampanoni M. Regridding reconstruction algorithm for real-time tomographic imaging. J. Synchrotron Radiat. 2012;19:1029-1037.
39. Nixon M, Aguado AS. Feature extraction and image processing for computer vision. Cambridge, Massachusetts: Academic Press; 2012.
40. Lanman D. Steerable Gaussian Filters (https://www.mathworks.com/matlabcentral/fileexchange/9645-steerable-gaussian-filters), MATLAB Central File Exchange. Retrieved July 16, 2021.
41. Freeman WT, Adelson EH. The Design and Use of Steerable Filters. IEEE Transactions on Pattern Analysis and Machine Intelligence. 1991;13(9):891–906.
42. Gadelmawla ES, Koura MM, Maksoud TMA, Elewa IM, Soliman HH. Roughness parameters. Journal of materials processing Technology.2002;123(1):133-145.

43. Esfahani M, Munir KS, Wen C, Zhang J, Durandet Y, Wang J, et al. Mechanical properties of electrodeposited nanocrystalline and ultrafine-grained Zn-Sn coatings. Surf. Coat. Technol. 2018;333:71–80.

44. Martinez WL, Martinez AR. Computational statistics handbook with MATLAB. Boca Baton, Florida: Chapman and Hall/CRC Press; 2015.

45. Klevezal, G., Stewart, B. S. Patterns and calibration of layering in tooth cementum of female northern elephant seals, *Mirounga angustirostris. J. Mammal.* 1994;75(2):483-487.

46. Bouxsein, M. L., Boyd, S. K., Christiansen, B. A., Guldberg, R. E., Jepsen, K. J., Müller, K. J. Guidelines for assessment of bone microstructure in rodents using micro-computed tomography. J. Bone Miner. Res. 2010;25(7):1468-1486.

47. Immel A, Le Cabec A, Bonazzi M, Herbig A, Temming H, Schuenemann VJ, et al. Effect of X-ray irradiation on ancient DNA in sub-fossil bones–Guidelines for safe X-ray imaging. Sci. Rep. 2016;6:32969.

Figure captions

Figure 1. Histological data of rhesus macaque (*Macaca mulatta*) cementum in the permanent lower first molar tooth.

(**a**) 70 µm-thick transverse reflected light digital micrograph of the cementum tissue from the coronal third of the tooth root at 20× magnification. Cementum (C) is defined as the tissue wrapping around the circumference of the dentine (D), comprising a series of circumferential increments. Blue dashed line highlights a surface scratch created during the thin-sectioning process. (**b**) Detail marked by red dashed box in (**a**) displaying cementum increments in acellular extrinsic fibre cementum, and cellular voids left by cementoblasts in cellular intrinsic fibre cementum (CIFC) under higher

resolution (50× magnification). White arrows highlight 'light' increments, blue arrows highlight 'dark' increments, and red arrows highlight cementoblasts. Red bracketed lines highlight cellular intrinsic fibre cementum. Green bracketed line highlights the hyaline layer of Hopewell-Smith, demarcating the cemento-dentine boundary. Scale bar in (**a**) represents 100 μm. Scale bar in (**b**) represents 30 μm.

Figure 2. Tomographic cementum increment counting. (**a**) Straightened, filtered PPCI SR CT image of *Macaca mulatta* cementum (see Fig. 5). (**b**) Plot of greyscale values along transect highlighted by the 10 pixel-thick coloured band in (**a**) with outer-cementum surface highlighted with orange asterisk and cemento-dentine boundary by red asterisk. Transects are split into five sections. Light/dark increment pairs are distinguished as peak/trough systems in greyscale values, where both the peak and trough depart from the mean greyscale value of that section (red line), beyond the half standard deviations of greyscale values within the section above the mean (green line) and below the mean (blue line), respectively. (**c**) Sections split into their upper and lower datasets comprising of peaks and troughs in greyscale that exceed beyond the upper and lower standard deviation (respectively). Here, peak/trough pairs are counted, denoted with red numbers. Troughs and peaks that are not counted are denoted in blue numerals, as they either do not exceed the standard deviation of that section or are less than three pixels away from the last respective peak/trough. (**d**) Resulting increment pair counts for each section as seen in (**b**).

Figure 3. SR CT scan of a tooth root and regions for signal-to-noise ratio (SNR) and contrast-to-noise ratio (CNR) calculations. (**a**) One CT slice of the tooth root of *Macaca mulatta* individual l56. Blue box highlights the ROIfor

evaluation of background signal from which the mean greyscale value ($\overline{g_b}$) and standard deviation of greyscale values (σ_b) was generated for SNR and CNR calculations (see Equations (1) & (2)). The green box highlights the sampling area for cementum signal from which the mean greyscale value ($\overline{g_c}$) was generated for SNR and CNR calculations. Dashed red boxes indicate regions highlighted of the detail views. (**b**) Detail from region indicated by dashed red boxes in (**a**) from the dataset acquired at 16 mm sample-to-detector distance. (**c**) Detail from the same region from the dataset acquired at 28 mm sample-to-detector distance. (**d**) Detail from the same regionfrom the dataset acquired at 100 mm sample-to-detector distance. (**e**) Plots of greyscale values along transects indicated by dashed lines in (**b-d**), acquired at different sample-to-detector distances. (**a-d**) White scale bars in (**a**) represent 100 µm in the overview image, 30 µm in the detail view highlighted by the red dashed box, and 10 µm in (**b-d**).

Figure 4. SNR and CNR for sweep of experimental settings for PPCI through SR CT. Data shown is from specimen l56. (**a**) SNR (shown in black) and CNR (shown in red) values for different X-ray energies. (**b**) SNR and CNR values for different exposure times. (**c**) SNR and CNR values for different numbers of angular projections. (**d**) SNR and CNR values for different sample-to-detector distances. (**e**) Relationship between the standard deviation of background (σ_b – shown in black) and the mean of cementum signal ($\overline{g_c}$ – shown in red) with increasing sample-to-detector distance.

Figure 5. Image processing of PPCI SR CT images of *Macaca mulatta* cementum. (**a**) One CT slice of specimen l59. (**b**) Detail of CT slice highlighting circumferential

cementum increments (cementum defined by dashed yellow line) with midline shown as solid yellow line. (**c**) Straightened cementum section following the midline highlighted in (**b**). (**d**) Filtered image of (**c**) using a steerable Gaussian filter (see Supplement, section 1. 'Image processing by directional filtering'). White scale bar in (a) represents 200 µm; and in (b) represents 100 µm. Black scale bars in (c) and (d) represent 30 µm.

Figure 6. Robustness tests for algorithmic increment counts. (**a**) Counts (black circles) and their standard deviations (green boxes) for incremental sine wave patterns of 5 up to 30 increments with signal-to-noise ratios (SNRs) of 0.9-0.5. Inset: box displaying an example of a 10-increment pattern with an SNR of 0.5. (**b**) Counts and their standard deviations for incremental sine wave patterns of 5 up to 30 increments with SNRs of 0.4-0.2. Inset; box displaying an example of a 10-increment pattern with an SNR of 0.2. (**c**) Counts and their standard deviations for incremental sine wave patterns of 5 up to 30 increments with an SNR of 0.1. Inset: box displaying an example of a 10-increment pattern with an SNR of 0.1. Red dashed lines represent 1:1 correlation between estimated and known increment count.

Figure 7. Comparison between histological and CT data. (**a**) Detail of histological thin-section of the k49 specimen displaying 12 light increments indicated by blue arrows. (**b**) Detail of reconstructed CT slice of the same region as (**a**) displaying 11 cementum increments. (**c**) Detail of histological thin-section of the k16 specimen displaying eight light cementum increments. (**d**) Detail of reconstructed CT slice of the same region as (**c**) displaying eight increments. (**e**) Detail of histological thin-section of the l59 specimen displaying 11 light increments. (**f**) Detail of reconstructed

CT slice of the same region as (**e**) displaying 11 increments. (**g**) Detail of histological thin-section of the l56 specimen displaying 12 light increments. (**h**) Detail of reconstructed CT slice of the same region as (**g**) displaying 11 increments. See Supplementary Figure S4 for combination of histological/tomographic data for each specimen. Black scale bars represent 30 μm. Yellow whiskers highlight the granular layer of Tomes (labelled GLoT); pink dashed whiskers highlight the hyaline layer of Hopewell Smith (labelled HLHS); and red arrows highlight cellular voids within cellular intrinsic fibre cementum. Red dashed circles highlight surface damage created during thin-section processing.

Figure 8. 3D CT data of cementum increments. Data shown is from specimen k49. (**a**) Straightened and filtered CT slice displaying 10 increment pairs, highlighted with coloured arrows. (**b**) Schematic of detail from **a** highlighted by dashed red box, showing complexities in increment patterns. Increments are given the same colour as their respective arrows in **a**. Instances of splitting are highlighted with dashed red lines, and instances of coalescence by blue dashed lines. (**c**) 3D model of segmented cementum increment patterns plotted through the coronal third of the root imaged by PPCI through SR CT. Scale bars represent 100 μm.

Figure 9. Comparison between known increment pair counts and those estimated by the proposed cementum increment counting algorithm. PPCI SR CT data shown are from m1 teeth of ten *Macaca mulatta* individuals of known age (see Table 1)**.** Cyan boxes indicate the interquartile range around the mean estimated increment count, indicated by the thick black line. Whiskers represent the extreme lower and upper estimated counts. Blue circles indicate known age of each individual. Red

triangles indicate the maximum and inverted green triangles indicate the minimum expected increment count for each individual based on the approximate age-of-eruption of the m1 at 18 months of age in *Macaca mulatta*.

Table 1.

Specimen	Age (years)	DOB	DOD	Known increment pair count		Estimated increment pair count	
				Minimum	Maximum	Mean	Standard deviation
**k49**	12	09.04.03	08.04.15	10	11	10	0.95
**k91**	11.5	06.10.03	10.04.15	9.5	10.5	10	0.81
**k23**	12	09.03.03	09.04.15	10	11	10	0.89
**k24**	12	12.03.03	08.04.15	10	11	10	0.96
**l10**	11	20.02.04	08.04.15	9	10	9.5	0.94
**l14**	11	26.02.04	10.04.15	9	10	9.75	0.86
**l56**	11	14.04.04	10.04.15	9	10	10	0.92
**l59**	11	17.04.04	09.04.15	9	10	9.5	0.71
**k16**	10.5	16.09.04	09.03.15	8.5	9.5	9	0.52
**t46**	5.5	11.03.10	07.07.15	3.5	4.5	5	0.94

Table 1. Life history data, known increment pair counts and estimated increment pair counts for each of the 10 female rhesus macaque (*Macaca mulatta*) individuals studied. DOB = date of birth; DOD = date of death. Mean increment pair counts are rounded to the nearest 0.25 years for comparison with known increment pair counts.

Table 2.

Scan name	Energy (keV)	Exposure time (ms)	Number of projections	Sample-to-detector distance (mm)	Mean SNR	Mean CNR
SO20	19	150	1501	14	187.9	50.7
SO21	20	150	1501	14	123.8	56.9
SO22	21	150	1501	14	112.9	60.8
SO23	22	150	1501	14	82.6	35.5
SO24	26	150	1501	14	72.0	24.5
SO25	20	100	1501	14	118.3	50.7
SO26	20	125	1501	14	124.0	55.4
SO27	20	300	1501	14	177.8	72.3
SO28	20	150	3001	14	245.4	74.5
SO29	20	150	4501	14	252.7	81.5
SO30	20	150	6001	14	300.5	84.9
SO31	20	150	1501	16	142.2	59.0
SO32	20	150	1501	20	152.9	68.9
SO33	20	150	1501	28	185.6	75.2
SO34	20	150	1501	60	179.6	77.8
SO35	20	150	1501	100	247.4	54.9

Table 2. Image quality assessments of PPCI SR CT images using different experimental settings. SNR = signal-to-noise ratio; CNR = contrast-to-noise ratio.

Figure
Click here to access/download;Figure;FINAL plos one figure 1.pdf

Figure 1.

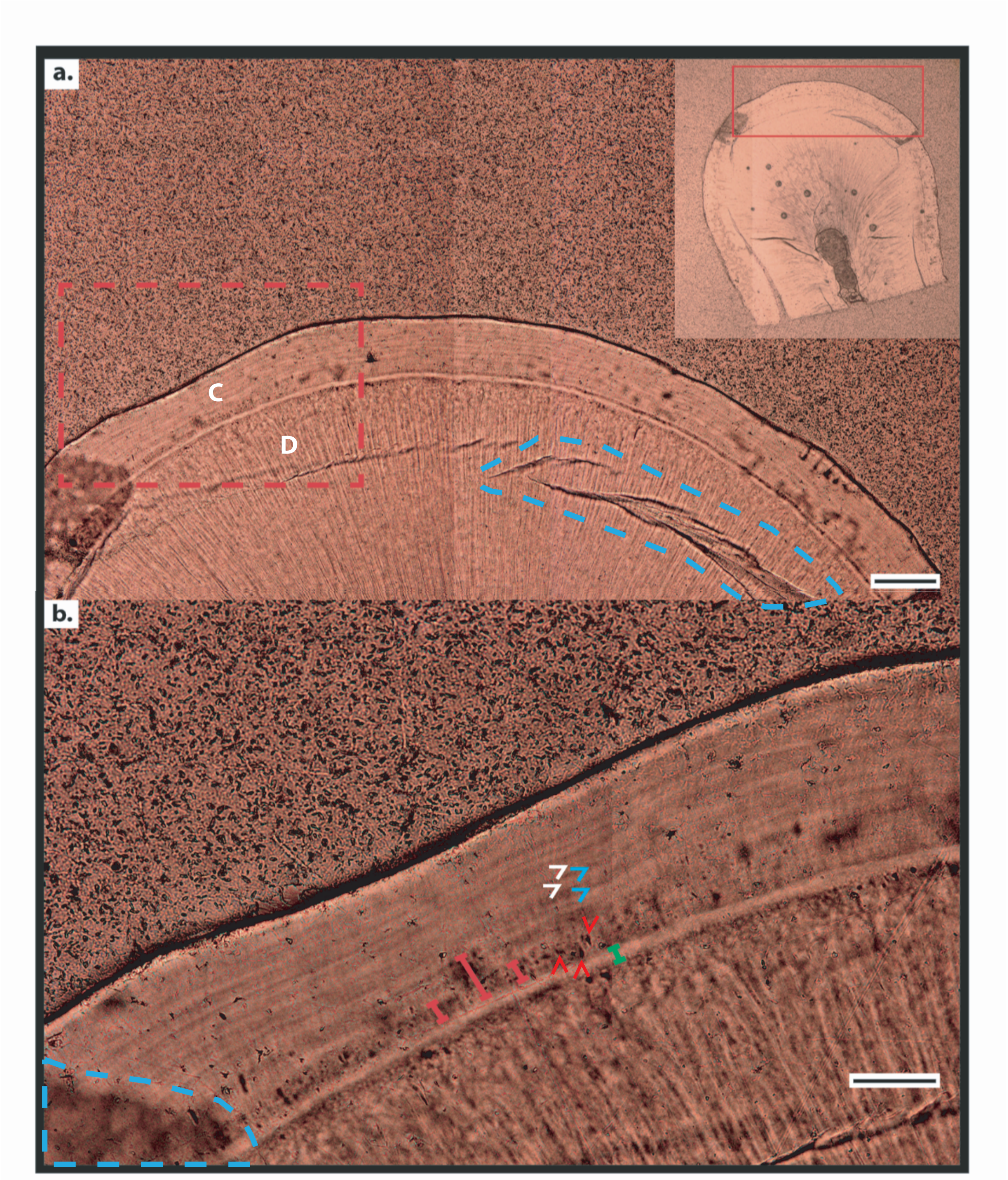

Figure

Click here to access/download;Figure;FINAL plos one figure 3.pdf

Figure 3.

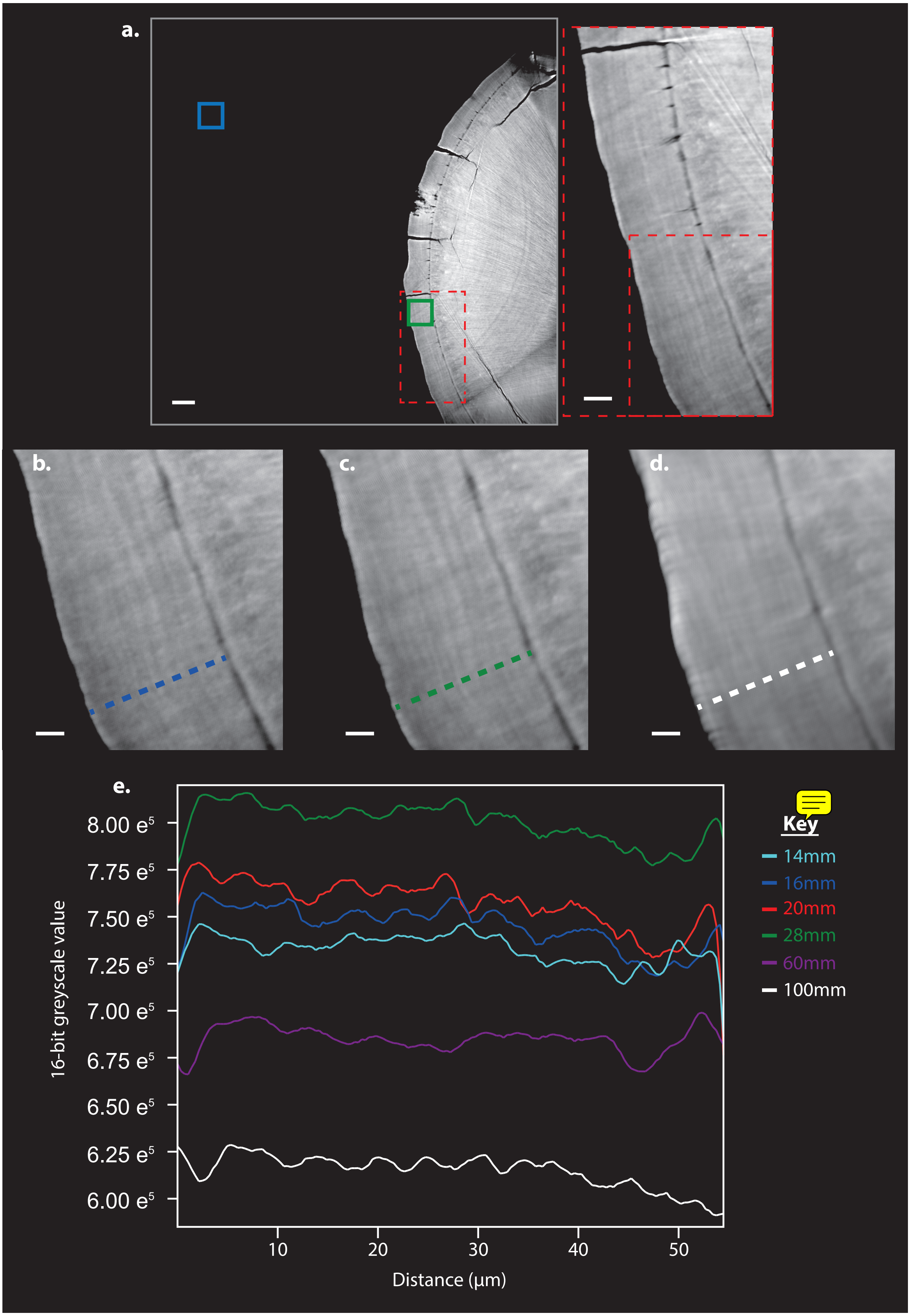

Figure

Click here to access/download;Figure;FINAL plos one figure 8.pdf

Figure 8.

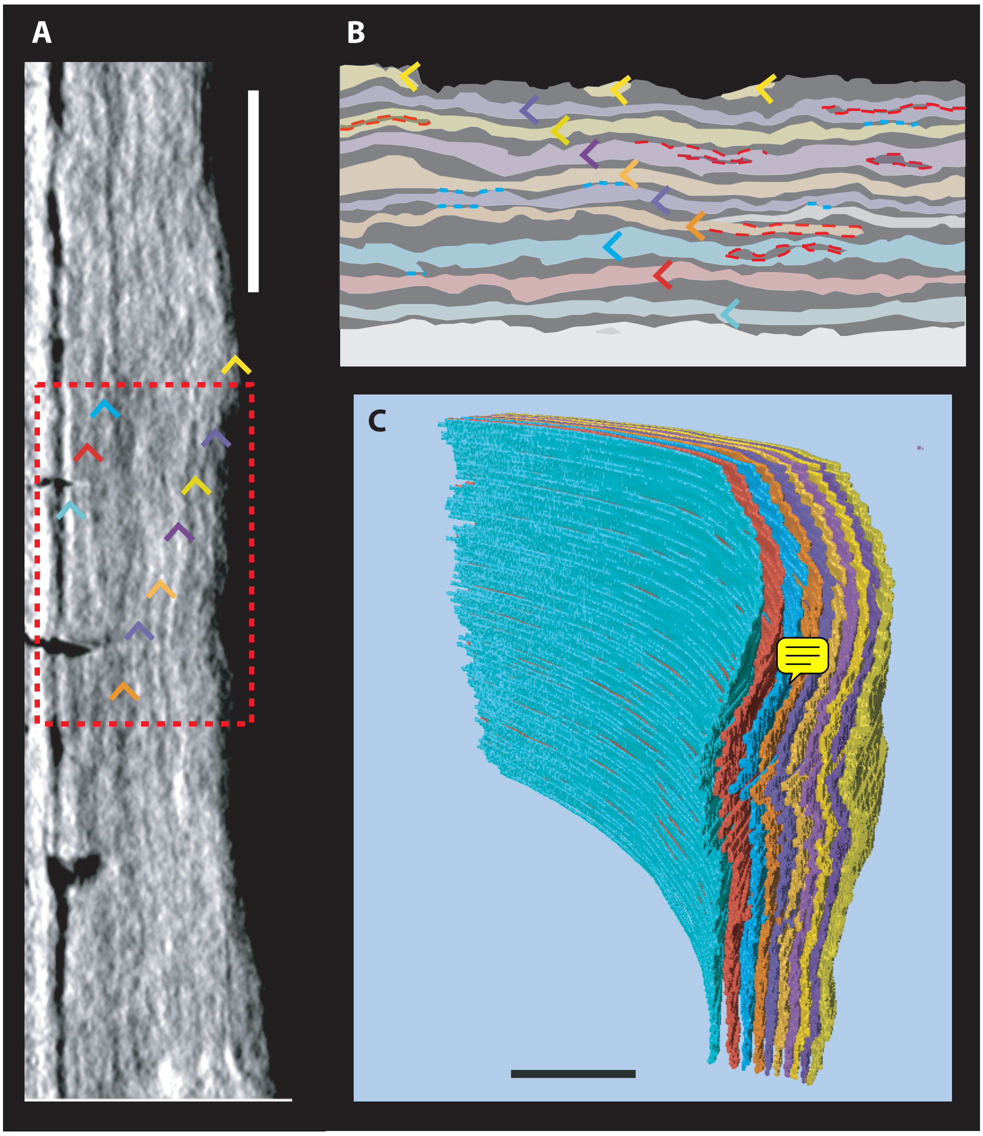

Figure
Click here to access/download;Figure;Revised figure 2.pdf

Figure 2.

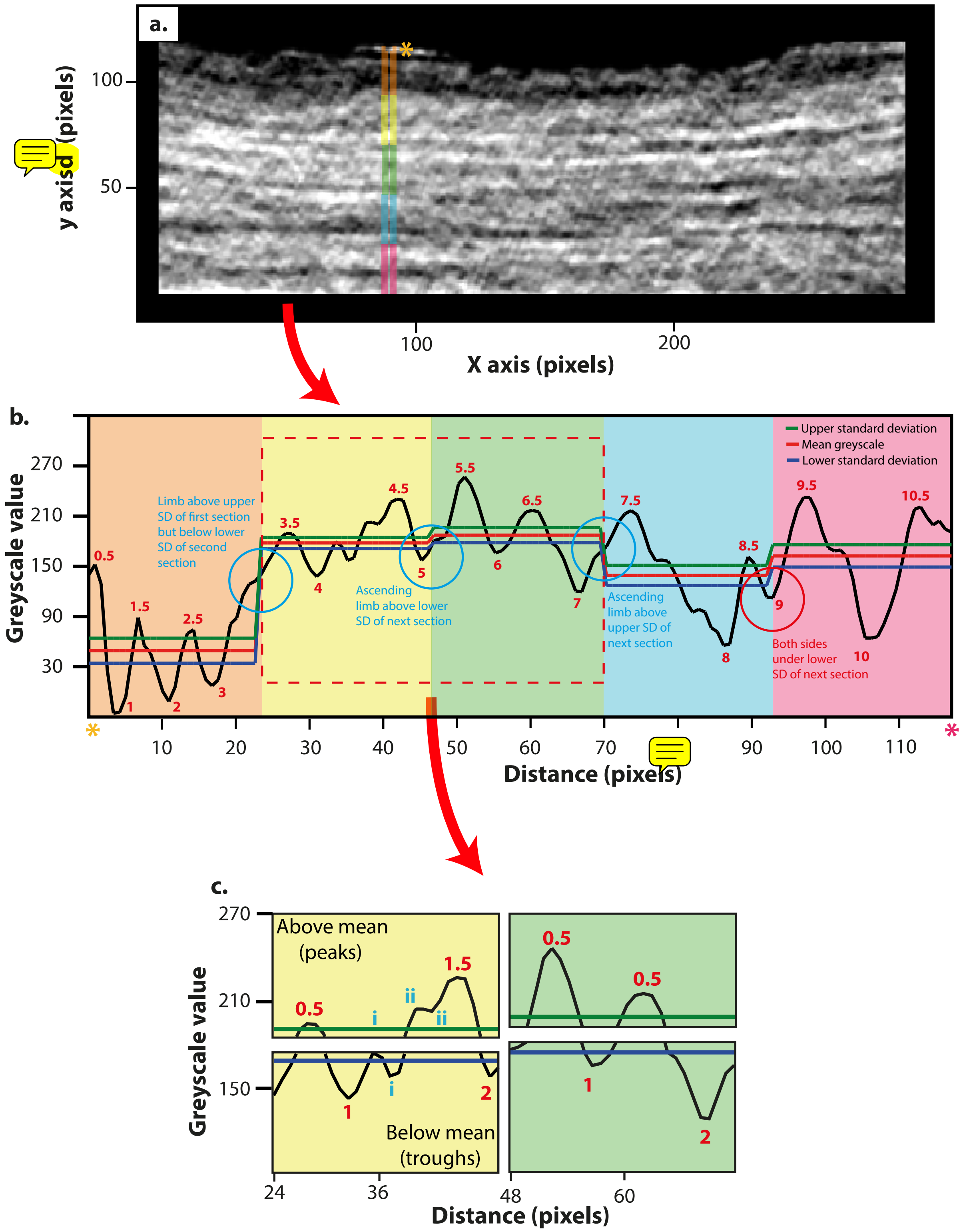

d.

Section	Increment pair count
1	3.0
2	2.0
3	2.0
4	1.5
5	2.0
Total	10.5

Figure
Click here to access/download;Figure;Revised figure 4.pdf

Figure 4.

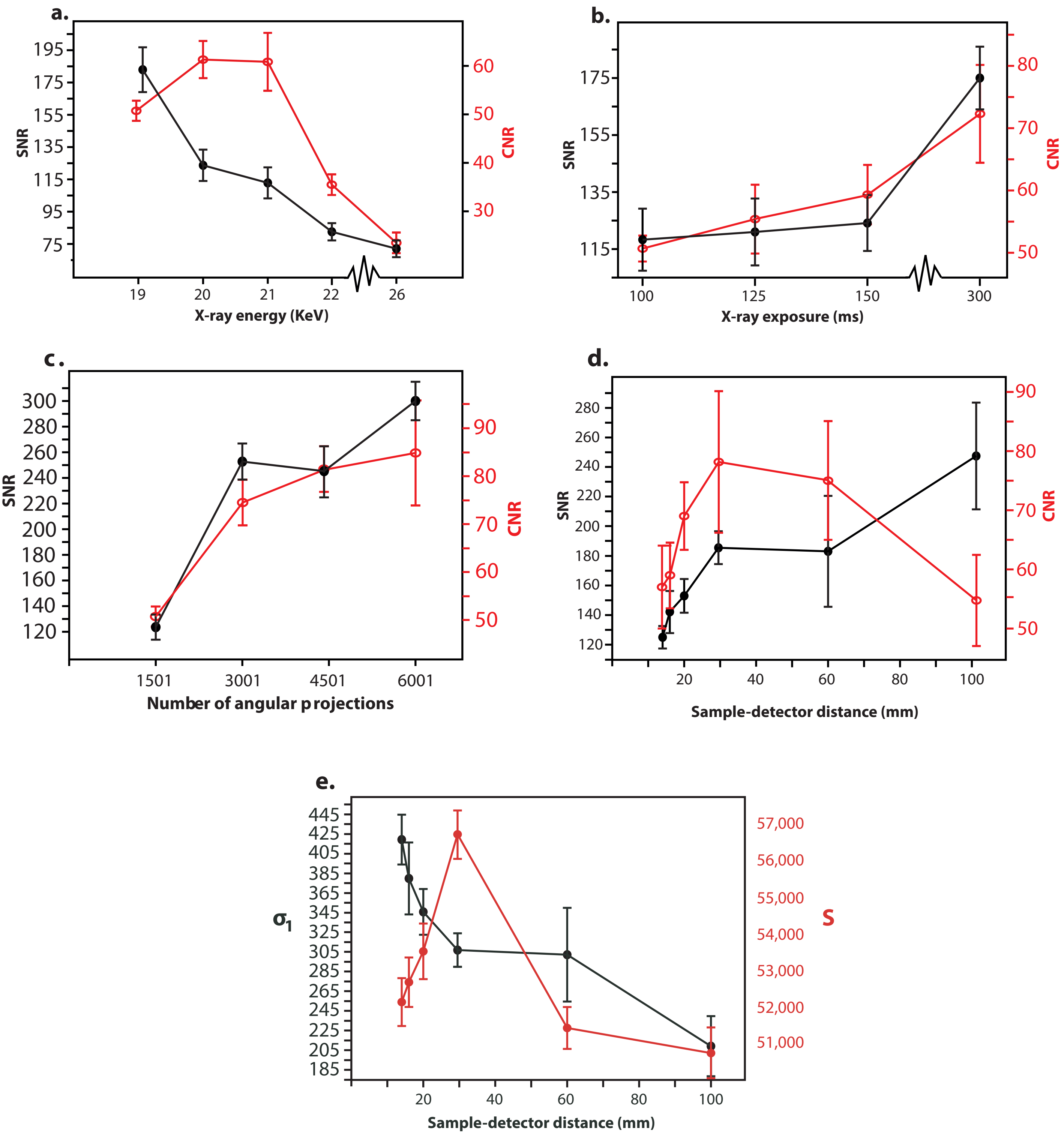

Figure Click here to access/download;Figure;Revised figure 5.pdf

Figure 5.

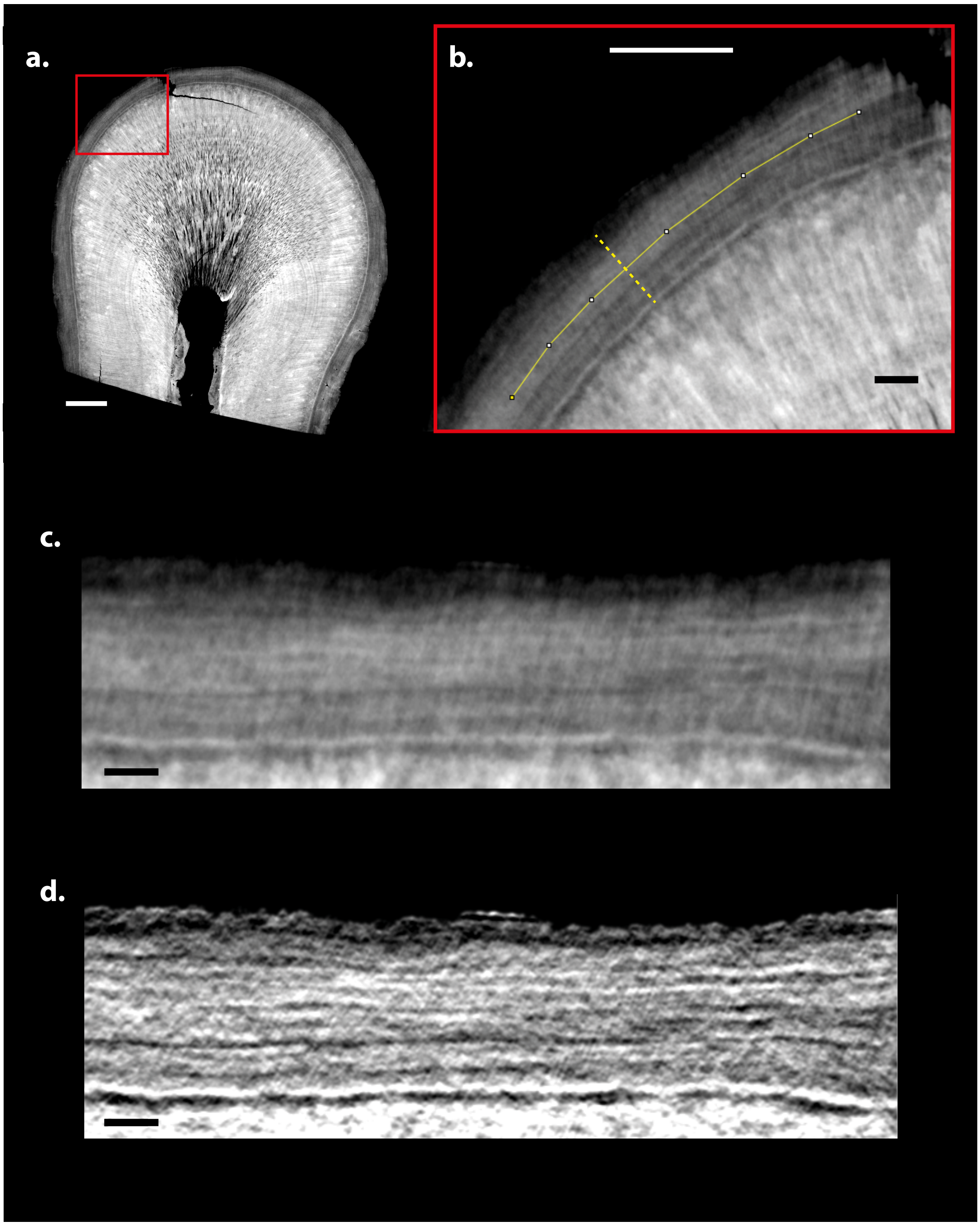

Figure

Click here to access/download;Figure;Revised figure 6.pdf

Figure 6.

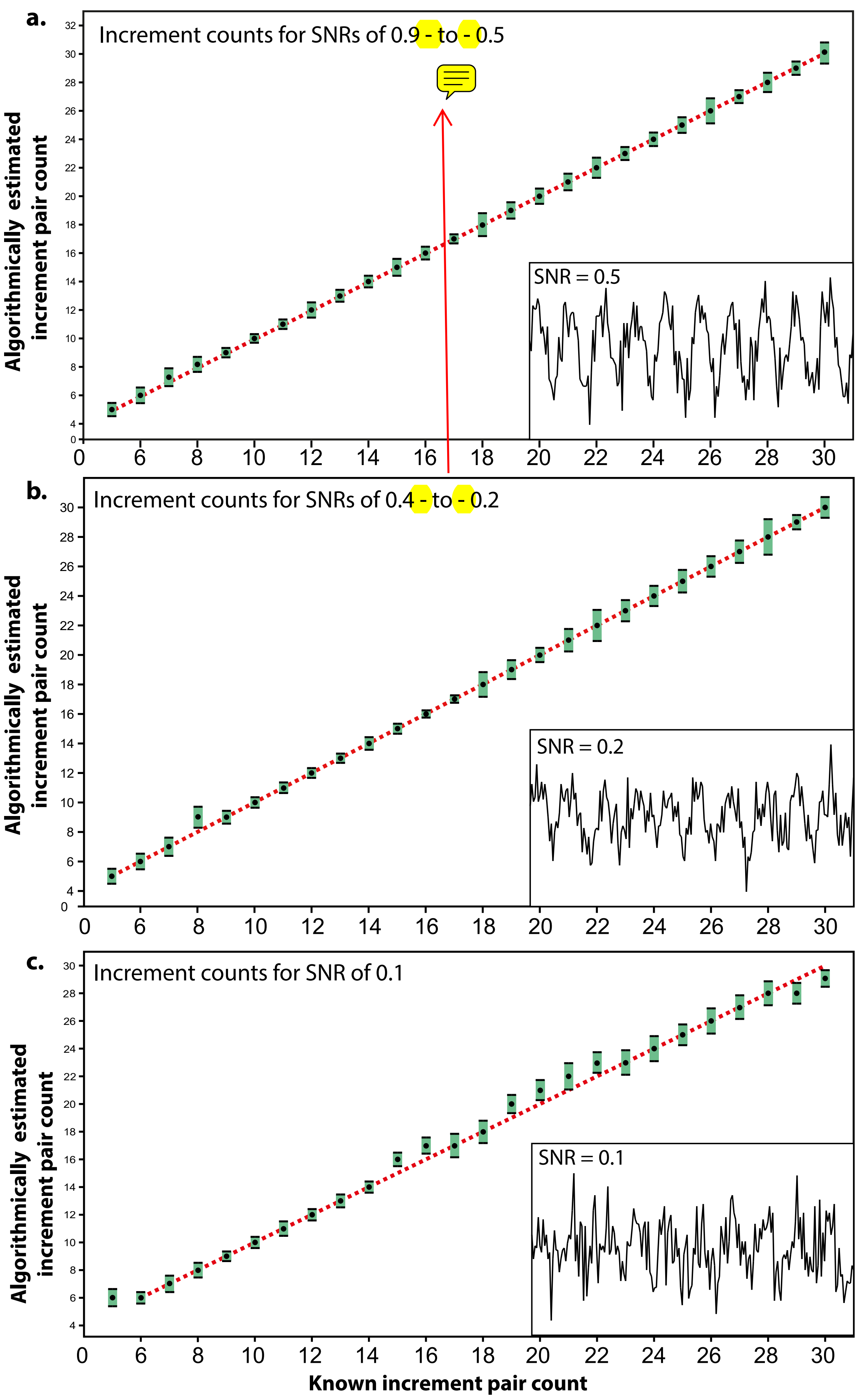

Figure
Click here to access/download;Figure;Revised figure 7 reduced (2).pdf

Figure 7.

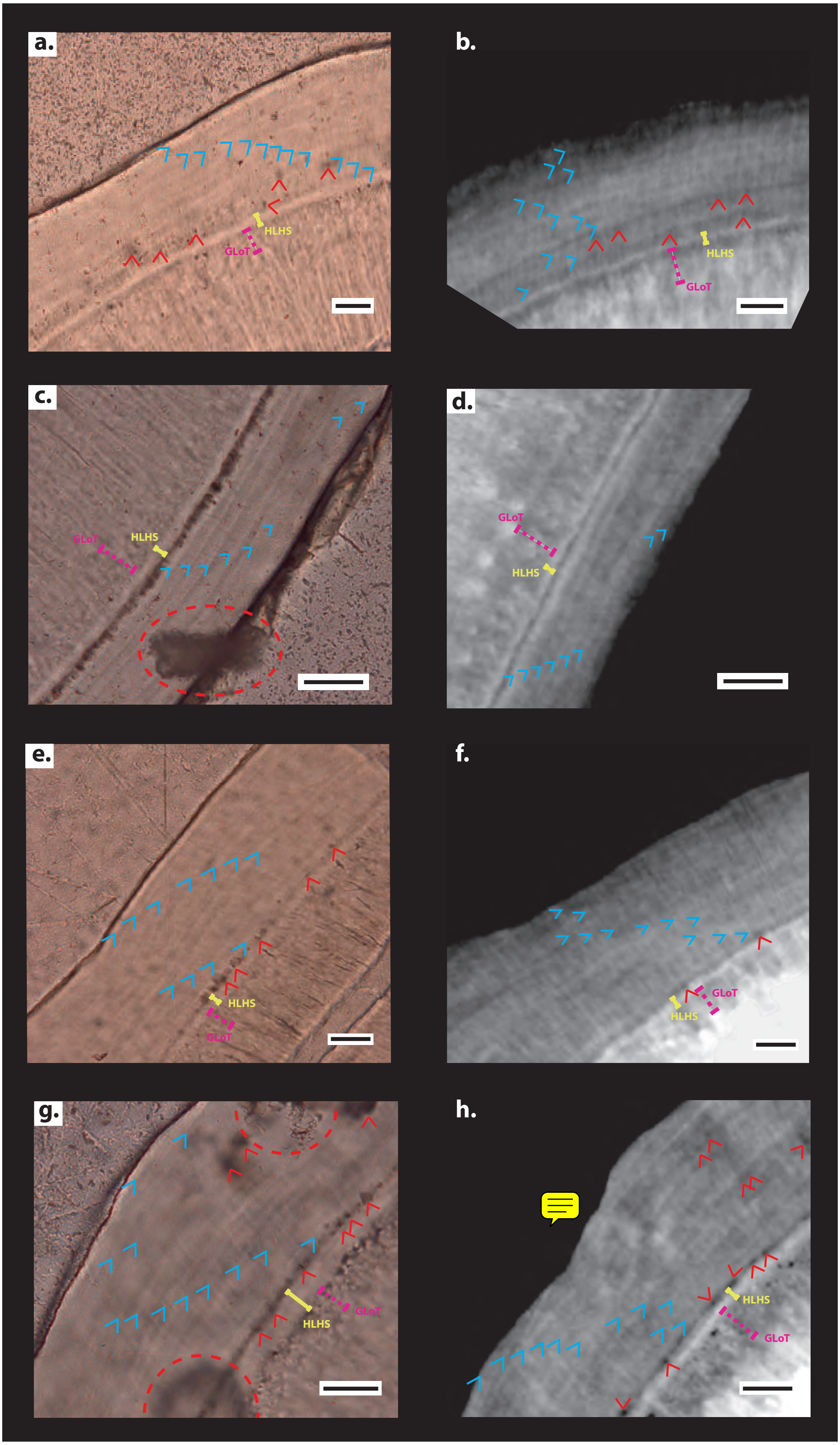

Figure 9.

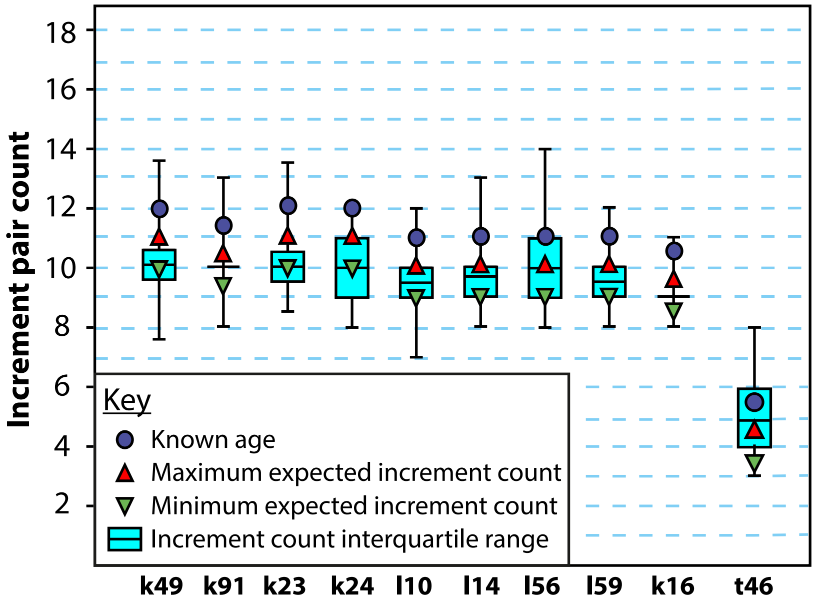
Increment pair count
18
16
14
12
10
8
6
4
2
Key
Known age
Maximum expected increment count
Minimum expected increment count
Increment count interquartile range
k49
k91
k23
k24
l10
l14
l56
l59
k16
t46

Click here to access/download
Supporting Information
Plos one Newham et al. 2021 supplement 22.07.docx

A robust, semi-automated approach for counting cementum increments imaged with X-ray computed tomography

Elis Newham[1,2*], Pamela G. Gill.[3,4], Kate Robson Brown[5,6], Neil J. Gostling[7], Ian J. Corfe[8,9], & Philipp Schneider[1,10*].

* Corresponding author

1. Bioengineering Science Research Group, Faculty of Engineering and Physical Sciences, University of Southampton. University Road, Southampton, SO17 1BJ, United Kingdom.
2. School of Engineering and Materials Science, Queen Mary University of London, London, E1 4NS, United Kingdom.
3. School of Earth Sciences, University of Bristol. Wills Memorial Building, Queens Road, Bristol, BS8 1RJ, United Kingdom.
4. Department of Earth Sciences, Natural History Museum. Cromwell Road, London, SW7 5BD, United Kingdom.
5. Department of Mechanical Engineering, Queen's Building, University of Bristol, Bristol, UK.
6. Department of Anthropology and Archaeology, University of Bristol. 43 Woodland Road, Bristol, BS8 1UU, United Kingdom.
7. School of Biological Sciences, Faculty of Environmental and Life Sciences, University of Southampton, Southampton, UK.
8. Developmental Biology Program, Institute of Biotechnology, University of Helsinki. Viikinkaari 5D, University of Helsinki, Helsinki, Finland.
9. Geological Survey of Finland, Espoo, Finland.

10. High-Performance Vision Systems, Center for Vision, Automation & Control, AIT Austrian Institute of Technology, Vienna, Austria.

Abstract

Cementum, the tissue attaching mammal tooth roots to the periodontal ligament, grows appositionally throughout life, displaying a series of circum-annual incremental features. These have been studied for decades as a direct record of chronological lifespan. The majority of previous studies on cementum have used traditional thin-section histological methods to image and analyse increments. However, several caveats have been raised in terms of studying cementum increments in thin-sections. Firstly, the limited number of thin-sections and the two-dimensional perspective they impart provide an incomplete interpretation of cementum structure, and studies often struggle or fail to overcome complications in increment patterns that complicate or inhibit increment counting. Increments have been repeatedly shown to both split and coalesce, creating accessory increments that can bias increment counts. Secondly, identification and counting of cementum increments using human vision is subjective, and it has led to inaccurate readings in several experiments studying individuals of known age. Here, we have attempted to optimise a recently introduced imaging modality for cementum imaging; X-ray propagation-based phase-contrast imaging (PPCI). X-ray PPCI was performed for a sample of rhesus macaque (*Macaca mulatta*) lower first molars (n=10) from a laboratory population of known age. A new method for semi-automatic increment counting was then integrated into a purpose-built software package for studying cementum increments. Qualitative comparison with data from conventional cementochronology, based on histological examination of tissue sections, confirmed that X-ray PPCI reliably non-destructively records cementum increments (given the appropriate preparation of specimens prior to imaging). Validation of the increment counting algorithm suggests that it is robust and provides accurate estimates of increment counts. In summary, we show that our new

increment counting method has the potential to overcome caveats of conventional cementochronology approaches, when used to analyse 3D images provided by X-ray PPCI.

1. Introduction

Mammalian teeth comprise three principal mineralised tissues: enamel, dentine and cementum. Each tissue provides its own record of growth via incremental patterns observed using thin-section histology that track periodic changes in their growth rates. Development of enamel and dentine is largely truncated after the maturation of the tooth. However, cementum grows continuously throughout life and provides a more complete record of an individual's life history [1]. Incremental features found in cementum are well understood to have a circum-annual periodicity, with one thick translucent increment, and one thin opaque increment formed every year (when viewed in thin-section under transmitted light microscopy; Fig. 1). These contrasting opacities are hypothesised to be primarily due to seasonal changes in the mineralisation rates of the cementum hydroxyapatite matrix. Cementum is composed of bundles of collagen fibres emanating from both the cementum itself (intrinsic fibres), or from the periodontal ligament or PDL (extrinsic 'Sharpey's' fibres), wrapped within a hydroxyapatite matrix [2]. Unfavourable growth seasons promote a reduction in the deposition rate of this matrix, while the mineralisation rate is unaffected [3]. This produces ultra-mineralisation of thinner portions of cementum during these periods ([1,4]; though see [3] and [5] for mineral distribution mapping studies that suggest higher mineral densities in wider, not narrower, cementum increments).

The proportion of collagen fibres emanating from either the cementum or the PDL defines the two major cementum tissue types. Acellular extrinsic fibre cementum (AEFC) contains predominantly Sharpey's fibres from the PDL. While the distribution of either tissue is not homogeneous, there is a general consensus that AEFC is concentrated in the crownward/coronal third of the root, while CIFC is the predominant tissue of the apical third of the root [6]. This tissue has the most regular periodicity and provides the most consistent growth record in the cementum. Cellular intrinsic fibre cementum (CIFC) contains only fibres originating from the cementum itself. This tissue grows more sporadically than AEFC with a less precise periodicity and is known to nucleate within regions of the tooth that undergo anomalously high occlusal forcing. As such, AEFC is usually the recommended cementum tissue for cementochronological studies and the tissue focussed on for this study ([6] and references within).

Several studies have questioned the accuracy, precision, and reliability of current methods of imaging and analysing cementum increments [7,8]. CIFC cementum is a dynamic, biomechanically responsive tissue and increments [illegible] known to both split and coalesce. These phenomena are also known to occure in AEFC ce[illegible]um, which can undermine confidence in their counts as an estimate of age at death. The overwhelming majority of previous studies have relied on thin-sectioning samples to image increments using light microscopy [6]. This allows increments to be viewed at high spatial resolutions, and offers a range of optical [9] and digital image processing [10,11] methods that filter and highlight increments to varying degrees. However, the destructive nature of thin-sectioning and the restrictive two-dimensional (2D) perspective offered by histological sections limit the understanding of the complex nature of cementum and its increments [7]. There is a wide range in the

reported accuracy (the proximity of estimated increment counts to known/true chronological age in years) and precision (repeatability and reproducibility between researchers) of increment counts [7,12,13], despite generally high reported accuracy and precision across multiple studies [6].

Computer vision and image processing have been explored to aid human counting of cementum increments, and to overcome the need for human counting itself [14,15]. Peaks and troughs in cementum opacity or greyscale 'luminance' can be extracted along radial transects through the cementum from digital micrographs of thin-sections using image processing software and studied by using 'Digital Cementum Luminance Analysis' (DCLA) [11] (Fig. 2a-b). Light increments are represented by distinct peaks in luminance values, and dark increments by distinct troughs in luminance values (Fig. 2b). These patterns are interpreted by either manually counting peaks and/or troughs, or through peak/trough detection algorithms (e.g. 'Find peaks' in ImageJ/Fiji; [16]). This abstraction of increments to peaks and troughs offers a less subjective method for manually counting increments, compared to directly reading thin-section images (Fig. 2b). Further, the use of numerical greyscale values to distinguish neighbouring light and dark increments allows quantitative thresholds to be defined for distinguishing increments. These thresholds represent a specific greyscale value from which peaks/troughs representing light/dark increments must differ from either the last trough or peak (respectively), or from the mean value of greyscale for the transect under study, in order to be identified as a 'genuine' increment.

However, DCLA methods have so far relied upon *a priori* assumptions regarding the contrast in greyscale values caused by incrementation versus those caused by other sources such as image noise. The chosen threshold value for

distinguishing increments in each DCLA method is specific to the image technique and hence image data in the original study, and so may not be robustly applied to data from other imaging modalities or techniques, or to taxa that fail to meet the specified threshold specified in the original study. Thus, the next stage in DCLA development should focus on developing a more flexible strategy for distinguishing cementum increments based on relative instead of absolute greyscale distribution criteria.

3D imaging, such as high-resolution computed tomography (CT) using X-ray propagation-based phase-contrast imaging (PPCI) at synchrotron radiation (SR) sources, may overcome the limitations posed by counting cementum increments in histological thin-sections. SR CT has revolutionised the study of other hard tissue microstructures such as vascular and cellular networks in bone [17:22] or growth increments in enamel [22-27]. SR CT imaging of such tissues provides a 3D context to the study of internal structures at sub-micrometre levels, and, with high signal-to-noise ratio (SNR) and high contrast-to-noise ratios (CNRs), provides high levels of image quality. PPCI through SR CT has recently allowed increments to be followed through the cementum of the teeth of archaeological humans [28-30] and macaque monkeys and early mammal fossils [31,32], overcoming the limitations of 2D thin-section-based imaging. The study of Le Cabec et al. [30] investigated a sample of human teeth from an archaeological population of known age at death, and reported high precision between counts performed by different observers, and for repeated counts performed by the same observer. However, although a strong correlation was found between age estimated by increment counts and known age, the accuracy (proximity of estimated age to known age) of estimates fell from 2.5 years in individuals 20-29 years old to 28 years in individuals 60-89 years old (with counts consistently underestimating true age). Due to the uniqueness of their archaeological

sample, the canines studied in Le Cabec et al. [30] could not be thin-sectioned. On this account, it could not be determined whether the nature of the source of this inaccuracy was biological, diagenetic (chemical changes to the cementum changing and overprinting original increments), or technical (insufficient image contrast between increments due to similar material properties in terms of X-ray interactions and/or due to unsuitable imaging and/or CT reconstruction settings). Further optimisation of cementum analysis using SR CT must be accompanied by comparison with histological data of the same regions of interest (ROIs), in order to relate image quality of SR CT data to the current "gold standard" for cementum imaging.

Here, we aim to optimise PPCI through SR CT for studying cementum increments in 3D, in comparison with the respective histological data in 2D. We will particularly assess the potential for overcoming the limitations identified in current approaches for counting cementum increments. As a second objective, we provide a validated, semi-automated and robust algorithm for user-independent counting of cementum increments.

2. Materials and Methods

2.1. Teeth samples

This study is focused on the analysis of the permanent right lower first molars (m_1) from a sample of 10 female Rhesus macaques (*Macaca mulatta*), raised under laboratory conditions and bred for biomedical research at the Primate Breeding Facility of Public Health England, Salisbury (UK) (Table 1). All animals used here were routinely monitored and checked for primate-borne diseases of risk to humans (including hepatitis B, herpes B and tuberculosis) and were humanely killed using an overdose of pentobarbital, under Home Office establishment licence 70-1707, due to

being unfit for breeding or whole-animal scientific procedural use. No animal was killed for the specific purpose of this study. Once the animals were killed, their lower jaws were mechanically dislocated and removed by Public Health England. Lower jaws were then freeze-stored at -20 °C prior to further tissue preparation. The studied sample was classed as Category B biological waste by the UK government. As no animal was sacrificed or harmed for the purpose of this study, no animal research ethics committee approval was needed. No permits were required for the described study, which complied with all relevant regulations under the UK Animals (Scientific Procedures) Act 1986 (ASPA).

To prepare specimens, lower jaws were first mechanically cleaned of soft tissue using surgical tools (scalpel, scissors and tweezers). The coronoid and angular processes were then removed using a handsaw. Once prepared, specimens were bathed in tap water in a sealed plastic container, which was stored in a fume cupboard for three weeks (21 days). This procedure was adopted to rot away the periodontal ligament and alveolar soft tissue that could not be mechanically removed. After three weeks, teeth were sufficiently loose within the jaw to be easily removed using surgical pliers. The left and right permanent m1 teeth of all animals were fixed in 10% paraformaldehyde (PFA) solution for 10 days to minimise risk of infection.

Finally, to minimise the X-ray absorption by dense mineralised tissue that was not of interest, the roots were mechanically separated before imaging. The crowns of all teeth were removed using a Buehler IsoMet® Low Speed precision sectioning saw equipped with an Acuthin^{tm} blade (Buehler Ltd, Lake Bluff, IL, USA). Using the same saw, the anterior and posterior roots of each m1 tooth were mechanically separated and mounted on 2 mm-thick carbon fibre rods (CR200600; Ripmax Ltd, Enfield, UK) cut to 1.5 cm length, using cyanoacrylate super glue. While this is a

destructive preparation technique (relative to Le Cabec et al. [30]), we determined it as necessary to provide the optimum sample geometry for SR CT imaging. Following PPCI experiments, transverse histological thin-sections of approximately [illegible] µm thickness were prepared, cut through the region of ROIs that were acquired by PPCI through SR CT, following the method outlined in Newham et al. [31].

Chronological age at death in years was known for each individual (Table 1). This data point, and the average age of eruption of lower permanent m1 teeth for captive populations of *Macaca mulatta* (approximately 12-18 months; [33]) provided an expected increment count for each individual (age at death minus average age at eruption). However, a potential variation of six months for m1 eruption necessitated the use of a minimum expected increment count, and a maximum expected increment count (one increment higher than the minimum expected count) (Table 1). All thin-sections and remaining whole tooth samples were reposited at the University of Southampton and are accessible upon request to EN and/or PS.

2.2. X-ray PPCI of cementum

PPCI for this study was performed during a three-day experiment at the TOMCAT beamline of the Swiss Light Source (SLS) (15-18 March 2016). The station at TOMCAT allows the user to control a series of key experimental settings, affecting the image quality of the resulting CT data [34,35]. The effects of these experimental settings must be systematically assessed in order to achieve optimal experimental conditions for the specific purpose and required image quality of a study.

In a preliminary experiment, the cementum tissue of specimen l56 was mounted using the convention described above, and imaged using X-ray PPCI through SR CT for a range of different experimental settings. Four key experimental settings (X-ray energy, exposure time, number of X-ray projections, and sample-to-

detector distance) were individually varied according to Table 2, while all other experimental settings were fixed at an X-ray energy of 20 keV, a voxel size of 0.66 µm and an exposure time of 150 ms for 1501 projections, at a sample-to-detector distance of 14.00 mm, which corresponds to a similar effective X-ray propagation distance of 13.99 mm [35] due to the parallel X-ray beam geometry at TOMCAT. The effects of changing experimental settings on image quality of cementum increments were characterised by the signal-to-noise ratio (SNR) and the contrast-to-noise ratio (CNR) as figure of merits. SNR quantifies the level of the image signal relative to the background noise. CNR is a useful measure for assessing image contrast between distinct structures, such as dark/light cementum increments.

Image quality measures for each experimental setting were calculated for 10 reconstructed µCT slices, representing the same regions of the tooth root of I56 in each scan (Fig. 3). SNR was calculated as the ratio between the mean greyscale value (representing density) for a 150-pixel × 150-pixel ROI of cementum ($\overline{g_c}$) and the standard deviation of a 150-pixel × 150-pixel sample of background (i.e. air) (σ_b) in each slice:

$$SNR = \frac{\overline{g_c}}{\sigma_b} \quad (1)$$

CNR was calculated as the difference of the mean greyscale values of the same ROIs of cementum ($\overline{g_c}$) and air ($\overline{g_b}$), respectively, divided by the pooled standard deviation, following the Pythagorean Theorem of Statistics ($Var(X \pm Y) = Var(X) + Var(Y)$ or $\sigma^2(X \pm Y) = \sigma^2(X) + \sigma^2(Y)$ for independent random variables X and Y, with Var and σ denoting the variance and standard deviation, respectively):

$$CNR = \frac{\overline{g_c} - \overline{g_b}}{\left[(\sigma_c^2 + \sigma_b^2)/2\right]^{1/2}} \quad (2)$$

where σ_c and σ_b represent the standard deviations of cementum and background, respectively. Mean values of SNR and CNR were calculated from the values for these 10 slices and compared between all experimental settings (Fig. 4 and Table 2).

Following this preliminary study, an X-ray energy of 20 keV and a sample-to-detector distance of 14 mm was chosen to scan three ROIs within the coronal third of the anterior root of each tooth, overlapping longitudinally and covering a volume of approximately 4X1.3X1.3 mm^3 (Table 1). This provided sufficient image contrast between cementum increments. For each scan, the exposure time was set to 150 ms, and 1501 projections were taken per scan. These settings provided sufficient image quality at scan times that allowed the entire sample of teeth to be imaged during our fixed-time experiment. The phase of each X-ray projection was retrieved through the Paganin single-distance non-iterative phase retrieval algorithm, [36], implemented in-house at TOMCAT. The values of the imaginary part of the refractive index δ=3.7 10^{-8} and the decrement of the real part of the refractive index β=1.7 10^{-10}, and hence the ratio $\delta/\beta = 218$, were fixed for all scans. Phase contrast is much more sensitive for imaging low absorbency materials with small differences in material properties, compared to traditional X-ray absorption [37]. The retrieved phase images were reconstructed using an in-house implementation of the Gridrec algorithm [38] at TOMCAT. The resulting CT reconstructions were saved as 16-bit tiff stacks.

2.3. Image processing: straightening and filtering

Image processing of the “raw” reconstruction data is often needed before digital image visualisation, segmentation and quantification. This can involve a wide range of image processing methods, the majority of which are based on the manipulation of 2D pixels and/or 3D voxels using mathematical operations [39]. Here, we applied two principal image processing methods to individual transverse CT slices: straightening

and isolation of cementum, and directional filtering of cementum increments. Straightening and isolation involved distinguishing the cementum from the dentine tissue that it surrounds in each slice, removing the dentine data and algorithmically minimising/”straightening” the remaining cementum data along its circumferential axis (Fig. 5). This then allowed directional filtering to enhance image contrast in a single direction between straightened cementum increments.

For circumferential structures such as cementum increments (when viewed in CT slices transversely through the tooth root), it is often difficult to apply standard image processing tools and analyses without distorting results, due to complexities in their patterns and boundaries. Hence, 2D straightening algorithms are often applied in order to further analyse the data. We chose to use the ‘Straighten’ tool of the open source ImageJ/Fiji image analysis software [16]. This tool applies cubic-spline interpolation across a segmented midline of the feature of interest, which is defined by the user. Straightening is then performed using a series of non-linear cubic splines for an arbitrary number of pixels on either side of the midline that can also be determined by the user. Here, we assigned this number on an individual basis for each dataset, based on the (radial) thickness of cementum being imaged in the first/bottom-most slice, to ensure that all cementum, but no dentine, was included in the processed image (Fig. 5.a-b). This workflow was then repeated in a semi-automated fashion for all CT slices of each dataset following Newham et al. [31], wherein the same midline coordinates and thickness of the segmentation was applied for as many slices as possible from the first slice of a dataset. Due to the change in shape of the root and non-perfect alignment of the sample axis and scanning axis, these midline coordinates had to be adjusted several times through each scan, as the sample changed in size and drifted through the volume. This required manual inspection of the amount of

cementum captured by the segmentation through the volume from the first slice, and adjusting the thickness and midline coordinates.

Following straightening, cementum datasets were further processed using directional filtering in order to enhance contrast between increments (see Supplement, section 1. ‘Image processing by directional filtering’) (Fig. 5.d). Filtering is commonly used to suppress the contribution of unwanted signals such as noise, while preserving and enhancing the targeted signal [illegible]nage contributions for the analysis in question (Freeman and Adelson, 1991). We used a custom MATLAB (R2016a; The MathWorks, Inc., Natick, MA, USA) tool called ‘SteerGauss’ (version 1.0.0.0) developed and made freely available by Lanman [40], in order to employ directional Gaussian filtering of straightened cementum images following Freeman and Adelson [41]. For directional Gaussian filtering, a Gaussian function for a set of 2D (x,y) Cartesian coordinates can be prescribed for any arbitrary orientation using a directional derivative operator that interpolates between two ‘basic’ Gaussian functions, directed at 0° and 90°, respectively. Straightened increments follow similar longitudinal paths, so a steerable filter can be used to select a single orientation of all increments in an image (Fig. 5.c-d). For the current study, the use of a directional Gaussian filter oriented at 90° to the x-axis has been shown to substantially enhance image contrast between straightened cementum increments (see Supplement, section 1. ‘Image processing by directional filtering’) (Fig. 5).

2.4. Cementum increment counting algorithm

The cementum increment counting algorithm developed here was designed to count cementum increments in a user-independent and semi-automated fashion, further developing the rationale proposed by DCLA for distinguishing individual increments

by using a cut-off point that greyscale peaks/troughs must differ by, in order to be counted as a genuine increment. Our algorithm employs population statistics (mean and standard deviation of greyscale values) to count increments, based on the unique distribution of greyscale values within each individual CT slice (see Supplement, section 2 ‘Robustness testing for increment counting algorithm’). As in DCLA, this method makes use of the average greyscale values along 10 pixel-thick transects through cementum (Fig. 2a). Adapting methods used in tribological surface profiling [42,43] , individual transects are separated into five sections of equal length (Fig. 2b) (see Supplement, section 3 ‘Splitting of transects through the cementum’). PPCI SR CT datasets of cementum show an overarching reduction in greyscale (density) values from the cemento-dentine boundary to the outer-most cementum increment (Fig. 2a-b) [31,32]. Therefore, the use of the mean greyscale value and its standard deviation for the entire transect, as opposed to local values for individual sections of the transect, may preclude the counting of genuine increments towards the outermost increment (as greyscale peaks/troughs are below the mean value), and counting of increments towards the cemento-dentine boundary (as greyscale peaks/troughs are above the mean value). The mean and standard deviation of greyscale values in each section is then calculated (Fig. [illegible]d), and light-dark increment pairs are distinguished as peak-trough systems in greyscale that depart from the mean value beyond the local standard deviation in each section (Fig. 2c).

Most importantly, this new method for increment counting can be operated in a semi-automated fashion, following an algorithm implemented in the MATLAB statistical environment (see Appendix for MATLAB script). In MATLAB, each individual straightened and filtered cementum image is investigated along a series of 1000 transects through the cementum chosen at random using a random number

generator (Fig. 2a). For each transect, the distance across the transect that the first pixel above zero appears is saved, which gives the radial length of sampled cementum along the transect. Any transect that is less than the lower standard deviation of the saved lengths is then deselected and resampled until all transects fulfil the lower standard deviation of the original sample. Each transect is divided into five sections of equal length (Fig. 2b), and a cubic spline ('Smoothing spline' function in MATLAB) is fitted to the greyscale pattern captured within each section, in order to minimise the influence of image noise on peak/trough patterns [44] (Fig. 2b). For these five smoothed datasets, their mean greyscale value (red lines in Figure 2) is calculated. An upper 'cut-off' value (green lines in Figure 2) is then determined for each section as its mean greyscale value plus half the standard deviation of its greyscale values, and lower 'cut-off' value (blue lines in Figure 2) as the mean greyscale value minus half of the standard deviation. Two new datasets are then created for each section, the first comprised of only greyscale values above the mean, and the other of values below the mean (Fig. 2c). The dataset comprising higher greyscale values thus consists solely of greyscale 'peaks' (local apex in greyscale values), while the dataset comprising lower greyscale values consists solely of greyscale 'troughs' (local nadir in greyscale values) (Fig. 2c). The 'Findpeaks' tool (part of the default 'Signal processing toolbox' in MATLAB) is then used to identify peaks and troughs in their respective datasets (following multiplication with -1 to convert troughs into peaks) and calculate their difference from the mean greyscale value of that section. This allows peaks and troughs that extend beyond the top and bottom cut-off values (respectively) for each section to be identified, providing the first stage of estimating increment and increment pair counts (Fig. 2b-d).

Following this first estimate of increment and increment pair counts, further steps are taken within the algorithm to ensure that ‘piggy-back’ features (secondary peaks/troughs along the ascending/descending limbs of genuine increment peaks and troughs, formed either due to accessory increments from increment splitting/coalescence, or image noise) do not affect increment counts (Fig. 2). No peaks are counted that immediately proceed from the last respective peak; so only one peak is counted for every trough (in Fig. 2c. peaks i and ii are not counted). Also, no peak/trough system along the transect for which each feature is separated by less than three pixels along the transect (or 1.98 µm), are counted, to ensure that grey scale variations on a small scale do not influence increment counts (Fig. 2c peaks i and ii). While this procedure does not necessarily target or discriminate “accessory” or secondary increments from “real” circum-annual increments, it remains an objective way of discerning “real” increments that does not rely on subjective interpretation by the user. This in-turn relies on user-experience, which is one of the primary elements of cementochronology we are here seeking to minimise.

A final measure is taken to account for increments that are only partly captured inside a neighbouring set of sections along one transect (Fig. 2b-c.). As only the ascending/descending limb of such features would be captured in each section, they may not be detected as a peak/trough in greyscale in either section using the first stage of the increment counting algorithm, which defines peaks or troughs with reference to the two troughs or peaks surrounding them respectively. A second step is therefore undertaken to distinguish, measure and count these features based on their greyscale values relative to the upper and lower standard deviations of each neighbouring section (described in Supplement Section 4 ‘Accounting for

increments split between two neighboring sections'). Once increment pair counts are estimated for the 1000 random transects, the mean and standard deviation are calculated, providing a final estimate of cementum increment pair count.

The robustness of the proposed algorithm was tested by applying it to a series of digital sine wave patterns of known increment number between five and 30. Random noise at different degrees was applied to these patterns in a controlled manner by increasing their standard deviation along the *y*-axis (Fig. 6). Noise was increased incrementally by SNR decrements of 0.1; starting from a SNR of 0.9 and ending at an SNR of 0.1. For each SNR level, increments were counted for 30 sine wave patterns for each count between five and 30. Increment estimates were considered as accurate if the mean estimated count equalled the known increment number to an accuracy of $\pm$ 0.5. Estimates were considered robust for each count as long as the standard deviation for the 30 counted sine wave patterns was < 1, as values above this may produce estimated increment counts of over 1 year above/below known/expected counts (see Supplement Section 2 'Robustness testing for increment counting algorithm').

2.7. Application of cementum increment counting algorithm

The increment counting algorithm presented here was used to generate estimates of increment counts for straightened and filtered CT slices for each lower first molar specimen of the 10 *Macaca mulatta* individuals. We applied the cementum increment counting algorithm to 30 CT slices for each individual, representative of highest cementum increment contrast and quality for each individual [6]. Each straightened and filtered CT dataset was examined by eye, in order to find the regions of highest increment contrast and minimum amounts of complexity in increment patterns (i.e. splitting and coalescence of increments). Slices were

selected contiguously through these regions until a number of 30 slices had been reached. 1000 transects were plotted through the cementum in each CT slice, and increment pair counts were generated for each transect. The mean increment pair count for all 1000 transects was then used as the estimated increment pair count for the slice, and the mean count of the 30 slices rounded to the nearest integer was defined as the estimated increment pair count for that *Macaca mulatta* individual.

3. Results

3.1. Optimisation of cementum imaging

When the X-ray energy was changed in isolation, SNR became consistently lower with increasing X-ray energy, whereas CNR peaked at 20 keV, before steadily falling with increasing X-ray energy beyond this point (Fig. 4a). SNR and CNR steadily improved with increasing exposure time (Fig. 4b). SNR and CNR also improved with increased number of projections, although the relative increase in CNR was marginal between 3001 and 4501 projections (Fig. 4c). SNR steadily increased with larger sample-to-detector distances up to 60 mm (Fig. 4d). Whereas CNR steadily rose from 14 mm sample-to-detector distance to a peak at 28 mm, it fell between a sample-to-detector distance of 28 mm and 100 mm (Fig. 4d).

The image quality of the dataset imaged at 28 mm sample-to-detector distance (Fig. 3c) represents an optimum in the trade-off between spatial resolution and contrast for our application. The smoothing inherent in the Paganin phase retrieval algorithm [35] flattens out increment boundaries and reduces noise, while (mean) greyscale differences are retained between light and dark increments to an extent that offers sufficient image contrast to identify individual cementum increments. For datasets created using smaller sample-to-detector distances (14 mm - 20 mm), high

image contrast resulted between increments, but their boundaries were smoothed and less well defined (case Fig. 3b). For sample-to-detector distances above this, the increasing amounts of smoothing diminished the differences in greyscale values between light and dark increments such that by 100 mm sample-to-detector distance, they were difficult to distinguish by eye (case Fig. 3d). This can also be shown quantitatively by plotting greyscale values along transects through the same region of cementum in each dataset (Fig. 3e) acquired at different sample-to-detector distances.

3.2. Cementum imaging results

Cementum was clearly visible in each CT dataset as an incremental tissue wrapping around the dentine of tooth roots and comprising a series of radial increments (Figs. 5 and 7-8). The cementum could be distinguished from the dentine due to its significantly lower mean grey values, and the cemento-dentine boundary was marked by the characteristic tissues of the granular layer of Tomes and the high-density hyaline layer of Hopewell Smith (Fig. 7). Individual increments were clearly visible within the cementum and could be followed through the entire dataset, both transversely and longitudinally (Fig. 8).

Comparison between CT slices and histological thin-sections of the same regions of cementum (created using the method outlined in Newham et al. [31] and imaged using the method outlined in Supplement Section 5 ‘Thin-section Imaging’) suggests that both imaging techniques represent the same cementum increments (Fig. 7; Supplement Fig. S4). Optical differences between increments in histological data were reflected as grey value differences in CT data. Thick, light increments in histological data corresponded to thick, light increments in CT data, and so absorbed a higher proportion of X-rays relative to thin, dark increments (Fig. 7). Volumetric CT data could further be used to help elucidat[illegible]imary increments from accessory

increments in several specimens (Fig. 8). Complexities in increment patterns were witnessed intermittently in every *Macaca mulatta* individual, with individual increments splitting and coalescing to create apparent accessory increments. Following Newham et al. [31,32], individual increments could be mapped through the cementum tissue, and the same primary increments could be plotted through the entire scanned tissue volume (approximately 4 mm^3) (Fig. 8) across these complexities, and distinguished from the accessory increments created. Therefore, regions that were confounded by splitting and coalescing of these increments could be distinguished and excluded for analysis of increment counts (Fig. 8). Also, CIFC, the tissue with the least chronological precision in its increment periodicity [6], could be distinguished from acellular cementum by the presence of cellular voids, and so could be avoided when identifying high-contrast regions of increments with a circum-annual periodicity (Fig. 7). These two factors, aided by the entire coronal (crownward) third of the cementum tissue being imaged, led to the identification of the highest quality regions of circum-annual cementum increments for each specimen (Fig. 7).

3.3. Validation of cementum increment counting algorithm

Robustness tests for the proposed increment counting algorithm suggest that it is reliable for SNRs down to 0.2 (Fig. 6). For each simulated pattern of known increment number, the average value of 30 automated counts was identical to the known count for SNRs between 0.9-0.5. The upper/lower standard deviations of these samples did not exceed one integer above/below the known count (Fig. 6). Between SNR levels of 0.5-0.2, average automated counts only differed from known increment number by a value of one in a single sample (with a known increment number of eight). The standard deviations of automated counts exceed one integer above/below the known count for known increment counts of 22 and 28 (Fig. 6). SNRs of 0.1

introduced more errors of between one and two in increment count when compared to the known increment number, and the automated count was outside the region of one standard deviations around the known increment number (Fig. 6).

When increments were algorithmically counted in our macaque data and compared to expected counts for our sample based on known age, a Spearman's *r* of 0.77 (p<0.009) and Kendall's τ of 0.71 (p = 0.004) suggest significant correlation between semi-automated increment pair counts and expected numbers of cementum increment pairs. The mean of the semi-automated increment pair counts for each *Macaca mulatta* individual either met the minimum or maximum expected count based on their known age or fell in-between the two for every sample, apart from the juvenile individual t46 whose mean estimated count 0.5 years more than the maximum expected count (Table 1 and Fig. 9). Juvenile cementum has been previously shown to contain more complex incrementation and greater amounts of increment splitting and coalescence than adult cementum [45]. Standard deviations of increment pair counts (average = 0.83) for the 30 individual CT slices examined for each individual did not exceed one for any *Macaca mulatta* individual. This suggests a precision of within one year for estimated increment counts using the proposed cementum increment counting algorithm.

4. Discussion

4.1. Image quality of SR CT data and optimisation of cementum imaging

The positive relationship observed between both SNR and CNR with increasing exposure time and number of projections has been expected following SR CT imaging of other hard tissues [24, 46]. The opposite relationship seen between SNR and X-ray energy can also be explained by a diminished X-ray absorption with

increased X-ray energy due to an exponentially decreased probability of photoelectric interactions between X-rays and the tissue. CNR has a more complex relationship with each experimental setting, with an optimum setting at a different level compared to SNR for each experimental setting. For instance, we located the optimal energy at TOMCAT for cementum increments in terms of CNR at around 20-21 keV, while SNR was highest at 19 KeV and continuously decreased with higher X-ray energies.

The steady increase in SNR with increasing sample-to-detector distance is in agreement with the results of Kitchen et al. [34], but in contrast to the results of Zeller-Plumhoff et al. [35]. Instead, Zeller-Plumhoff et al. [35] found that SNR of PPCI SR CT data of muscle tissue steadily decreased when sample-to-detector distance was increased between 30 mm and 60 mm at TOMCAT. The main factors responsible for the increase in SNR with increasing sample-to-detector distance in our study are the steady decrease in the standard deviation of the image background (σ_b) with increasing sample-to-detector distance versus the peak in mean greyscale value of cementum ($\overline{g_c}$) between 28-60 mm (Fig. 4e). The Paganin phase retrieval algorithm acts as a low pass filter, reducing the image noise in the resultant CT reconstructions. This filtering has been enhanced here with increased sample-to-detector and hence propagation distance, as shown by Kitchen et al. [34]. The reason for the different patterns encountered in SNR between the results of Kitchen et al. [34] and those of Zeller-Plumhoff et al. [35] were attributed by Zeller-Plumhoff et al. [35] to be due to different targets in terms of image quality, when considering the optimal ratio of δ/β for the Paganin phase retrieval algorithm. The objective of Kitchen et al. [34], and of our study, was primarily to enhance image contrast within the PPCI SR CT data, whereas Zeller-Plumhoff et al. [35] also considered the sharpness of feature boundaries when optimising δ/β. Moreover, the material

properties of cementum are different to the soft tissues studied by both Kitchen et al. [34] (lung tissue) and Zeller-Plumhoff et al. [35] (muscle tissue).

It is important to remember that, while our optimisation study was focused on image quality, other factors play important roles for the PPCI of cementum. Firstly, cementum has recently been suggested as an important reservoir of ancient DNA (aDNA) in sub-fossil and archaeological specimens, including early hominins such as Neanderthals [47]. It is also well understood that the significant X-ray doses provided by low energy and/or sub-micrometre resolution PPCI is sufficient to denature and destroy aDNA within the scanned ROI [47]. Hence, the optimum experimental settings presented here may well involve an X-ray dose that is too high to be viable for specimens bound for aDNA extraction and study [30].

Further, while increasing the number of angular projections increases image quality, it also increases the total acquisition time of each CT scan. As each synchrotron experiment is awarded a restricted amount of beam time, optimal experimental setings have to be weighed up against scanning time to image a sufficient sample of specimens. Hence, given the time allocated for our experiment, we retained 1501 projections per CT scan, half of the optimal value around 3000-4000 in terms of SNR (Fig. 4), in order to image our complete sample within the allotted beamtime.

4.2. X-ray PPCI versus thin-section imaging for counting cementum increments

The first objective of this study was to optimise PPCI through SR CT for studying cementum increments in 3D tomographic data, as an alternative strategy to destructive thin-sectioning and light microscopy for imaging and counting cementum increments. We have shown here that optimised PPCI strategies can overcome the principal caveats of thin-section imaging: namely the destructive sample preparation

process (with the caveat that we isolate both tooth roots from the crown) and the limited 2D view of tissue that is actually 3D in nature, so lacking context for interpreting complexities in increment patterns; and also limited control over which cementum tissue type is imaged (AEFC versus CIFC). The high image quality offered by SR CT, including phase retrieval offered in PPCI, has provided comparable fidelity for counting individual cementum increments to thin-section histological images of the same regions. This is of particular note given that the study sample originates from a captive population with minimal seasonal environmental and nutritional variation. It is understood that the cementum of captive mammals presents lower increment contrast than free-roaming populations [1], and so the identification and accurate counting of circum-annual increments of PPCI data in captive cementum may be another promising element of this technique. This also adds to the body of work finding circum-annual increments in modern humans ([6] and references therein), suggesting that incrementation follows an endogenous (potentially hormone-driven; see Newham et al. [30]) rhythm as opposed to primarily reflecting seasonal dietary differences [4].

The volumetric nature of CT datasets allows navigation through the entire cementum tissue at an isotropic and sub-micrometre nominal spatial resolution. Individual cementum increments can be followed across regions exhibiting complex cementum patterns, created by splitting and coalescence of increments, and regions of CIFC can be avoided when analysing AEFC. This minimises the potential for inaccurate increment counting. As a non-destructive technique the use of PPCI through SR CT for cementochronology permits the study of cementum in specimens previously beyond the reach of traditional histological analyses that are destructive, including fossils [32] and archaeological specimens [28-30]. As there is no physical

thin-sectioning of the tissue involved for CT, images are not affected by tissue preparation artefacts such as scratches on the ground and polished thin section or tissue distortion through the mechanical cutting process, which can obscure or alter image details on cementum increments [6,14].

However, our PPCI of cementum through SR CT has also highlighted the sensitivity of cementum image quality to experimental settings. This, alongside other factors described in Section 4.1., suggests that optimisation of experimental settings should be conducted preliminary to every cementochronological PPCI experiment using SR CT, in order to ensure optimised image quality for identifying and counting cementum increments within the constraints given by the broader aims of the particular experiment [30,47]. Optimal experimental settings are specific to the optics of the synchrotron beamline and the material properties, size and morphology of the specimen, and so should be investigated when any of these factors are changed.

It also became apparent during scanning that micrometre-scale cracks, which are not visible macroscopically, have formed within the cementum tissue (Supplementary Fig. S5), most probably due the interaction of the hard X-rays with residual water left within the teeth and/or related effects due to this interaction [47]. Although this damage could not be seen macroscopically, comparison with the lack of observations of any damage in archaeological [30] and fossil [32] teeth confirms that the preparation of “fresh” teeth was suboptimal for SR CT scanning, as residual water was retained. Further, it cannot be confirmed whether micro-cracks occurred solely during the scanning process, or started due to demineralisation during fixation in paraformaldehyde. This should be examined in future studies via microscopic examination of the cementum of fixed teeth. Moreover, further optimisation of the

preparation procedure for fresh teeth is still needed, that ensures both minimal risk of biological contamination and minimal damage to the tissue during CT scanning.

4.3.Cementum increment counting algorithm

The second objective of this study was to provide a validated, semi-automated and robust algorithm for user-independent counting of cementum increments. The manual counting of cementum increments amongst a restricted number of thin-sections per tooth, plays a central role in the current user-dependent approach for counting cementum increments. This subjectivity has led to a wide range of different accuracies and precisions reported for increment counts and their correlation with known age in animal and human samples. Both accuracy and precision in estimated increment counts correlate with the experience of the researcher when compared to known age (i.e. expected increment count) in validation studies [6]. Our algorithm offers a new method for objectively counting cementum increments in a user-independent and semi-automated fashion. This substantially decreases the subjectivity and propensity for human error involved in increment counting (see Supplement section 6. 'Testing the effects of variability in straightening on increment counting'). Within the same selected sample of straightened, isolated and filtered PPCI slices, our algorithm requires no further human input for counting cementum increments and will estimate the same increment count regardless of the experience of the researcher. The accuracy and precision of this algorithm has been validated here for both simulated data and our experimental sample of *Macaca mulatta* cementum (in both 8-bit and 16-bit data; see Supplement Section 1). It could also be further assessed in the same quantitative manner with other PPCI cementum data from animals of known age. Such assessment will allow for further optimisation of our algorithm and tailoring for a wide range of PPCI cementum data.

Finally, although we state the advantages of PPCI imaging over traditional thin-section histological imaging here, the validation of our application on thin-section data of cementum from animals of known age may afford its application for thin-section images. If found to be an accurate method for counting thin-section increments, implementation of our algorithm for thin-sections has potential as an important tool for validating the accuracy of counts estimated by-eye, or even discounting the need for counting increments by-eye completely.

5. Conclusion

In conclusion, we have undertaken a first systematic experimental study on cementum increment counting for non-fossilised dental tissue, based on a comparison between optimised PPCI through SR CT and thin-section histological imaging. Comparison between these two imaging techniques has shown that PPCI SR CT data can provide sufficient spatial resolution and image contrast to reproduce individual growth increments in the cementum tissue (as well as accessory increments that can be followed through their own trajectory and discounted, thanks to the 3D nature of CT data). CT reconstructions are of sufficient quality to count increments semi-automatically using image processing, by defining them as peaks and troughs in greyscale values along transects through the cementum. We have implemented this semi-automated method of increment counting as part of a novel workflow of image processing (cementum isolation, straightening and filtering) and analysis (application of a purpose-built increment counting algorithm). This may help future studies to overcome the central caveat facing current studies of cementum increments: the subjectivity inherent in counting increments by eye that depends on the individual researcher. The combination of non-destructive imaging and objective increment

counting may open up a new range of specimens, samples and studies not suitable for destructive thin-section analysis, and help to exploit the potential of cementum as a record of life history for archaeology, anthropology, forensic science and palaeontology.

Acknowledgments and funding: This study was part-funded by a Natural Environmental Research Council/Engineering and Physical Sciences Research Council doctoral candidateship (UK; grant number NE/R009783/1). Funding was also provided by Ginko Investments Ltd (Bristol, UK) for purchasing external hard drives and funding travel to the Swiss Light Source, and the Academy of Finland. We acknowledge the Paul Scherrer Institute, Villigen, Switzerland for provision of synchrotron radiation beamtime at the TOMCAT beamline of the SLS (Experiment 20151391) and would like to thank Iwan Jerjen, Mark Mavrogordato, Orestis Katsamenis, Sharif Ahmed, Christianne Fernee Juan Núñez, and Priscilla Bayle for their assistance during our beamtime.

Conflict of interest. The authors report no conflicts of interest. The funders had no role in study design, data collection and analysis, decision to publish, or preparation of the manuscript. The private funding received from Ginko Investments Ltd does not alter our adherence to PLOS ONE policies on sharing data and materials.

Author contributions. EN designed, validated and performed all analyses. All authors were involved in writing the proposal for synchrotron beamtime and synchrotron imaging. All authors contributed to drafting and revising the manuscript.

Data accessibility. All data supporting this study are openly available from the University of Southampton repository (“https://doi.org/10.5258/SOTON/D1722).

6. References

1. Klevezal G. Recording structures of mammals. Boca Raton, Florida: CRC Press; 1996.
2. Foster BL. On the discovery of cementum. J. Periodontal Res. 2017;54:666-685.
3. Stock SR, Finney LA, Telser A, Maxey E, Vogt S, Okasinski JS. Cementum structure in Beluga whale teeth. Acta biomater. 2017;4:289-299.
4. Lieberman DE. Life history variables preserved in dental cementum microstructure. Science. 1993;261(5125):1162-1164.
5. Dean C, Le Cabec A, Spiers K, Zhang Y, Garrevot J. Incremental distribution of strontium and zinc in great ape and fossil hominin cementum using synchrotron X-ray fluorescence mapping. J. R. Soc. Interface. 2018;15(138):20170626.
6. Naji S, Colard T, Blondiaux J, Bertrand B, d'Incau E, Bocquet-Appel JP. Cementochronology, to cut or not to cut? Int. J. Paleopathol. 2016;15:113-119.
7. Renz H, Radlanski RJ. Incremental lines in root cementum of human teeth – a reliable age marker? Homo. 2006;57(1):29-50.
8. Kasetty S, Rammanohar M, Raju Ragavendra T. Dental cementum in age estimation: a polarized light and stereomicroscopic study. J. Forensic Sci. 2010;55(3): 779-783.
9. Stutz AJ. Polarizing microscopy identification of chemical diagenesis in archaeological cementum. J. Archaeol. Sci. 2002;29(11):1327-1347.
10. Lieberman DE, Deacon TW, Meadow RH. Computer image enhancement and analysis of cementum increments as applied to teeth of *Gazella gazella*. J. Archaeol. Sci. 1990;17(5):519-533.

11. Wall- Scheffler CM, Foley RA. Digital cementum luminance analysis (DCLA): a tool for the analysis of climatic and seasonal signals in dental cementum. Int. J. Osteoarchaeol. 2008;18(1):11-27.
12. Lipsinic FE, Paunovich E, Houston GD, Robison SF. Correlation of age and incremental lines in the cementum of human teeth. J. Forensic Sci. 1986;31(3):982-989.
13. Obertová Z, Francken M. Tooth cementum annulation method: accuracy and applicability. In Koppe T, Meyer G, Alt KW, Brook A, Dean MC, Kjaer I, et al. editors. Comparative dental morphology Vol. 13. Basel, Switzerland; 2009. p. 184-189.
14. Czermak A, Czermak A, Ernst H, Grupe G. A new method for the automated age-at-death evaluation by tooth-cementum annulation (TCA). Anthropol. Anz. 2006;64(1):25-40.
15. Klauenberg K, Lagona, F. Hidden Markov random field models for TCA image analysis. Computational Statistics & Data Analysis. 2007;52(2):855-868.
16. Schneider CA, Rasband WS, Eliceiri KW. NIH Image to ImageJ: 25 years of image analysis. Nat. Methods. 2012;9(7):671.
17. Schneider P, Stauber M, Voide R, Stampanoni M, Donahue R, Müller R. Ultrastructural properties in cortical bone vary greatly in two inbred strains of mice as assessed by synchrotron light based micro- and nano- CT. J. Bone Miner. Res. 2007;22(10):1557-1570.
18. Schneider P, Krucker T, Meyer E, Ulmann- Schuler A. Weber B, Stampanoni M, et al. Simultaneous 3D visualization and quantification of murine bone and bone vasculature using micro- computed tomography and vascular replica. Microsc. Res. Tech. 2009;72(9):690-701.

19. Sanchez S, Ahlberg PE, Trinajstic KM, Mirone A, Tafforeau P. Three-dimensional synchrotron virtual paleohistology: a new insight into the world of fossil bone microstructures. Microsc. Microanal. 2012;18(5):1095-1105.

20. Goggin PM, Zygalakis KC, Oreffo RO, Schneider P. High-resolution 3D imaging of osteocytes and computational modelling in mechanobiology: insights on bone development, ageing, health and disease. Eur. Cells and Mater. 2016;31:264-95.

21. Núñez JA, Goring A, Hesse E, Thurner PJ, Schneider P, Clarkin CE. Simultaneous visualisation of calcified bone microstructure and intracortical vasculature using synchrotron X-ray phase contrast-enhanced tomography. Sci. Rep. 2017;7(1):13289.

22. Goring A, Sharma A, Javaheri B, Smith RC, Kanczler JM, Boyde A, et al. Regulation of the bone vascular network is sexually dimorphic. J. Bone Miner. Res. 2019; DOI:10.1002/jbmr.3825.

23. Tafforeau P, Boistel R, Boller E, Bravin A, Brunet M, Chaimanee Y, et al. Applications of X-ray synchrotron microtomography for non-destructive 3D studies of paleontological specimens. Appl. 2006;83(2):195-202.

24. Tafforeau P, Bentaleb I, Jaeger JJ, Martin C. Nature of laminations and mineralization in rhinoceros enamel using histology and X-ray synchrotron microtomography: potential implications for palaeoenvironmental isotopic studies. Palaeogeogr. Palaeoclimatol. Palaeoecol. 2007;246(2-4):206-227.

25. Tafforeau P, Smith TM. Nondestructive imaging of hominoid dental microstructure using phase contrast X-ray synchrotron microtomography. J. Hum. Evol. 2008;54(2):272-278.

26. Le Cabec A, Tang N, Tafforeau P. Accessing developmental information of fossil hominin teeth using new synchrotron microtomography-based visualization techniques of dental surfaces and interfaces. PloS one. 2015;10(4):e0123019.

27. Green DR, Green GM, Colman AS, Bidlack FB, Tafforeau P, Smith TM. Synchrotron imaging and Markov chain Monte Carlo reveal tooth mineralization patterns. PloS one. 2017;12(10):e0186391.

28. Mani-Caplazi G, Schulz G, Deyhle H, Hotz G, Vach W, Wittwer-Backofen U, et al. Imaging of the human tooth cementum ultrastructure of archaeological teeth, using hard X-ray microtomography to determine age-at-death and stress periods. In Developments of X-ray tomography XI. International Society for Optics and Photonics. 2017;10391:103911C.

29. Newham E. Exploring the use of tomography for the quantification of cementum growth patterns across the mammal phylogeny. PhD Thesis, The University of Southampton. 2018.

30. Le Cabec A, Tang NK, Ruano Rubio V, Hillson S. Nondestructive adult age at death estimation: Visualizing cementum annulations in a known age historical human assemblage using synchrotron X- ray microtomography. Am. J. Phys. Anthropol. 2019;168(1);25-44.

31. Newham E. Corfe IJ, Brown KR, Gostling NJ, Gill PG, Schneider P. Synchrotron radiation-based X-ray tomography reveals life history in primate cementum incrementation. J. R. Soc. Interface. 2020a;17(172):20200538.

32. Newham E, Gill PG, Brewer P, Benton MJ, Fernandez V, Gostling N J, et al. Reptile-like physiology in Early Jurassic stem-mammals. Nat. Commun. 2020b;11(1):1-13.

33. Bowen WH, Koch G. Determination of age in monkeys (*Macaca irus*) on the basis of dental development. Lab. Anim. 1970;4(1):113-123.

34. Kitchen MJ, Buckley GA, Gureyev TE, Wallace MJ, Andres-Thio N, Uesugi K, et al. CT dose reduction factors in the thousands using X-ray phase contrast. Sci. Rep. 2017;7(1):15953.

35. Zeller-Plumhoff B, Mead JL, Tan D, Roose T, Clough GF, Boardman RP, et al. Soft tissue 3D imaging in the lab through optimised propagation-based phase contrast computed tomography. Opt. Express. 2017;25(26):33451-33468.

36. Paganin D, Mayo SC, Gureyev TE, Miller PR, Wilkins SW. Simultaneous phase and amplitude extraction from a single defocused image of a homogeneous object. J. Microsc. 2002;206(1):33-40.

37. Keyriläinen J, Bravin A, Fernández M, Tenhunen M, Virkkunen P, Suortti P. Phase-contrast X-ray imaging of breast. Acta radiol. 2010;51(8): 866-884.

38. Marone F, Stampanoni M. Regridding reconstruction algorithm for real-time tomographic imaging. J. Synchrotron Radiat. 2012;19:1029-1037.

39. Nixon M, Aguado AS. Feature extraction and image processing for computer vision. Cambridge, Massachusetts: Academic Press; 2012.

40. Lanman D. Steerable Gaussian Filters (https://www.mathworks.com/matlabcentral/fileexchange/9645-steerable-gaussian-filters), MATLAB Central File Exchange. Retrieved July 16, 2021.

41. Freeman WT, Adelson EH. The Design and Use of Steerable Filters. IEEE Transactions on Pattern Analysis and Machine Intelligence. 1991;13(9):891–906.

42. Gadelmawla ES, Koura MM, Maksoud TMA, Elewa IM, Soliman HH. Roughness parameters. Journal of materials processing Technology.2002;123(1):133-145.

43. Esfahani M, Munir KS, Wen C, Zhang J, Durandet Y, Wang J, et al. Mechanical properties of electrodeposited nanocrystalline and ultrafine-grained Zn-Sn coatings. Surf. Coat. Technol. 2018;333:71–80.

44. Martinez WL, Martinez AR. Computational statistics handbook with MATLAB. Boca Baton, Florida: Chapman and Hall/CRC Press; 2015.

45. Klevezal, G., Stewart, B. S. Patterns and calibration of layering in tooth cementum of female northern elephant seals, *Mirounga angustirostris. J. Mammal.* 1994;75(2):483-487.

46. Bouxsein, M. L., Boyd, S. K., Christiansen, B. A., Guldberg, R. E., Jepsen, K. J., Müller, K. J. Guidelines for assessment of bone microstructure in rodents using micro-computed tomography. J. Bone Miner. Res. 2010;25(7):1468-1486.

47. Immel A, Le Cabec A, Bonazzi M, Herbig A, Temming H, Schuenemann VJ, et al. Effect of X-ray irradiation on ancient DNA in sub-fossil bones–Guidelines for safe X-ray imaging. Sci. Rep. 2016;6:32969.

Figure captions

Figure 1. Histological data of rhesus macaque (*Macaca mulatta*) cementum in the permanent lower first molar tooth.

(**a**) 70 µm-thick transverse reflected light digital micrograph of the cementum tissue from the coronal third of the tooth root at 20× magnification. Cementum (C) is defined as the tissue wrapping around the circumference of the dentine (D), comprising a series of circumferential increments. Blue dashed line highlights a surface scratch created during the thin-sectioning process. (**b**) Detail marked by red dashed box in (**a**) displaying cementum increments in acellular extrinsic fibre cementum, and cellular voids left by cementoblasts in cellular intrinsic fibre cementum (CIFC) under higher

resolution (50× magnification). White arrows highlight ‘light’ increments, blue arrows highlight ‘dark’ increments, and red arrows highlight cementoblasts. Red bracketed lines highlight cellular intrinsic fibre cementum. Green bracketed line highlights the hyaline layer of Hopewell-Smith, demarcating the cemento-dentine boundary. Scale bar in (**a**) represents 100 μm. Scale bar in (**b**) represents 30 μm.

Figure 2. Tomographic cementum increment counting. (**a**) Straightened, filtered PPCI SR CT image of *Macaca mulatta* cementum (see Fig. 5). (**b**) Plot of greyscale values along transect highlighted by the 10 pixel-thick coloured band in (**a**) with outer-cementum surface highlighted with orange asterisk and cemento-dentine boundary by red asterisk. Transects are split into five sections. Light/dark increment pairs are distinguished as peak/trough systems in greyscale values, where both the peak and trough depart from the mean greyscale value of that section (red line), beyond the half standard deviations of greyscale values within the section above the mean (green line) and below the mean (blue line), respectively. (**c**) Sections split into their upper and lower datasets comprising of peaks and troughs in greyscale that exceed beyond the upper and lower standard deviation (respectively). Here, peak/trough pairs are counted, denoted with red numbers. Troughs and peaks that are not counted are denoted in blue numerals, as they either do not exceed the standard deviation of that section or are less than three pixels away from the last respective peak/trough. (**d**) Resulting increment pair counts for each section as seen in (**b**).

Figure 3. SR CT scan of a tooth root and regions for signal-to-noise ratio (SNR) and contrast-to-noise ratio (CNR) calculations. (**a**) One CT slice of the tooth root of *Macaca mulatta* individual l56. Blue box highlights the ROI for

evaluation of background signal from which the mean greyscale value ($\overline{g_b}$) and standard deviation of greyscale values (σ_b) was generated for SNR and CNR calculations (see Equations (1) & (2)). The green box highlights the sampling area for cementum signal from which the mean greyscale value ($\overline{g_c}$) was generated for SNR and CNR calculations. Dashed red boxes indicate regions highlighted of the detail views. (**b**) Detail from region indicated by dashed red boxes in (**a**) from the dataset acquired at 16 mm sample-to-detector distance. (**c**) Detail from the same region from the dataset acquired at 28 mm sample-to-detector distance. (**d**) Detail from the same regionfrom the dataset acquired at 100 mm sample-to-detector distance. (**e**) Plots of greyscale values along transects indicated by dashed lines in (**b-d**), acquired at different sample-to-detector distances. (**a-d**) White scale bars in (**a**) represent 100 µm in the overview image, 30 µm in the detail view highlighted by the red dashed box, and 10 µm in (**b-d**).

Figure 4. SNR and CNR for sweep of experimental settings for PPCI through SR CT. Data shown is from specimen l56. (**a**) SNR (shown in black) and CNR (shown in red) values for different X-ray energies. (**b**) SNR and CNR values for different exposure times. (**c**) SNR and CNR values for different numbers of angular projections. (**d**) SNR and CNR values for different sample-to-detector distances. (**e**) Relationship between the standard deviation of background (σ_b – shown in black) and the mean of cementum signal ($\overline{g_c}$ – shown in red) with increasing sample-to-detector distance.

Figure 5. Image processing of PPCI SR CT images of *Macaca mulatta* cementum. (**a**) One CT slice of specimen l59. (**b**) Detail of CT slice highlighting circumferential

cementum increments (cementum defined by dashed yellow line) with midline shown as solid yellow line. (**c**) Straightened cementum section following the midline highlighted in (**b**). (**d**) Filtered image of (**c**) using a steerable Gaussian filter (see Supplement, section 1. 'Image processing by directional filtering'). White scale bar in (a) represents 200 µm; and in (b) represents 100 µm. Black scale bars in (c) and (d) represent 30 µm.

Figure 6. Robustness tests for algorithmic increment counts. (**a**) Counts (black circles) and their standard deviations (green boxes) for incremental sine wave patterns of 5 up to 30 increments with signal-to-noise ratios (SNRs) of 0.9-0.5. Inset: box displaying an example of a 10-increment pattern with an SNR of 0.5. (**b**) Counts and their standard deviations for incremental sine wave patterns of 5 up to 30 increments with SNRs of 0.4-0.2. Inset; box displaying an example of a 10-increment pattern with an SNR of 0.2. (**c**) Counts and their standard deviations for incremental sine wave patterns of 5 up to 30 increments with an SNR of 0.1. Inset: box displaying an example of a 10-increment pattern with an SNR of 0.1. Red dashed lines represent 1:1 correlation between estimated and known increment count.

Figure 7. Comparison between histological and CT data. (**a**) Detail of histological thin-section of the k49 specimen displaying 12 light increments indicated by blue arrows. (**b**) Detail of reconstructed CT slice of the same region as (**a**) displaying 11 cementum increments. (**c**) Detail of histological thin-section of the k16 specimen displaying eight light cementum increments. (**d**) Detail of reconstructed CT slice of the same region as (**c**) displaying eight increments. (**e**) Detail of histological thin-section of the l59 specimen displaying 11 light increments. (**f**) Detail of reconstructed

CT slice of the same region as (**e**) displaying 11 increments. (**g**) Detail of histological thin-section of the l56 specimen displaying 12 light increments. (**h**) Detail of reconstructed CT slice of the same region as (**g**) displaying 11 increments. See Supplementary Figure S4 for combination of histological/tomographic data for each specimen. Black scale bars represent 30 µm. Yellow whiskers highlight the granular layer of Tomes (labelled GLoT); pink dashed whiskers highlight the hyaline layer of Hopewell Smith (labelled HLHS); and red arrows highlight cellular voids within cellular intrinsic fibre cementum. Red dashed circles highlight surface damage created during thin-section processing.

Figure 8. 3D CT data of cementum increments. Data shown is from specimen k49. (**a**) Straightened and filtered CT slice displaying 10 increment pairs, highlighted with coloured arrows. (**b**) Schematic of detail from **a** highlighted by dashed red box, showing complexities in increment patterns. Increments are given the same colour as their respective arrows in **a**. Instances of splitting are highlighted with dashed red lines, and instances of coalescence by blue dashed lines. (**c**) 3D model of segmented cementum increment patterns plotted through the coronal third of the root imaged by PPCI through SR CT. Scale bars represent 100 µm.

Figure 9. Comparison between known increment pair counts and those estimated by the proposed cementum increment counting algorithm. PPCI SR CT data shown are from m1 teeth of ten *Macaca mulatta* individuals of known age (see Table 1**).** Cyan boxes indicate the interquartile range around the mean estimated increment count, indicated by the thick black line. Whiskers represent the extreme lower and upper estimated counts. Blue circles indicate known age of each individual. Red

triangles indicate the maximum and inverted green triangles indicate the minimum expected increment count for each individual based on the approximate age-of-eruption of the m1 at 18 months of age in *Macaca mulatta*.

Table 1.

Specimen	Age (years)	DOB	DOD	Known increment pair count		Estimated increment pair count	
				Minimum	Maximum	Mean	Standard deviation
**k49**	12	09.04.03	08.04.15	10	11	10	0.95
**k91**	11.5	06.10.03	10.04.15	9.5	10.5	10	0.81
**k23**	12	09.03.03	09.04.15	10	11	10	0.89
**k24**	12	12.03.03	08.04.15	10	11	10	0.96
**l10**	11	20.02.04	08.04.15	9	10	9.5	0.94
**l14**	11	26.02.04	10.04.15	9	10	9.75	0.86
**l56**	11	14.04.04	10.04.15	9	10	10	0.92
**l59**	11	17.04.04	09.04.15	9	10	9.5	0.71
**k16**	10.5	16.09.04	09.03.15	8.5	9.5	9	0.52
**t46**	5.5	11.03.10	07.07.15	3.5	4.5	5	0.94

Table 1. Life history data, known increment pair counts and estimated increment pair counts for each of the 10 female rhesus macaque (*Macaca mulatta*) individuals studied. DOB = date of birth; DOD = date of death. Mean increment pair counts are rounded to the nearest 0.25 years for comparison with known increment pair counts.

Table 2.

**Scan name**	**Energy (keV)**	**Exposure time (ms)**	**Number of projections**	**Sample-to-detector distance (mm)**	**Mean SNR**	**Mean CNR**
SO20	19	150	1501	14	187.9	50.7
SO21	20	150	1501	14	123.8	56.9
SO22	21	150	1501	14	112.9	60.8
SO23	22	150	1501	14	82.6	35.5
SO24	26	150	1501	14	72.0	24.5
SO25	20	100	1501	14	118.3	50.7
SO26	20	125	1501	14	124.0	55.4
SO27	20	300	1501	14	177.8	72.3
SO28	20	150	3001	14	245.4	74.5
SO29	20	150	4501	14	252.7	81.5
SO30	20	150	6001	14	300.5	84.9
SO31	20	150	1501	16	142.2	59.0
SO32	20	150	1501	20	152.9	68.9
SO33	20	150	1501	28	185.6	75.2
SO34	20	150	1501	60	179.6	77.8
SO35	20	150	1501	100	247.4	54.9

Table 2. Image quality assessments of PPCI SR CT images using different experimental settings. SNR = signal-to-noise ratio; CNR = contrast-to-noise ratio.

Response to the Academic Editor:

We thank the editor for their positive review of our article and offer for revision and resubmission. We concede that comparison with the results of simila[illegible]udies on archaeological and fossil teeth suggest that the damage observed in our experiment was most likely due to suboptimal preparation of teeth prior to scanning. We have adjusted our discussion of this accordingly, to minimise any inference that synchrotron imaging itself is necessarily destructive.

We have revised our citation format and reference list to both conform to that of PLoS ONE, and amend inconsistencies in the reference list. We have also revised our scale bar description and scaling in all figures. Finally, following the comments of the editor and reviewers, we have aimed to make the narrative more broad in relation to cementum biology and synchrotron radiation-based X-ray imaging.

We now include the statement "No permits were required for the described study, which complied with all relevant regulations" to conform to PLoS ONE's reporting requirements.

We also now explicitly state what funding from Ginko investments was used for (Line 665: "Funding was also provided by Ginko Investments Ltd (Bristol, UK) for purchasing external hard drives and funding travel to the Swiss Light Source, and the Academy of Finland. And on line 673: "The funders had no role in study design, data collection and analysis, decision to publish, or preparation of the manuscript." We also amend our competing interest statement as: "The authors report no conflicts of interest. The private funding received from Ginko Investments Ltd does not alter our adherence to PLOS ONE policies on sharing data and materials."

We hope that the changes and revisions to our manuscript are satisfactory.

Response to reviewer 1:

Reviewer #1: This is an important contribution to the literature on cementum. It defines the nature of an annual increment in acellular cementum and describes a semi-automated method for making objective quantitatively based counts of annual cementum increments. It is significant that these counts closely match those made with traditional transmitted light microscopy in the same specimens.

We thank the reviewer for their considered and positive review.

The study is based on animals that were raised under laboratory conditions (presumably caged for most of their lives). Since this suggests they were buffered from the external seasonal environment and since the presumption is also that their diet was of a consistent nature throughout their lives, the presence of annual increments in their cementum suggests this is an endogenous rhythm and not one that has been assumed by some to be entirely driven by seasonality and or shifts in dietary quality and/or toughness. Some reflection of this in the discussion might be useful for people to at least ponder the implications.

We agree that the above-mentioned factors point to the circum-annual periodicity of cementum incrementation following an endogenous, potentially hormonal rhythm. We have thus altered our discussion to include (Line 567): "This is of particular note given that the study sample originates from a captive population with minimal seasonal environmental and nutritional variation. It is understood that the cementum of captive mammals presents lower increment contrast than free-roaming populations (Klevezal, 1995), and so the identification and accurate counting of circum-annual increments of PPCI data in captive cementum may be another promising element of this technique. This also adds to the body of work finding circum-annual increments in modern humans (Naji et al., 2016 and references therein), suggesting that incrementation follows an endogenous (potentially hormone-driven; see Newham et al., 2020a) rhythm as opposed to primarily reflecting seasonal dietary differences (Liberman, 1993)."

The age range of the animals in this study is as expected much less than the age range in studies of adult modern human cementum. It seems the accuracy of counts of annual cementum increments decreases with increasing age. This may imply later cementum increments, formed say after twenty of thirty years (way beyond the ages of animals in this study), are of a different nature, perhaps more likely fused together or so much slower in formation rate that they can't be resolved. It might be a good thing to offer some suggestions about the problems this study may not have been able to address as well as those it clearly does address so well and that are discussed. In this way other potential factors affecting, particularly large cementum increment counts in older humans, are less likely to be swept under the carpet and more likely to be addressed in other ways. I'm particularly happy to see Zander and Hurzeler (1958) making it back into the cementum literature. It is one of very few studies that contains data on rates of cementogenesis in different modern human tooth types over many years.

While several validation studies have found exceptional accuracy using cementochrono[illegible] to age humans living up to and above 90 years of age (Kagerer and Grupe, 2001; Witwer-Backhofen et al., 2004), others have indeed shown a decrease in accuracy with increasing age (Mani-Caplazi et al,, 2019; see Naji et al., 2016 for a review). It is known that the cementum of long living animals including humans shows an increasing increment density (i.e. numer of increments per unit length), with increment count increasing faster than increases in radial width of the cementum. This increase in increment density evidently makes subjective determination of increments more difficult, given a certain spatial resolution. Thus, it may well be that the optimum parameters found for our sample will be suboptimal for others. Several synchrotron beamlines can now readily achieve effective voxel sizes of around 0.3 µm, which may be preferable for distinguishing densely spaced increments in human cementum. As we state (now in more detail; see Line 537) in our discussion, we believe our results indicate that each experiment based on novel material to cementum SR CT imaging should begin with a parameter sweep to achieve the optimum data relative to the goals of the particular experiment, including the biological nature of the sample itself.

The materials and methods are very carefully described and left and right 'm1s' – better to say M1s because they are permanent teeth – were fixed for 10 days in paraformaldehyde. Can the authors present some evidence that there was no demineralisation from the surface inwards as a result of this that might have introduced (or altered) a gradient in the grey scale values e.g. in Figure 2?

We cannot conclusively state whether any demineralisation occurred due to fixation in PFA, and this m[illegible]y be one of the factors that led to microscale cracking witnessed during the experiment (Figure S5). Preparation of the sample and its effects on imaging are now discussed in the revised manuscript (Line 598). For future work, we intend on dehydrating all teeth through desiccation prior to scanning.

References:

- Condon K, Charles DK, Cheverud JM, Buikstra JE. Cementum annulation and age determination in Homo sapiens. II. Estimatres and accuracy. AM. J. Phys. Anthropol. 1986;71:321-330.
- Kagerer P, Grupe G. On the validity of individual age-at-death diagnosis by incremental line counts in human dental cementum. Technical considerations. Anthropol. Anz. 2001;59:331-342.
- Mani-Caplazi G, Hotz G, Wittwer-Backofen U, Vach W. Measuring incremental line width and appearance in the tooth cementum of recent and archaeological human teeth to identify irregularities: First insights using a standardized protocol. Int J Paleopathol. 2019;27:24-37.
- Naji S, Colard T, Blondiaux J, Bertrand B, d'Incau E, Bocquet-Appel JP. Cementochronology, to cut or not to cut? Int. J. Paleopathol. 2016;15:113-119.
- Wittwer-Backofen U, Gampe J, Vaupel JW. Tooth cementum annulation for age estimation: results from a large known-age validation study. Am. J. Phys. Anthropol. 2004;123:119-129.

Response to reviewer 2:

Thank you for asking me to review the work by Newham and colleagues.

This piece of research is most valuable and represent a considerable step forward in the field of cementochronology,with potential implications for studying hominin fossils, yet with some limitations that I will highlight later in this review.

I would like to congratulate the authors for the work and encourage them to consider the comments andsuggestions I made, after what I will fully support the publication of this work in PlosOne.

I have provided a detailed annotation of the main manuscript (attached pdf). Please find below some more generalcomments:

We thank the reviewer for their extremely considered and thorough review. We appreciate their positive interpretation of the value of our study, a[illegible]ave taken on board their critiques. We have thus considerably revised the elements raised by the reviewer, including new supplementary study and modification of discussion points that were rightfully highlighted as easily misinterpreted. We have gone through the detailed comments in the reviewers attached commented PDF, and attempted to correct all points accordingly. Please see below for direct replies to comments:

Abstract

- see my comment, the claim are currently not supported by any reported statistical results. Please refer to yourcorrelation tests.

We have now changed the claim to “qualitative comparison” and have created a new figure in the Supplement (Figure S3), where histological and tomographic data are combined to provide qualitative evidence for this statement.

- After reading in depth the paper and SOM, I understand why there is no highlight about the non-destructive natureof synchrotron µCT imaging, this is later explained by the destructive protocol used. Yet, for future directions, it would be good to stress that non-destructive imaging is possible.

We have added a note in the abstract that synchrotron CT imaging is in general non-destructive, given appropriate preparation of the sample prior to scanning.

Introduction

- errors in biblio

We have adjusted errors and the citation style accordingly.

- lack of precision regarding location of AEFC and CIFC.

We have added more detail on current knowledge of cementum tissue distribution (see line 79), although it should not be expected to be homogeneous (Naji et al., 2016).

- improve clarify regarding the use of the word "cementum": complex tissue with sub-types, indicate here clearlywhat is being studied.

We have clarified that we are specifically interested in imaging and analysing AEFC.

Methods + Results

- the protocol used here is highly destructive! The crown was sectioned, and the roots separated. The end of the introduction involves a kind of blunt criticism about the pilot study of Le cabec et al 2019, yet, they did not cut theteeth, and use higher energy to avoid damaging the DNA!

The preparation procedure is indeed more destructive than that of Le Cabec et al. (2019), because one of our principal aims was validating the optimum experimental settings to achieve optimal image quality for PPCI of cementum, regardless of specimen provenance or the broader scientific goals. This meant ensuring that samples were processed to be optimal in shape for PPCI, with minimal “excess” tissue interfering with the X-ray beam outside of the region of interest being scanned. We now implicitly discuss these concerns in the paragraph starting at line 191.

Of course, this procedure is unsuitable for many archaeological/fossil specimens, and different experimental settings are necessary for maximal possible image quality while minimising X-ray dose (if a DNA analysis is planned following PPCI) and accounting for interference by other roots etc. Briefly, our focus was to push the

imaging technique for best possible image quality that could be achieved at TOMCAT beamline. We now discuss the other constraints that will effect 'optimum' experimental settings for a particular cementum study from Line 539.

We apologise for our seemingly blunt review of Le Cabec et al. (2019), as this was definitively not our intention. We value the work of Cabec and co-workers as one of the founding studies for the synchrotron study of cementum and have revised our wording accordingly (Line 151: "Due to the uniqueness of their archaeological sample, the canines studied in Le Cabec et al. [30] could not be thin-sectioned."). We also believe that our comparison with the current gold standard of thin-section histology is a necessary prerequisite for understanding the accuracy of PPCI data how they reflect genuine biological structures, and the effects of taphonomic and/or diagenetic processes on this structure.

- Whether the teeth used here are deciduous or permanent is not clear until the discussion. This should be stated atthe beginning of the "Materials".

The teeth used are all permanent teeth, and we apologise if we did not make that sufficiently clear throughout. We have now added reference to this more frequently and from the start of the "Materials" section.

- There are no explanation about how the thin sections were made? Any polishing involved? Slice thickness?

The full thin-section processing procedure is available in Newham et al. (2020). We have also added the slice thickness in our revised manuscript.

- SR µCT acquisitions: Why choosing 1501 projections whereas SNR seems best around 3000-4000 projections?

We used 1501 projections for the principal experiment in order to scan the entire sample within the allotted experimental time. This compromise is now discussed in terms of general PPCI study of cementum in the revised discussion: (Line 551 "Further, while increasing the number of angular projections increases image quality, it also increases the total acquisition time of each CT scan. As each synchrotron experiment is awarded a restricted amount of beam time, optimal experimental setings have to be weighed up against scanning time to image a sufficient sample of specimens. Hence, given the time allocated for our experiment, we retained 1501 projections per CT scan, half of the optimal value around 3000-4000 in terms of SNR (Fig. 4), in order to image our complete sample within the allotted beamtime".

- There is a lack of explanation regarding what has been scanned: where is the FOV/ROI on the root? What size? Which root? Where along the root length? Was the ROI chosen in a standardized manner for all individuals? Explainthe criteria. A figure would be helpful.

We have now explained that scans were centered on the coronal third of the anterior root of each tooth analysed. Three regions were scanned, which were overlapping longitudinally and covering a volume of approx. $4\times1.3\times1.3$ mm^3. See Line 248 for this explanation.

- Figure 2: I think there is a mismatch between the x values in Fig 2b and Fig 2c? (if you track the peaks, andcorresponding x-values).

Many thanks for highlighting this mistake, which is now corrected.

- Cite more often your supplementary information.

The supplementary information is now cited in seven more places than the original manuscript.

- 16 or 8 bit data? This needs to be clarified and tested as it may have an impact on the results.

The original CT reconstructions were saved as 16-bit files, but the output of the steerable Gaussian filter function was saved as 8-bit files, and the increment counting procedure was developed for 8-bit data.

- Figure 3: on the plot, there are too many curves compared to the figure legend? Also rather indicate the resolution / pixel size and not the sample-detector distances, as this is less intuitive for most non-specialist readers.

The plot and key represent the different sample-to-detector distances examined, as shown in Figure 5.e. We use the actual distance between the sample and the detector, rather than the effective propagation distance that would require further explanation in the manuscript. We believe that the actual distance is an effective concept for the unexperienced reader that requires minimal knowledge or explanation of how the two factors (physical distance and effective pixel size) interact, especially when the effects of this distance are shown in Figure 3. Finally, due to the quasi-parallel nature of the synchrotron X-ray beam, effective propagation distance is nearly identical to sample-to-detector distance (Zeller-Plumhoff et al., 2017)

- How do the method deal with second-order increments if any? In the SOM I see they are ignored, as well as on

thefigure showing the I and II in light blue. But is it so frequent? I might be good to show a picture, because those structures do exist. They have been identified also by SXRF.

The method discounts increments with image contrast that does not exceed the local standard deviation of the image grey scale (i.e. “piggy-back features” as shown in Figure 5). Howe[illegible] the method does not directly discriminate between “real” circum-annual increments and “secondary” sub-annual increments. This would require subjective definition between increment types in each slice examined, which in-turn relies on user experience and interpretation. Our goal is instead to be as objective as possible, and to minimise the reliance of cementochronology on user experience (see Line 380: “While this procedure does not necessarily target or discriminate “accessory” or secondary increments from “real” circum-annual increments, it remains an objective way of discerning “real” increments that does not rely on subjective interpretation by the user. This in-turn relies on user-experience, which is one of the primary elements of cementochronology we are here seeking to minimise.”.

We understand from previous studies (Newham et al. 2020a,b) that secondary increments commonly form due to intermittent splitting of circum-annual increments, which we have attempted to overcome by intensively sampling over each examined slice, and over 30 slices per scan.

- how were the 30 slices chosen within the stack? Or was the ROI chosen to involve 30 slices only? This is not clear.

We apologise that we did not make that clear enough. The 30 slices were chosen within regions with highest image contrast for increments upon manual examination of each scan. For each region, slices were selected until 30 slices had been chosen (for each scan). We have made this clearer in our revised manuscript accordingly (line 419: “Slices were selected contiguously through these regions until a number of 30 slices had been reached”).

- any use of thick slices (average of multiple slices to enhance visibility)? Or only native slices?

Native slices were[illegible]d.

Discussion

Lines 553-560: + Fig. S3: the scans generated micro-cracks: this is of concern!!! Maybe the specimen was still fresh andnot totally dry? this could have induced the liberation of free radicals, especially with the low energy used.

This claim is actually going to be harmful to the synchrotron community, and a major concern! Why? Because ***non-specialists are going to be comforted in the <u>general belief</u> that synchrotron imaging damages specimens***. So it should be made clear here why and how these micro-cracks occurred, and *not let readers believe this will happen every time*!

We apologise for this unanticipated interpretation of this discussion. By no means we intended to undermine confidence in synchrotron imaging, and have significantly modified our discussion to highlight that the preparation of the sample is the primary cause for microstructural damage, exemplified by comparison with data from archaeological (Le Cabec et al., 2019) and fossil (Newham et al., 2020a) data, Line 608: “It also became apparent during scanning that micrometre-scale cracks, which are not visible macroscopically, have formed within the cementum tissue (Supplementary Fig. S5), most probably due the interaction of the hard X-rays with residual water left within the teeth and/or related effects due to this interaction [47]. Although this damage could not be seen macroscopically, comparison with the lack of observations of any damage in archaeological [30] and fossil [32] teeth confirms that the preparation of “fresh” teeth was suboptimal for SR CT scanning, as residual water was retained. Further, it cannot be confirmed whether micro-cracks occurred solely during the scanning process, or started due to demineralisation during fixation in paraformaldehyde. This should be examined in future studies via microscopic examination of the cementum of fixed teeth. Moreover, further optimisation of the preparation procedure for fresh teeth is still needed, that ensures both minimal risk of biological contamination and minimal damage to the tissue during CT scanning.”

Supplementary material

Equations 2 and 3: is the second 0 the symbol for degree (0°)? then it should be superscript to 0 or 90, here it seemsto have normal size and it thus seems confusing. Or L42, explain this is for 0°, this would help non-specialists to understand the notation.

This font has now been changed accordingly.

Fig. S2 and others: individual plots should at least have 1 or 2 key words as title otherwise it is impossible what they represent without reading the full caption.

Titles have been added to plots as suggested. Thank you very much for this useful comment.

L66: so why not running this on the 16 bit data? It needs to be tested if running it on 8 bit induces a loss ofcontrast/details that would not occur when using 16 bit data.

The output of the filtering function is first calculated at double precision (i.e. "float64" between 0 and 1) format, then converted to the chosen Tiff format (here as 8-bit data). As this conversion is proportional to the distribution of float64 values in the original filtered file, conversion to 16-bit data will experience the same (relative) greyscale distribution as that of an 8-bit conversion, although the absolute greyscale variation will be different. However, we have now run a test on a subsample of five straightened datasets converted to both 8-bit and 16-bit data and subjected to our increment counting algorithm. This test shows that both bit depths produce comparable accuracy of estimated increment counts when compared to expected counts for each specimen. ANOVA comparisons between 8-bit and 16-bit datasets for each specimen only suggest a significant difference in increment counts for one single specimen, and Levene's test show no significant difference in the variation between 8-bit and 16-bit datasets for each specimen. We interpret these results as suggestive that, while a minority of specimens may produce different ranges of absolute increment estimate values using 8/16-bit data, they are still centred on the same increment count and 16-bit data does not provide significantly higher accuracy or precision for increment count estimates than 8-bit data.

L78-79: I am not sure to understand how you choose by which integer you divide the 1st directional derivative image.

The optimum integer was chosen as the integer that provided the strongest image contrast between circum-annual cementum increments, while preserving as much greyscale contrast as possible. This was based upon qualitative examination of data filtered using each integer value successively, and monitoring when particular elements of the data were lost. While this is a subjective process, it was necessary to determine that as much data of interest (circum-annual increments) was preserved as possible, while minimizing the presence of greyscale noise.

L104: is not this circular reasoning? the PPC SR data without any processing, thus including background noise? this needs to be clarified.

While filtering aims to reduce background noise (i.e. unwanted signal), over-filtering can also remove potentially important and useful biological contrast (i.e. wanted signal). Thus, comparison between filtered data and original data was important to monitor what information (wanted vs unwanted signal) was being lost when applying increasingly strong filters, and to establish a cut-off where too much "wanted" biological signal was lost.

L161: why "tribological" in the title??? and L187, L286: I don't understand the use of this word "tribological"? To me,this has to do when 2 surfaces are in contact and involve movement (e.g., tooth occlusal surfaces and food items).

We agree that this term is confusing and have deleted the term throughout the manuscript.

Table S5: typo.

Typo corrected. Thank you very much for the detailed review, which is greatly appreciated.

References:

- Naji S, Colard T, Blondiaux J, Bertrand B, d'Incau E, Bocquet-Appel JP. Cementochronology, to cut or not to cut? Int. J. Paleopathol. 2016;15:113-119.
- Newham E, Gill PG, Brewer P, Benton MJ, Fernandez V, Gostling N J, et al. Reptile-like physiology in Early Jurassic stem-mammals. Nat. Commun. 2020a;11(1):1-13.
- Newham E. Corfe IJ, Brown KR, Gostling NJ, Gill PG, Schneider P. Synchrotron radiation-based X-ray tomography reveals life history in primate cementum incrementation. J. R. Soc. Interface. 2020b;17(172):20200538.
- Zeller-Plumhoff B, Mead JL, Tan D, Roose T, Clough GF, Boardman RP, et al. Soft tissue 3D imaging in the lab through optimised propagation-based phase contrast computed tomography. Opt Express. 2017;25(26):33451-68.
