# Supplementary material for: A robust, semi-automated approach for counting cementum increments imaged with synchrotron X-ray computed tomography
Source: PLoS One. 2021 Nov 4;16(11):e0249743. doi: 10.1371/journal.pone.0249743 (PMC8568193; doi:10.1371/journal.pone.0249743)
Supplement: Attachment — Submitted filename: Reviewer response 16.09.docx [file pone.0249743.s006.docx]

Reviewer #1: I congratulate the authors on a very well written paper
I am satisfied all the comments and suggestions raised previously have been dealt with

We thank the reviewer for their previous comments to improve the manuscript, and positive response to our paper.

Reviewer #2: I would like to congratulate the authors on their work for revising their manuscript, which has greatly improved. I support the publication of this work after some minor comments have been addressed. I have again annotated in details the pdf (text with changes in red, + figures + your answers to the reviewers), and here is a summary of my comments:
We firstly thank the reviewer again for their detailed comments of our revised manuscript, which we have endeavoured to follow and correct accordingly. We also thank them for their positive summary of the revision.

Title:
- An important aspect that I missed during the first round of review: the title needs to contain either "synchrotron" or "phase contrast", since most readers would believe that, with a Skyscan (conventional µCT scanner) and scanning in absorption mode, they could visualize and reliably identify cementum increments, which is not the case. It should be made clear that this is enabled by synchrotron imaging, and more specifically PPC µCT.

We have now changed the title to: “A robust, semi-automated approach for counting cementum increments imaged with synchrotron X-ray computed tomography”.

Abstract:
- L38: the authors present the difficulties encountered by previous studies facing the identification of primary vs secondary cementum increments. Yet, no difference is made in the present study during increment identification (actually this is not so clear: it is identified then discarded, therefore the method seems to be able to make the difference). I just want to draw your attention on the fact that this would need to be discussed as this has been presented as a weakness/difficulty of previous studies, which can considerably affect the reliability of increments counts.

We have now amended the identified portion of the abstract to read: (line 45) “PPCI allowed the qualitative identification of primary/annual versus intermittent secondary increments formed by splitting/coalescence. A new method for semi-automatic increment counting was then integrated into a purpose-built software package for studying cementum increments, to count increments in regions with minimal complications.”

Main text:
- bias in sample: only juveniles? See my comment in the previous round of reviews. The algorithm may work better indeed on young individuals, but may be challenged on older individual, whose cementum may have undergone remodeling to some extent, or have more packed increments with less clear borders.
All teeth studied were permanent teeth, as macaques replace their m1 teeth at <18 months of age. Only one individual was a juvenile/young adult (t46; 5.5 years at death), and the nine other individuals were adults. However, while our macaque sample does not present a juvenile bias, the absolute lifespans experienced by our sample may provide a low lifespan bias for the validation results of our increment counting technique, when compared to potential results for long-living mammals including humans. As you say, such samples will present a different challenge than our sample for both imaging and analysing cementum increments, and will require their own optimisation and validation procedures for these tasks. This is a major element of our discussion and conclusion, and we look forward to testing these potential differences, and further optimisation procedures to provide as accurate estimates as possible for increment counts using synchrotron CT and image processing.

- “juvenile cementum”, “adult cementum”: I recommend changing this very misleading phrasing as it actually refers to whether the individual is juvenile or adult (and within this whether this is a young or old adult).
We agree that this terminology is too vague and have modified our new MS to be more specific: (line 515) “Cementum in juvenile individuals, deposited prior to sexual maturity, has been previously shown to contain more complex incrementation and greater amounts of increment splitting and coalescence than cementum deposited during sexual maturity [45].”

- use the multiply sign instead of the letter “x”.

Now changed to multiply sign ” ×”

- typo for ref. 30 and work by Newham et al.
All typos now corrected according to detailed PDF notes of MS. We thank the reviewer for their thorough checking!

- L596: this sentence is misleading: PPC SI does not allow to see the whole of the cementum at 0.66µm at once. For non-expert readers this would provide wrong hopes about what can be achieved.

We have now corrected this mistake, changing the passage to (Line 596): “The volumetric nature of CT datasets allows navigation through the entire scanned portion of the cementum tissue at an isotropic and sub-micrometre nominal spatial resolution.”

- Fig S3: a comment is left by one of the co-authors.
Comment now removed.

- Fig. S4: very convincing!
We thank the reviewer for their suggestion for this figure and its added support.

I could not find any feedback in the Authors’s answers regarding my question whether the color of the specimen had changed after scanning, and whether this color change was reversible or not? This is important to report and state clearly. Works at the ESRF had been strongly criticized for changes in the color of the enamel (on fossil and archeological teeth) due to the color center effect. Tafforeau (2008) had showed that exposure to UV light of appropriate wavelength (or even day light but it takes longer) enables recovering the initial state of the sample.

We apologize for our omission of a response to this query. The samples did turn a slight pink colour in the regions that encountered the X-ray beam. This lasted a few hours but colour returned to normal after a few days. We appreciate the information and advice regarding UV light and will consider this technique for future experiments. This is now noted in the manuscript (Line 631): “A short-term discoloration of specimens was also noted, with a slight pink hue created within the scanned portion of tooth roots immediately following scanning. This discoloration lasted typically six-eight hours, before the original colour returned.”
